# Hotspot pocket-based discovery of urea transporter selective inhibitors

**DOI:** 10.1038/s41467-026-71834-w

**Published:** 2026-04-20

**Authors:** Lei Liu, Zhi Li, Chao Zhang, Yan Zhang, Zhizhen Huang, Daolai Zhang, Dongfang Li, Juanjuan Zhao, Yuhao Miao, Boyang Cai, Kongkai Zhu, Jin-Peng Sun, Guige Hou, Ying Sun, Baoxue Yang, Xiao Yu, Shenming Huang

**Affiliations:** 1 State Key Laboratory of Bioactive Molecules and Druggability Assessment, Guangdong Basic Research Center of Excellence for Natural Bioactive Molecules and Discovery of Innovative Drugs, Jinan University, Guangzhou, China; 2 Advanced Medical Research Institute, Cheeloo College of Medicine, Shandong University, Jinan, Shandong China; 3 The Second Qilu Hospital of Shandong University, Jinan, China; 4 Jiangsu Key Laboratory of Geriatric Precision Medicine and Aging Intervention, Xuzhou Medical University, Xuzhou, Jiangsu P. R. China; 5 Institute of Brain Science and Brain-inspired Research, Shandong First Medical University & Shandong Academy of Medical Sciences, Jinan, Shandong PR China; 6 School of Pharmacy, The Key Laboratory of Prescription Effect and Clinical Evaluation of State Administration of Traditional Chinese Medicine of China, Shandong Medical and Pharmaceutical University, Yantai, China; 7 Department of Pharmacology, School of Basic Medical Sciences, State Key Laboratory of Natural and Biomimetic Drugs, and State Key Laboratory of Vascular Homeostasis and Remodeling, Peking University, Beijing, China; 8 Department of Physiology and Pathophysiology, School of Basic Medical Sciences, State Key Laboratory of Vascular Homeostasis and Remodeling, Beijing Key Laboratory of Cardiovascular Receptors Research, Peking University, Beijing, China; 9 Medical Innovation Center (Taizhou) of Peking University, Taizhou, China; 10 Key Laboratory Experimental Teratology of the Ministry of Education and Department of Physiology, School of Basic Medical Sciences, Shandong University, Jinan, China

**Keywords:** Structural biology, Virtual screening, Virtual drug screening, Cryoelectron microscopy

## Abstract

Urea transporter (UT) inhibitors are a promising class of diuretics, as selective inhibitors targeting UT-A subtypes have demonstrated considerable therapeutic potential. Herein, we employ a two-round progressive hotspot pocket-based virtual screening approach combined with biological validation to identify M353-0039 as a highly potent and selective inhibitor of UT-A2. We conduct cryo-electron microscopy to solve the structures of UT-A2 bound with the two inhibitors, M353-0039 and E822-1968, at the resolution of 2.7 Å and 2.9 Å respectively, and elucidate the structural mechanism underlying the superior efficacy and selectivity of M353-0039. Compared with the inhibitor HQA2 and E822-1968, M353-0039 occupies a deeper binding pocket and forms more interactions with UT-A2, thus leading to greater inhibitory potency. We demonstrate that the selectivity of M353-0039 is driven by the nonconserved residues C285 and G322 within the “T-T” subpocket of UT-A2. Finally, we validate the selective effects of M353-0039 in inhibiting UT-A2 function both in mouse models and hepatic cell. These findings not only identify a selective inhibitor as a tool that can be applied to elucidate the unique physiological roles of UT-A2 but also provide an available method for efficiently developing UT-A-selective inhibitors with potent activity as the next-generation diuretics.

## Abstract (web summary)

In this work, researchers identify a selective inhibitor of the urea transporter UT-A2 using hotspot pocket–based virtual screening combined with cryo-EM and pharmacological methods, thereby providing an efficient approach for developing UT-A-selective inhibitors as next-generation diuretics.

## Introduction

Urea represents the terminal metabolite of nitrogenous waste in humans and animals, ranking among the most prevalent organic compounds in biological systems. In mammalian physiology, urea homeostasis is predominantly regulated by a family of integral membrane proteins termed urea transporters (UTs), which orchestrate critical biological processes in establishing the osmotic gradients essential for renal urine concentration and maintaining systemic nitrogen balance while coordinating with other vital physiological functions^1–3^. Mechanistically, these UT proteins facilitate unidirectional urea movement across cellular membranes through facilitated diffusion driven by concentration gradients, positioning them as indispensable components of the renal medullary concentrating mechanism^4,5^.

Mammalian UTs arise from two tandemly organized genes: S*LC14A1 and SLC14A2*^6^. The S*LC14A1* gene produces two UT-B isoforms, UT-B1 and UT-B2, which share identical protein sequences except for a 55-amino acid N-terminal extension unique to UT-B2. In contrast, the *SLC14A2* gene encodes a family of six UT-A isoforms (UT-A1 to UT-A6), with species-specific expression patterns: UT-A1, UT-A2 and UT-A3 are conserved across mice, rats, and humans, whereas UT-A4 is rodent-specific, UT-A5 murine-exclusive, and UT-A6 uniquely expressed in human colon^7^. UT-A1 represents the largest UT protein, with the other UT-A isoforms sharing extensive amino acid homology with UT-A1, suggesting their evolutionary derivation from proteolytic processing or alternative splicing of this ancestral transcript.

UT-A1-3 and UT-B exhibit renal-specific expression where their pivotal roles in the urinary concentrating mechanism have been extensively characterized^1,8–11^. Pharmacological inhibition of UTs using selective inhibitors has been experimentally validated in animal models to suppress renal urine concentration while inducing osmotic diuresis without disrupting electrolyte homeostasis^12,13^. This unique diuretic profile positions UT inhibitors as promising therapeutic agents for managing refractory edema unresponsive to conventional loop diuretics such as furosemide^12^. Furthermore, their diuretic properties enable synergistic combinations with other diuretics in treating hyponatremia associated with syndrome of inappropriate antidiuretic hormone secretion (SIADH) and edematous conditions linked to congestive heart failure, nephrotic syndrome, and cirrhosis^14^. Notably, while UT-A expression remains predominantly renal, UT-B exhibits extrarenal distribution in multiple organs including the brain, small intestine, colon, vascular endothelium and testes^15^. Phenotypic analyses of UT-B and UT-A knockout mice have uncovered some different roles besides the urine urea concentration in kidney. Cardiac conduction defects were found in mice deficient in UT-B^16,17^. These findings highlight potential off-target effects and extrarenal complications arising from non-selective UT inhibition^18,19^. Structural analysis reveals that UT-B exhibits ~40% sequence difference with UT-A2^8^, suggesting that their sequence divergence likely underlies distinct functional specializations. Importantly, these differences offer an opportunity for developing selective inhibitors of UT-A2 to avoid the off target extra-renal effects from the non-selective UT inhibitors that can block both UT-A and UT-B. Therefore, therapeutic strategies targeting UT-A with high selectivity may preserve the diuretic efficacy of UT blockade while mitigating risks associated with extrarenal UT-B modulation, and offer superior clinical translational potential. Unfortunately, despite extensive research efforts, inhibitors that combine complete UT-A selectivity with potent inhibitory activity remain unreported.

**Fig. 1:**
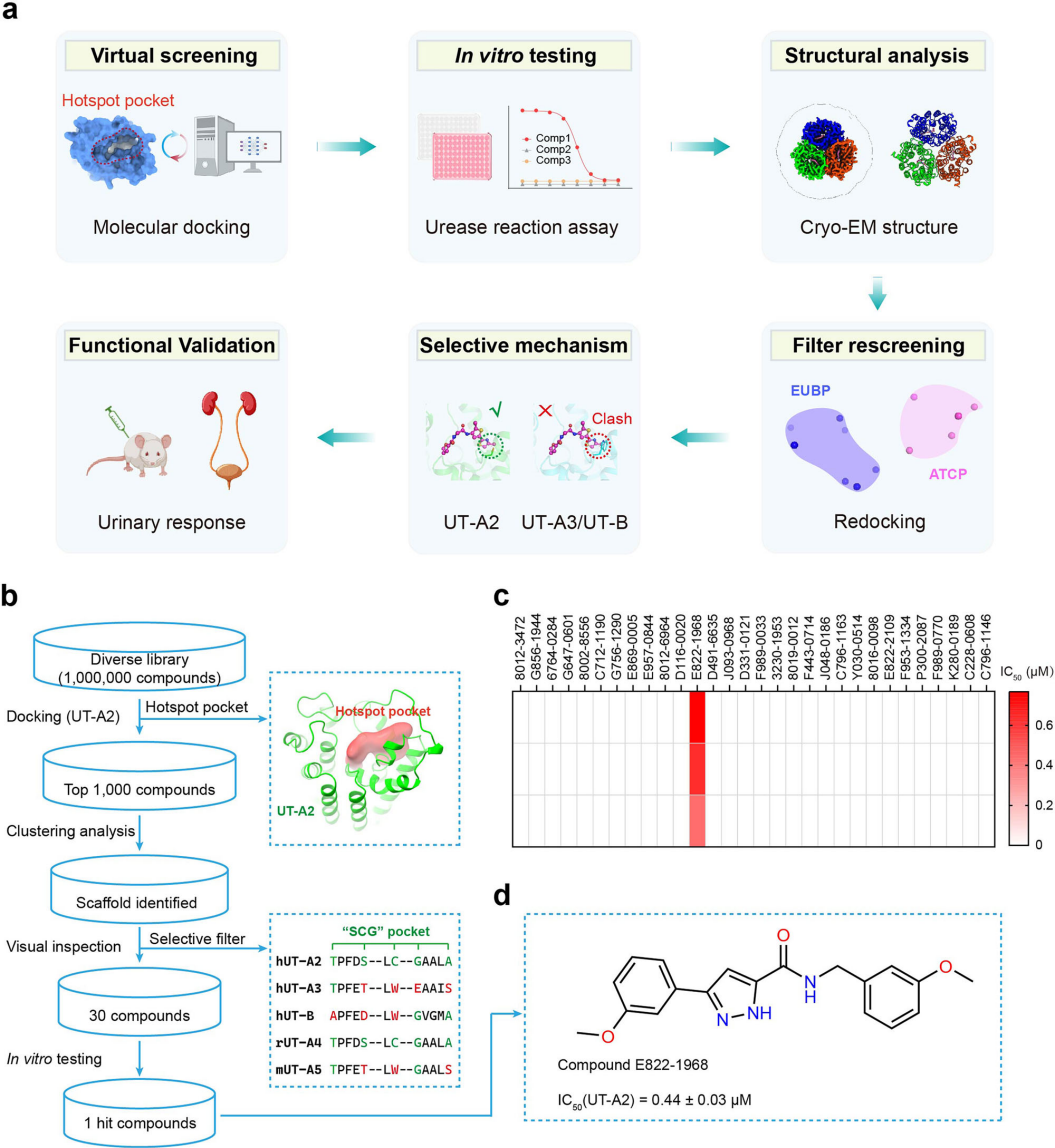
Hotspot pocket-based virtual screening of selective inhibitors to UT-A2. **a** A schematic illustrating the overall research flow of this paper. **b** The virtual screening strategy of selectively UT-A2 inhibitors discovery. Approximately one million compounds were used for screening; the top 1000 (by docking score) were clustered, then 30 compounds were selected manually according to compound-SCG pocket interaction profiles. These molecules were further evaluated for their UT-A2 inhibitory activity through in vitro tests. Among them, Compound E822-1968 was confirmed to inhibit UT-A2 activity. **c** Heatmap visualization of the IC_50_ values for 30 candidate compounds targeting UT-A2 mediated urea transport. Notably, compound E822-1968 exhibited inhibitory efficacy. **d** The molecular formula of compound E822-1968 which shows inhibitory potency toward UT-A2 with the IC_50_ = 0.44 ± 0.03 μM.

In this study, we identify a selective inhibitor with relatively high efficacy against the UT-A2 protein through a combination of progressive hotspot pocket-based virtual screening, in vitro testing, structural mechanical analysis and selective function validation (Fig. 1a). Our work not only offers a selective pharmacological tool to elucidate the unique physiological functions of UT-A2, but also provides a method for efficiently developing subtype-selective UT inhibitors with potent inhibitory activity as next-generation diuretics.

## Results

### Hotspot pocket-based virtual screening for UT-A2 inhibitors

Our previous work on UT structures revealed that the UT protein has various antagonist binding pockets, which have been proven to bind different types of UT inhibitor molecules, thus playing essential roles in blocking urea transport^7^. We define these pockets as hotspot pockets for UT inhibitors. To better define the specific range of different hotspot pockets, we used DoGSiteScorer^20^ to predict the possible ligand binding pockets on UT-A2 (Supplementary Fig. 1a). Subsequently, to avoid the impact of reduced bioavailability due to the transmembrane movement of small-molecule inhibitors, we excluded the binding pockets located in the intracellular segment of the UT channel. Finally, we identified the cavity surrounded by the extracellular lateral α-helices 3a, 2b-4b, Pb and loop LECL on the UT-A2 structure (Supplementary Fig. 1b) as the hotspot pocket regions for virtual screening. This pocket mainly includes the extracellular urea binding pocket (EUBP), the extracellular blocker binding pocket (EBBP), and the “SCG” pocket (Supplementary Fig. 1c and Supplementary Table 1).

**Fig. 2:**
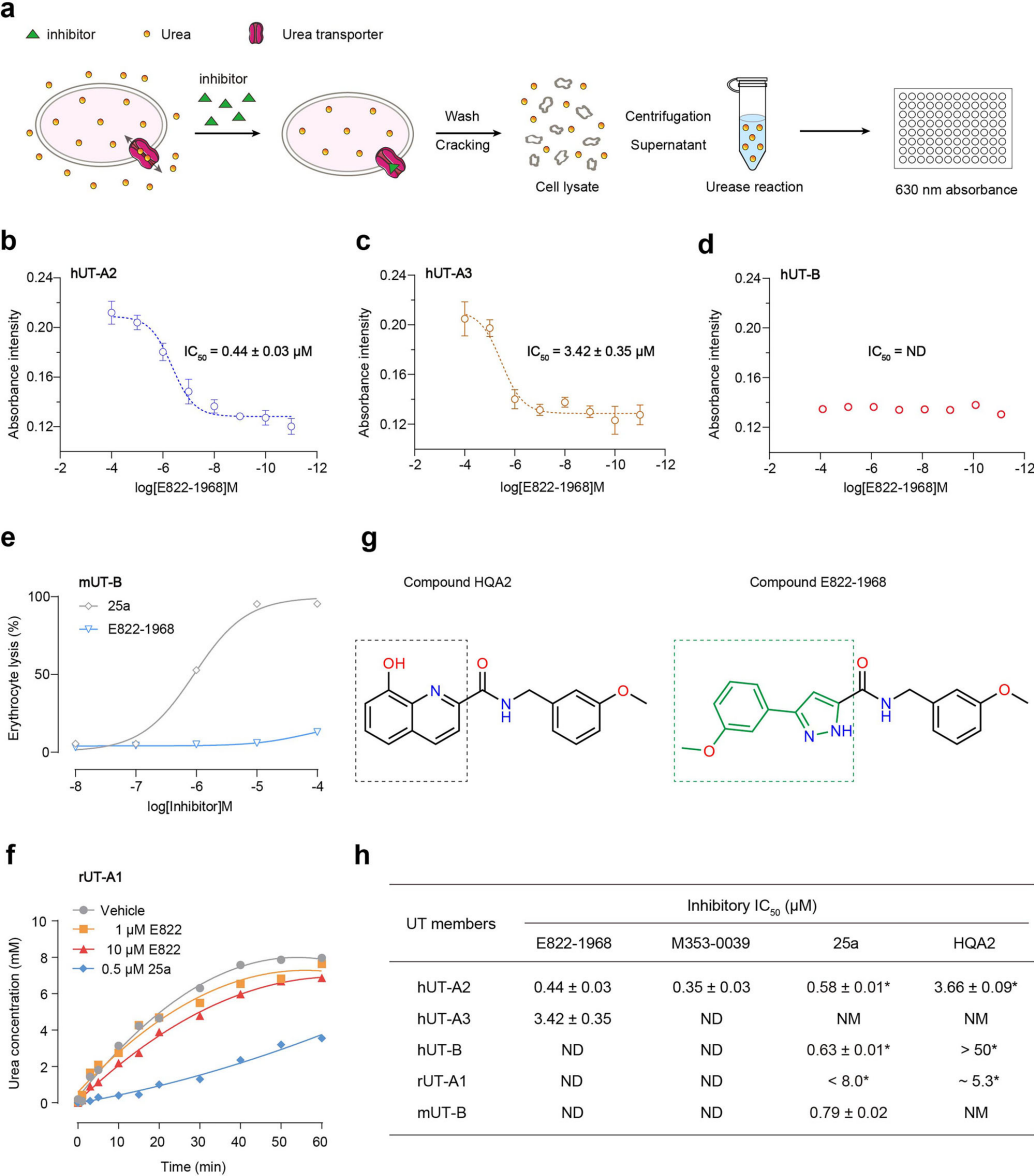
Validation of the inhibitory efficacy of E822-1968 targeting different UTs. **a** Schematic illustrating the urease reaction assay to evaluate the inhibitory efficacy of candidate compounds targeting UT-A2. **b**–**d** The inhibitory effects of E822-1968 to human UT-A2 (b), UT-A3 (**c**) and UT-B (**d**) validated by the urease reaction assay. E822-1968 showed inhibitory potency toward UT-A2 and UT-A3 with the IC_50_ = 0.44 ± 0.03 μM and 3.42 ± 0.35 μM, respectively. Each data was derived from three independent experiments (*n* = 3) to determine the IC_50_ values. Data are shown as the means ± SEM. ND not detected. **e** E822-1968 showed no dose-dependent inhibitory effects on mouse UT-B (mUT-B) in erythrocyte lysis assay, as compared to the positive control (compound 25a). **f** E822-1968 exhibited no significant inhibitory effects on rat UT-A1 stably expressed in MDCK cells using transwell assay, as compared to the positive control (compound 25a). **g** Structural formula comparison between E822-1968 and the previously reported UT-A inhibitor HQA2. The difference between the two compounds lies in the replacement of the hydroxyquinoline moiety in HQA2 with a pyrazole-anisole moiety of E822-1968. **h** The IC_50_ table of the UT inhibitors against different UT members. ND not detected; NM no measured. *, data from our previously work reported by Huang, et al.

We subsequently conducted forward virtual screening via molecular docking on the defined hotspot pocket by using a custom-designed compound database including one million compounds. We chose the top 1000 small molecules with the highest docking scores in the databases and performed clustering analysis on the basis of scaffold identification. Notably, the residues identified in the “SCG” pocket were considered pivotal for inhibitor selectivity, because the residues lining this pocket are non-conserved across different UT subtypes. To increase the selectivity of compounds targeting UT-A2, we specifically excluded small molecules that were likely to interact with residues in the “SCG” pocket during the selection process. Ultimately, we identified 30 compounds that showed promise in terms of both biological activity and selectivity (Fig. 1b). By conducting a urease reaction assay in UT-A2-overexpressing HEK293F cells, we found that one particularly promising compound, E822-1968, could effectively block urea transport via UT-A2, with an IC_50_ value of 0.44 ± 0.03 μM (Figs. 1c, d, 2a, b and Supplementary Fig. 1d–k; Supplementary Table 2–4).

### E822-1968 is a candidate UT-A selective inhibitor

We then conducted a further urease reaction assay to evaluate the impact of compound E822-1968 on UT-A3 and UT-B (Fig. 2a). Our results revealed that E822-1968 was unable to efficiently inhibit the rapid transmembrane transport of urea in UT-B-overexpressing HEK293F cells, but shown weak inhibitory effect on UT-A3 with an IC_50_ value of 3.42 ± 0.35 μM (Fig. 2c, d). We also assessed the efficacy of E822-1968 to UT-B by performing an erythrocyte lysis assay. Compared with the positive control compound 25a, E822-1968 failed to block the urea transport mediated by UT-B and UT-A1 (Fig. 2e, f). Structural formula comparison revealed that E822-1968 shares partial structural similarity with the previously reported UT-A inhibitor HQA2 (Supplementary Table 2)^7^. The difference between the two compounds lies in the replacement of the hydroxyquinoline moiety in HQA2 with a pyrazole-anisole moiety of E822-1968 (Fig. 2g). Interestingly, E822-1968 demonstrated 8-fold higher inhibitory potency against UT-A2 compared to HQA2. Collectively, these findings demonstrated that E822-1968 inhibits the UT-A2 protein subtype and exhibits weak inhibitory activity on UT-A3, but has no efficacy against UT-B (Fig. 2h).

### The binding pocket of E822-1968 in UT-A2

**Fig. 3:**
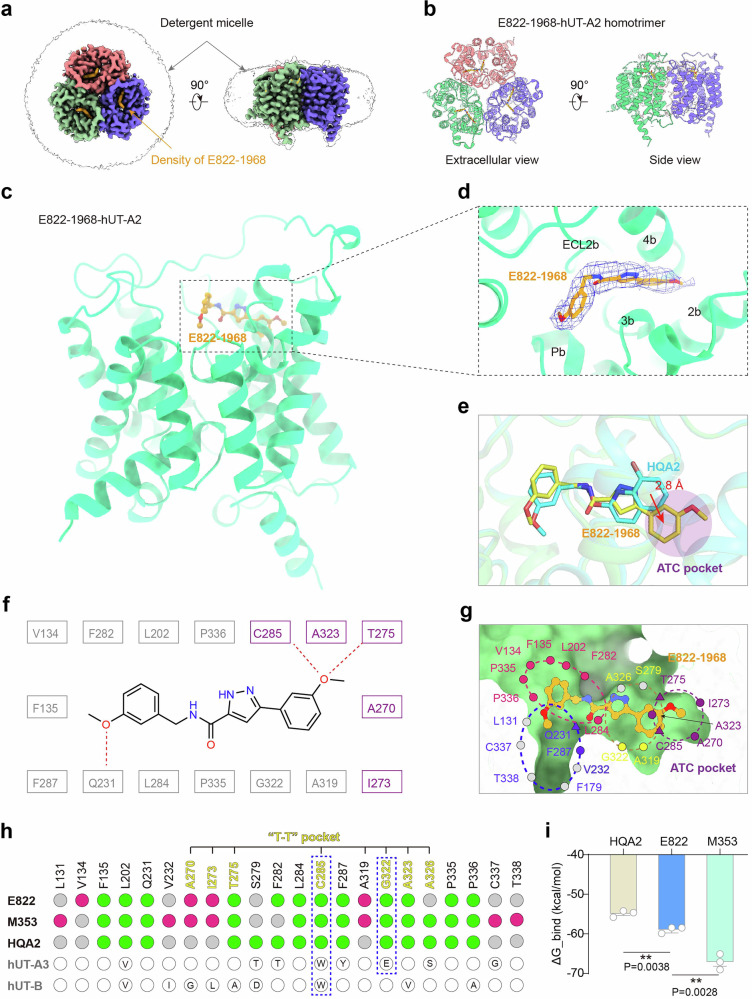
Structural characterizations of E822-1968-UT-A2 complex. **a** Cryo-EM densities of E822-1968-UT-A2 homotrimer from the extracellular view (left) and the side view (right). The EM densities of UT are surrounded by the detergent micelle with densities of E822-1968 colored by orange. **b** Structural representation of E822-1968-UT-A2 homotrimer from the extracellular view (left) and the side view (right). **c** The E822-1968 bound in the extracellular side of UT-A2. **d** The cryo-EM density (blue mesh) of E822-1968 is located in the cavity surrounded by helices 2b-4b, Pb and loop ECL2b. **e** Position comparison between E822-1968 and HQA2. The benzene ring in the pyrazole-anisole moiety of E822-1968 exhibited a shift with 2.8 Å into the extended ATC pocket. HQA2 is drawn in turquoise, and E822 is rendered in gold. **f** A diagram shows the residues in the E822-1968 binding pocket of UT-A2. The hydrogen bonds and polar interactions are shown as red dashed lines. The residues in the ATC pocket are colored by purple. **g** Cutaway view of the E822-1968 binding pockets. E822-1968 occupied the EBBP, SCG pocket and ATC pocket, represented by magentas, yellow and purple dashed line circles. Residues forming polar interactions or hydrogen bonds with E822-1968 are depicted using triangles. Residues forming hydrophobic interactions are depicted as solid round circles, while residues that do not interact with E822-1968 are shown as gray circles. **h** Barcode comparisons of the residues in the binding pocket of different UTs. The residues interacting with E822-1968, M353-0039 and HQA2 are colored green, and residues interacting with E822-1968 or M353-0039 are colored magentas. The residues with no interactions are shown as gray dots. The residue sequences of UT-A2 are displayed on the top line, with the residues in “T-T” pocket colored yellow. The letters inside the black circles represent the non-conserved residues of UT-A3 or UT-B. The blue dashed line boxes indicate the crucial residues for the selectivity of M353-0039. **i** Comparison of the best estimated ΔG_binding energies of HQA2, E822-1968 and M353-0039 to UT-A2. Statistical differences were determined by the two-sided unpaired Student’s t-test. The data are shown as the means ± SEM, *n* = 3.

To further understand the molecular mechanism by which compound E822-1968 acts on UT-A2, we solved the high-resolution structure of E822-1968-bound human UT-A2 using single-particle cryo-electron microscopy, with an overall resolution of 2.9 angstroms (Supplementary Fig. 2a–h and Supplementary Table 5). The structure showed that the E822-1968-bound UT-A2 complex is a homotrimer complex surrounded by detergents (Fig. 3a, b). Compared with the cryo-EM map of UT-A2 in the resting state (PDB code: 8XD9), additional density was found on the extracellular side of the channel in the E822-1968-UT-A2 structure, which is unambiguously defined to the compound E822-1968 in the refined structural model (Fig. 3a, c, d). The binding pocket of E822-1968 is located in the cavity surrounded by the extracellular α-helices 2b-4b, Pb and loop ECL2b (Fig. 3d), which occupies part of the hotspot pocket relied upon by virtual screening (Supplementary Fig. 1c). Compared to the binding pose of HQA2, the pyrazole-anisole moiety of E822-1968 exhibited a significant positional shift, with its benzene ring moving ~2.8 Å into a subpocket contiguous with but outside of the EBBP and SCG pockets (Fig. 3e, g and Supplementary Table 1). This subpocket, located within the space enclosed by residues A270, I273, T275, C285 and A323, was named the “ATC pocket” (ATCP) (Fig. 3f, g, Supplementary Fig. 3a, b and Supplementary Table 1). Sixteen residues of UT-A2 formed direct interactions with E822-1968, two more than the number interacting with HQA2 (Fig. 3h). Specifically, residues Q231 and T275/C285 engaged in hydrogen bonding and polar interactions with E822-1968, respectively (Fig. 3f and Supplementary Table 6). Moreover, binding free energy calculations revealed that the binding energy of E822-1968 for UT-A2 was significantly greater than that of HQA2 (Fig. 3i). These findings elucidate the molecular structural basis for the superior inhibitory efficacy of E822-1968 over HQA2 against UT-A2.

### Redocking based on the refreshed hotspot pocket

**Fig. 4:**
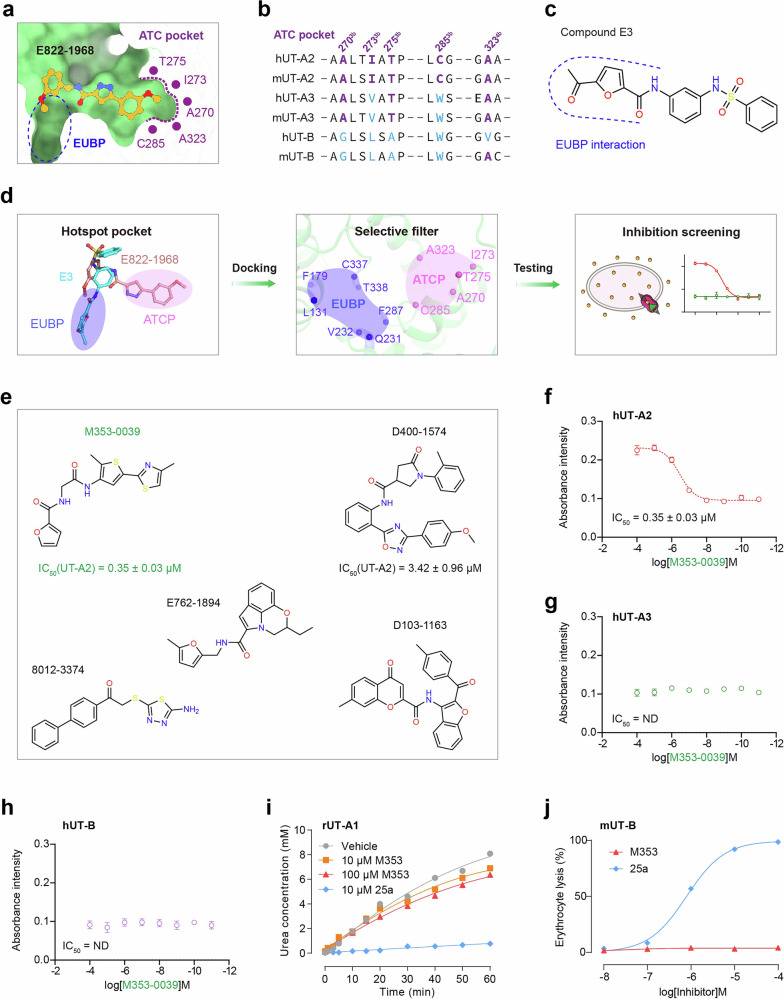
Progressive rescreening of selective inhibitors targeting UT-A2. **a** Cutaway view of the ATC pocket and the EUBP (extracellular urea binding pocket). **b** Sequence alignment of the structurally equivalent residues in the ATC pocket of hUT-A2 compared with hUT-A3, hUT-B, mUT-A2, mUT-A3 and mUT-B. Key residues of ATC pocket in hUT-A2 are shown in purple font on the top line. Sequences with the same amino acids as the ATC pocket of hUT-A2 are represented in purple, while those with non-conserved amino acids are represented in light blue. The letters ‘h’, ‘m’ before the UT indicates the UTs from human and mouse, respectively. **c** Diagram of compound E3 engaging with EUBP with the interfaces represented by blue dashed lines. **d** The strategy of progressive rescreening UT-A2 selective inhibitors. The hotspot pocket is refreshed in the regions mainly containing ATCP and EUBP of UT-A2, and the selective filter is based on the interactions between candidate compounds and the key residues of both EUBP and ATCP. **e** Structural formulas of the top five candidate compounds for UT-A2 selective inhibitors in progressive rescreening. The compounds M353-0039 and D400-1574 showed inhibitory potency to UT-A2 with IC_50_ value of 0.35 ± 0.03 μM and 3.42 ± 0.96 μM. **f**–**h** The inhibitory effects of M353-0039 to human UT-A2 (f), UT-A3 (g) and UT-B (h) validated by the urease reaction assay. M353-0039 showed inhibitory potency toward UT-A2 with the IC_50_ = 0.35 ± 0.03 μM. Each data was derived from three independent experiments (*n *= 3) to determine the IC_50_ values. Data are shown as the means ± SEM. ND not detected. **i** M353-0039 exhibited no significant inhibitory effects on rat UT-A1 stably expressed in MDCK cells using Transwell assay, as compared to the positive control (compound 25a). **j** M353-0039 showed no dose-dependent inhibitory effects on mouse UT-B in erythrocyte lysis assay (*n* = 3), as compared to the positive control (compound 25a).

We systematically evaluated the conservation patterns of amino acid sequence of the ATCP (A270/I273/T275/C285/A323) among distinct UT protein subtypes (Fig. 4a, b and Supplementary Table 1). Comparative sequence analysis demonstrated that the ATCP exhibits marked non-conservation in UT-B, with residues I273 and C285 showing similar divergence in UT-A3 (Fig. 4b). These results imply that the ATCP may serve as a structural determinant for the development of subtype-selective UT-A2 inhibitors. The cutaway view of the binding pocket revealed that E822-1968 does not penetrate deeply into the EUBP (extracellular urea binding pocket) (Fig. 4a and Supplementary Table 1), contrasting with our recent findings on the high-affinity UT-A inhibitor E3 (Supplementary Table 2)^21^. Notably, E3 achieves enhanced binding affinity through engagement with the EUBP (Fig. 4c and Supplementary Fig. 3c; Supplementary Table 1). Consequently, we integrated spatial constraints from both the ATCP and EUBP regions to define a refreshed hotspot pocket for the next round of molecular docking (Supplementary Fig. 3d). Furthermore, we implemented a filtering strategy to screen molecules based on their interactions with key residues within these two pockets (Fig. 4d). Finally, we selected five small molecules with the highest potential for selective UT-A2 interaction and conducted in vitro assays to evaluate their inhibitory activity (Fig. 4e).

### M353-0039 is a UT-A2 selective inhibitor

The urease reaction assay showed that compounds M353-0039 (Supplementary Table 4) and D400-1574 exhibited inhibitory activity against UT-A2, with IC_50_ values of 0.35 ± 0.03 μM and 3.42 ± 0.96 μM, respectively (Fig. 4e, f and Supplementary Fig. 4a; Supplementary Table 2). Further investigations revealed that M353-0039 did not show inhibitory activity against UT-A3 and UT-B, suggesting a lack of functional activity towards these two transporters (Fig. 4g, h). To further validate these findings, we employed the transwell assay system using MDCK cells which stably express UT-A1. At concentrations of 10 μM and 100 μM, M353-0039 exhibited no significant inhibition of UT-A1-mediated urea transport compared to the positive control compound 25a (Fig. 4i). Similarly, in erythrocyte lysis assays, M353-0039 failed to block UT-B-facilitated urea transport (Fig. 4j), confirming its subtype selectivity. Collectively, these results demonstrate that M353-0039 exhibits potent and selective inhibition of UT-A2, while showing no detective activity against UT-A3 or UT-B.

### Structures of the M353-0039-UT-A2 complex

**Fig. 5:**
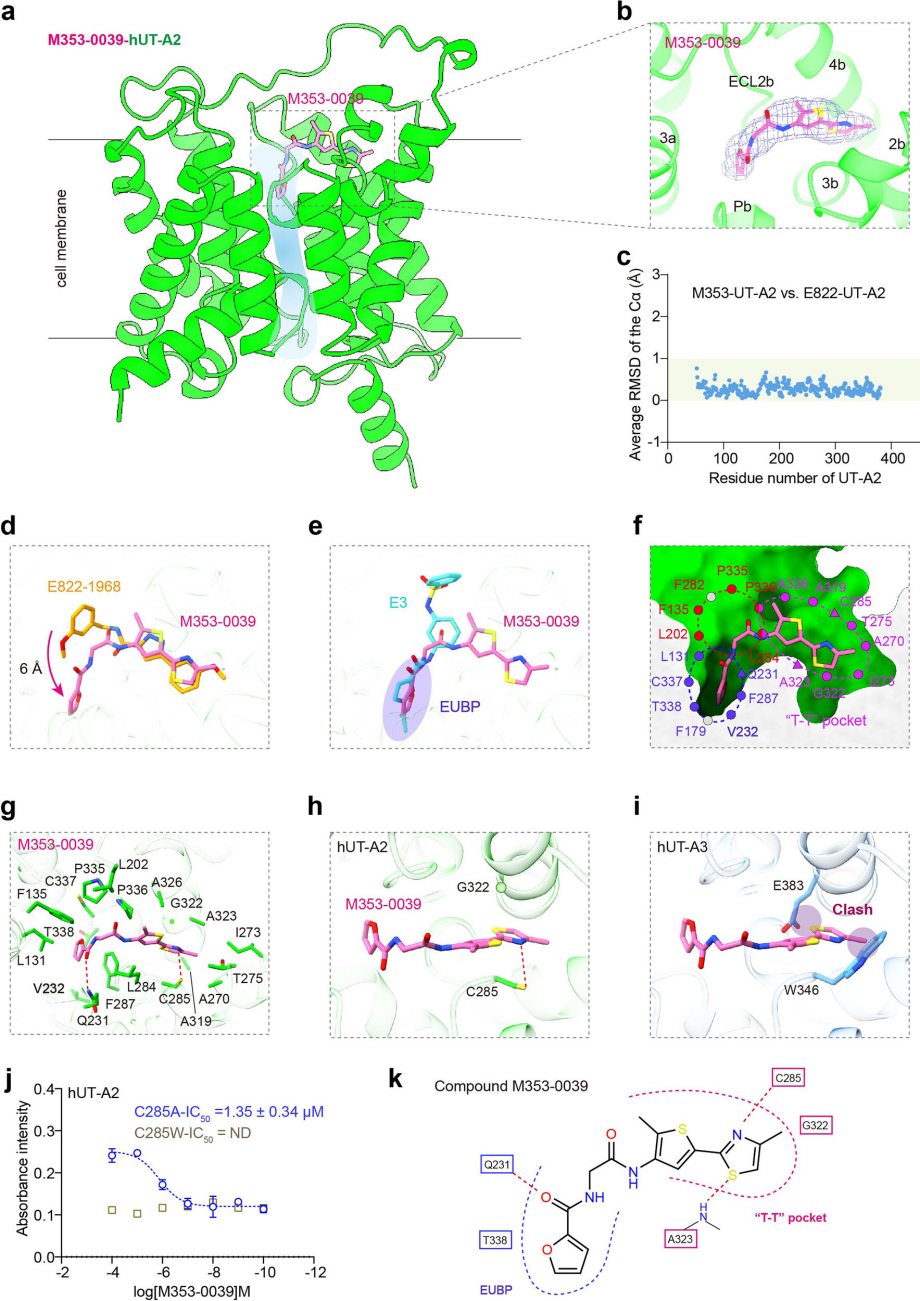
Structural basis of M353-0039’s selective interaction with UT-A2. **a**,**b** Structural representation of M353-0039-UT-A2 complex (**a**). The cryo-EM density (blue mesh) of M353-0039 is located in the cavity surrounded by helices 3a, 2b-4b, Pb and loop ECL2b (**b**). **c** Plot of the distance root mean square deviations (RMSDs) of Cα deviations between M353-0039-UT-A2 and E822-1968-UT-A2 structures. The vertical and horizontal axes indicate the RMSDs and the residues sequence of the UT-A2, respectively. The blue area represents Cα deviations less than 1.0 Å. **d**,** e** Binding position comparison of M353-0039, E822-1968 and E3 in UT-A2. The furan ring of M353-0039 is 6 Å deeper into the urea transport channel (**d**) and engages with the EUBP same as compound E3 (**e**). **f** Cutaway views of M353-0039 binding pocket. The furan moiety of M353-0039 occupies the EUBP (blue dashed circles). The EBBP is demarcated by red dashed circles, while the thiazole-thiophene moiety engages the “T-T” subpocket (magenta dashed circles). Residues forming polar interactions or hydrogen bonds with M353-0039 are depicted using triangles. Residues forming hydrophobic interactions are depicted as solid dots. The residues that have no interaction with M353-0039 are shown as gray dots. **g** The key residues in M353-0039 binding pocket are shown. The hydrogen bonds are shown as red dash line. **h**, **i**. Comparison of different residues in the M353-0039 binding pocket between UT-A2 (**h**) and UT-A3 (**i**). The C285 of UT-A2 is replaced by W346 of UT-A3 and the G322 of UT-A2 is replaced by E383 of UT-A3, thus cause steric hindrance to M353-0039. The hydrogen bond between C285 and M353-0039 is shown as red dash line. **j** Inhibitory effects of M353-0039 on the UT-A mutants C285A and C285W. Data are shown as the means ± SEMs (*n* = 3) to determine the IC_50_ values. ND, not detected. **k** The diagram of M353-0039 engaging with the EUBP and “T-T” pocket with the interfaces represented by blue and violet dashed lines, respectively. Key residues with EUBP and “T-T” pocket are shown in blue and violet rectangles. The hydrogen bonds and polar interactions are shown as red dash line.

We subsequently determined the structure of UT-A2 bound with M353-0039 at an overall resolution of 2.7 Å (Fig. 5a, Supplementary Fig. 4b–j and Supplementary Table 5) with a clear density of inhibitor M353-0039 which is located in a cavity surrounded by the extracellular α-helices 3a, 2b-4b, Pb and loop ECL2b (Fig. 5b). The overall structure of M353-0039-bound UT-A2 is almost identical to the structure of E822-1968-bound UT-A2, and the root mean square deviation (RMSD) of the Cα atoms in the structure of M353-0039-UT-A2 complex is less than 1.0 angstroms, with an average deviation of only 0.27 angstroms compared with the E822-1968-UT-A2 structure (Fig. 5c). These results suggest that similar structural conformations of UT-A2 are induced by these two different inhibitors. However, there are differences in the binding configurations of M353-0039 and E822-1968. Compared with E822-1968 and HQA2, the furan ring of M353-0039 is ~6 angstroms deeper into the urea transport channel (Fig. 5d and Supplementary Fig. 5a) and engages with the EUBP same as compound E3, which plays a role in blocking urea transport (Fig. 5e), whereas the thiophene-thiazole ring shows no significant positional change (Fig. 5d). These results indicate that M353-0039 occupies a deeper binding pocket than E822-1968 in UT-A2. The cutaway view reveals that, in addition to interacting with the EUBP and EBBP1 subpockets (Supplementary Table 1), the thiophene-thiazole ring of M353-0039 also forms a new interaction subpocket in UT-A2, named “T-T” pocket because of its interactions with the thiophene-thiazole ring (Fig. 5f and Supplementary Table 1). These differences in binding configurations between M353-0039 and E822-1968 reflect their different inhibitory effects on UT-A2.

### Structural basis for the increased potency of M353-0039

Compared with HQA2 (IC_50_ = 3.66 ± 0.09 μM), the IC_50_ value for M353-0039-bound UT-A2 for inhibiting urea transport increased ~10-fold to 0.35 ± 0.03 μM (Figs. 2h, 4e, f). Interactional barcode comparison revealed that M353-0039 interacted with 19 residues of UT-A2, which was significantly more than the 14 residues for HQA2 (Figs. 3h, 5g, Supplementary Fig. 5b and Supplementary Table 7). In the “T-T” pocket, M353-0039 formed new hydrogen bond and polar interaction with the thiol group of C285 and the carbonyl group of A323 respectively (Fig. 5f, g). M353-0039 formed hydrophobic interactions with A270, I273 and A319. Furthermore, in addition to maintaining hydrogen bond with Q231, M353-0039 also formed additional hydrophobic interactions or van der Waals forces with L131, V232, C337, and T338 in EUBP (Fig. 5f, g). Ligand binding free energy calculations indicated that the binding free energy of M353-0039 with UT-A2 was greater than that of HQA2 and E822-1968 with UT-A2 (Fig. 3i). Collectively, these data provide the structural basis for the superior inhibitory potency of M353-0039 towards UT-A2.

### Structural mechanism underlying the selectivity of M353-0039

Next, we analysed the structural mechanism underlying the selectivity of M353-0039 for UT-A2. Sequence comparison of the binding pocket revealed that the leucine at position 202 (L202) in the L-P subpocket and the cysteine at position 285 (C285) in the T-T subpocket were not conserved in UT-A2, UT-A3 or UT-B (Fig. 3h). Although the L-P pocket has been reported to potentially affect the selectivity of inhibitors, it contributed less to the selectivity of M353-0039 because L202 only formed hydrophobic interaction with M353-0039 (Fig. 5g and Supplementary Table 7). The smaller side chain of C285 on UT-A2 not only provided ample space for M353-0039 to enter the ligand pocket but also formed a hydrogen bond with the nitrogen atom of the thiazole ring in M353-0039 (Fig. 5g, h). In contrast, on UT-A3 and UT-B^22^, the residues spatially equivalent to C285 of UT-A2 are W346 and W286, respectively. These residues possess bulkier side chains that hinder access of M353‑0039 to this binding pocket, although the furan ring of M353‑0039 inserts into the EUBP in the same manner as the 2‑thienyl group of UTBinh‑14, as observed in the UTBinh‑14-UT‑B complex (Fig. 5i and Supplementary Fig. 5c, d). In addition, we found that residue E383 in UT-A3, whose equivalent residue is G322 in the T-T pocket of UT-A2 (Fig. 3h), also exerted steric hindrance against the thiazole ring of M353-0039 (Fig. 5i). These larger side chains clashed with the thiazole ring of M353-0039 as it attempts to enter the pocket, thereby preventing the effective binding of M353-0039 to UT-A3 and UT-B. Moreover, the C285A mutant of UT-A2 significantly reduced the inhibitory potency of M353-0039, whereas the C285W mutant completely abolished the interaction between M353-0039 and UT-A2 (Fig. 5j). Therefore, residues C285 and G322 on the T-T pocket played a crucial role in determining the inhibitor selectivity among UT-A2 and UT-A3/UT-B (Fig. 5k). These results suggested that the T-T pocket, which is located completely independent of the urea transport channel, can serve as an extrachannel subpocket on the extracellular face for designing selective inhibitors towards different UT subtypes.

### M353-0039 selectively inhibits UT-A2 in vivo

**Fig. 6:**
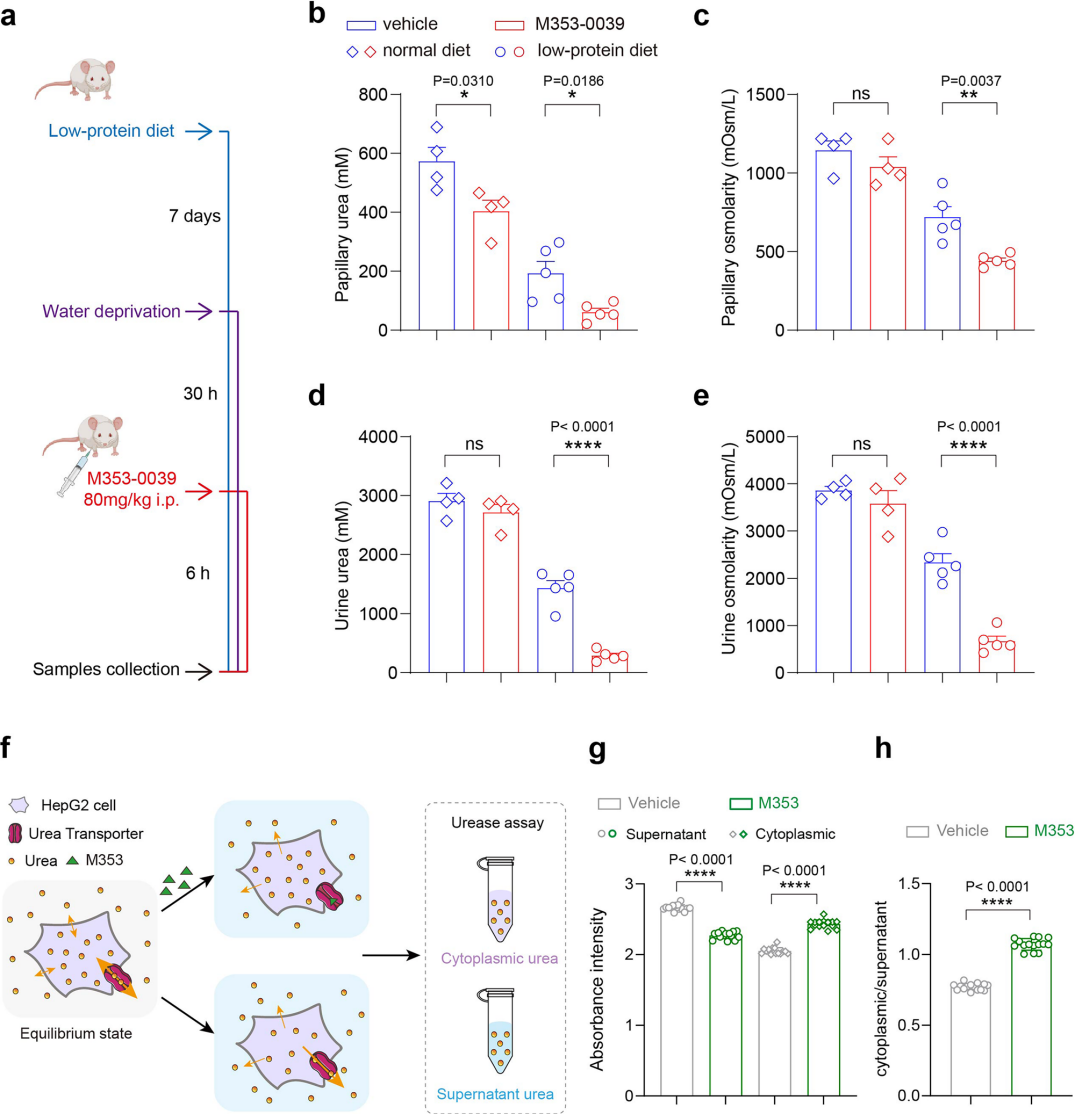
The selective effects of M353-0039 on UT-A2 in vivo and at the cellular level. **a** Workflow for assessing the selective action of M353-0039 on UT-A2 in mice. **b**,**c** The urea concentration (**b**) and osmolarity (**c**) in the kidney medulla were measured in mice under a low-protein (*n* = 4) /normal diet (*n* = 5), with or without intraperitoneal injection of M353-0039. The sample size (*n*) represents the biological replicates. The urea concentration and osmolarity of the papilla were measured in the supernatant of renal medulla homogenate supplied with 10 volume double-distilled water. *P < 0.05; **P < 0.01; ns, no significant; statistical differences were determined by the two-sided unpaired Student’s t-test. Data are shown as the means ± SEM. **d**,** e** The urea concentration (**b**) and osmolarity (**c**) in the urine were measured in mice under a low-protein diet (*n* = 4)/normal diet (*n* = 5), with or without intraperitoneal injection of M353-0039. The sample size (n) represents the biological replicates. ***P < 0.001; ns, no significant; statistical differences were determined by the two-sided unpaired Student’s t-test. Data are shown as the means ± SEM. **f** The experimental procedure for detecting the inhibitory effect of M353-0039 on HepG2 cells. **g**,** h** Effect of M353-0039 on urea transport in HepG2 cells. The data showed the urea concentration in the supernatant and cytoplasmic compartments (**g**), as well as in a cytoplasmic/supernatant format (**h**). The M353-0039 could inhibit urea efflux in HepG2 cells. ***P < 0.001, ****P < 0.0001; statistical differences were determined by the two-sided unpaired Student’s t-test. Data were derived from fifteen independent experiments (*n* = 15) and shown as the means ± SEM.

UT-A2 is the major urea transporter of the thin descending limb of the loop of Henle in nephrons. Furthermore, this transporter plays a critical role in maintaining hyperosmotic urea concentrations in the inner medulla under conditions of limited renal urea supply. UT-A2 knockout mice that are fed a low-protein diet with water deprovision have been shown to exhibit a reduction in maximal urine osmolality as well as a decrease in the urea concentration within the inner medulla when compared with wild-type mice^23^. Therefore, we administered an intraperitoneal injection of 80 mg/kg M353-0039 to validate the in vivo selective potency in wild-type mice with a low-protein diet and water deprivation to simulate the effects observed in UT-A2 knockout mice (Fig. 6a). Six h after the injection, we collected urine and kidney medulla tissue samples from the mice and analysed the urea concentration and osmolarity (Fig. 6a). The results revealed that the urea concentration and osmolarity in the kidney medulla of the wild-type mice treated with M353-0039 were lower than those observed in the control group (Fig. 6b, c), which was consistent with the phenomenon observed in the UT-A2 knockout mice. These findings suggested that M353-0039 exerts selective inhibitory effects on UT-A2 within the mouse kidney medulla. As for urine evaluation, we found that low protein diet caused increased urine volume before water deprivation (Supplementary Fig. 5e), but the urine volume was rare after water deprivation for 36 h. Therefore, the urine was collected by bladder pressure for detection. Interestingly, there was also a significant reduction in the urea concentration and osmolarity in the mouse urine (Fig. 6d, e). We hypothesize that this phenomenon may have resulted from the reduced urea concentration and osmolarity in the medulla. As urine flows through the inner medullary collecting duct, a portion of the urea molecules are transported from the duct lumen into the medulla via UT-A1 and UT-A3. Furthermore, we conducted parallel experiments on wild-type mice fed a normal diet and deprived of water. The results revealed a minor decrease in the urea concentration in the kidney medulla (Fig. 6b), whereas no significant changes were observed in the urine (Fig. 6c–e), which are similar to those observed in UT-A2 knockout mice fed a normal diet^23^. In summary, these observations demonstrated that M353-0039 selectively blocks urea transport mediated by UT-A2 in mice, thus indicating that this compound could serve as a tool for investigating the special physiological role of the UT-A2 subtype.

### M353-0039 blocks urea transport in hepatic cell

The urea cycle in the liver is the main metabolic pathway used to convert excess nitrogen into disposable urea^24^. Phloretin-inhibitable urea efflux associated with the UT-A urea transporter family has been detected in cultured hepatocytes. Western blot analysis of HepG2 cells and the liver tissues from rats, mice, chimpanzees with a UT-A antibody revealed two protein bands at 49 kDa and 36 kDa, which were localized to the membrane fraction and cytoplasm, respectively^25^. However, since the UT-A antibody used recognizes UT-A1, UT-A2 and UT-A4, it remains unclear which UT-A subtype is the primary isoform that mediates urea transport in liver cells. We incubated HepG2 cells cultured with 300 mM urea solution in the presence or absence of the inhibitor M353-0039 for 20 min. The cells and supernatant were then separated by centrifugation. The intracellular urea was released into the solution by lysing the cells with triple-distilled water. The urea concentrations in the supernatant and cytoplasmic lysate were measured separately using a BUN detection kit (Fig. 6f). The results revealed that the urea concentration in the cytoplasmic lysate was significantly higher in the presence of M353-0039 than in the control group, whereas the opposite trend was observed in the supernatant (Fig. 6g-h). These findings indicated that M353-0039 effectively blocks urea transport in HepG2 cells, suggesting that UT-A2 participates the transmembrane transportation of urea in liver cells. Therefore, as a selective inhibitor of UT-A2, M353-0039 can serve as a promising tool for investigating the specific physiological functions of UT-A2.

## Discussion

In this study, we identified a selective inhibitor of UT-A2 with relatively high activity, by employing a research framework that encompasses progressive hotspot pocket-based virtual screening, in vitro testing, structural analysis and functional validation. Furthermore, we elucidated the structural basis involved in the selective mechanisms for different UT subtypes, thus offered direct scientific methodologies for designing selective UT-A inhibitors, which proposes a more efficient method for developing selective UT inhibitors compared to conventional scaffold-hopping approaches alone.

Through a two-round progressive hotspot pocket-based virtual screening and in vitro testing, we determined that the compound M353-0039 effectively blocked urea transport via UT-A2, with an IC_50_ value of 0.35 ± 0.03 μM, which is 10 times greater than that of previously reported inhibitors, HQA2. In the first round of virtual screening, we utilized a relatively broad definition for the hotspot pocket. While this approach facilitated the identification of diverse binding modes, it also reduced the enrichment efficiency for functionally blocking inhibitors, because the effective inhibitions of urea transports depend not only on binding affinity, but also requires the inhibitor to physically occlude the urea permeation pathway. Consequently, from 30 candidate compounds tested, only one demonstrated measurable inhibitory activity. Based on this experience, the second-round screening focused on a refined hotspot pocket targeting the ATCP/EUBP sub-pocket, which improved the hit rate and ultimately led to the successful identification of a inhibitor, M353-0039, with enhanced selectivity. By coincidence, in the first-round screening, we discovered an inhibitor, E822‑1968, that exhibits partial structural similarity to HQA2 in their molecular scaffolds, notably a shared methoxyphenyl group (Fig. 2g). This finding implies that conventional structure‑based drug design (SBDD) and structure–activity relationship (SAR) approaches may guide optimization of HQA2 toward E822-1968-type molecules. Nevertheless, such a strategy is unlikely to afford M353-0039, owing to its completely distinct molecular framework.

Notably, M353-0039 is a specific inhibitor that targets UT-A2 without inhibitory activity on UT-A1/UT-A3 or UT-B. As the privoiusly unreported UT‑A2 selective inhibitor, we unexpectedly observed that M353‑0039 lacks appreciable activity against UT‑A1, although UT-A2 is essentially identical with one half of the UT-A1 molecule. UT-A1 shares identical amino acid sequence composition with the combined UT-A2 and UT-A3 domains, but the quaternary structure of UT-A1 remains structurally uncharacterized. Gamma Chi et al. attempting to resolve UT-A1’s structure exclusively identified a trimeric assembly derived from the N-terminal UT-A3 segment, with no discernible electron density corresponding to the C-terminal UT-A2 portion^22^. Concordantly, our prior work demonstrated that while UT-A2 and UT-A3 exhibit robust membrane localization upon heterologous overexpression, UT-A1 shows negligible membrane integration under identical conditions. These findings suggest that the physiological structure of UT-A on cell membrane is likely not a simple modular assembly of its constituent UT-A2 and UT-A3 subunits, which may lead to the functional phenomenon that inhibitors selective for UT-A2, like compound M353-0039, exhibit little efficacy against UT-A1.

We also solved the high-resolution structure of M353-0039-bound UT-A2 to explore the detailed structural mechanism underlying the ligand interactions and selectivity. This structure revealed a different ligand binding pose from those of the other inhibitors, HQA2 and E822-1968. M353-0039 occupied a deeper binding pocket and formed more interactions with UT-A2 residues, especially the hydrogen bond with residue C285, thus contributing to the superior inhibition potency of M353-0039 compared with HQA2. Furthermore, the selectivity of M353-0039 for UT-A2 was attributed to the nonconserved residue C285 and G322 in a unique T-T subpocket on the extracellular side of UT-A2. Although the action types (competitive or non-competitive) of M353-0039 have not yet been established, these observations revealed crucial regions and key residues, which provided useful information for designing selective inhibitors targeting various UT subtypes as well as guidance for conducting efficient structure-based modifications of UT inhibitors.

As the unreported UT-A2 selective inhibitor, we validated the functional activity of M353-0039 in selectively inhibiting UT-A2 in both mouse and cellular models. Shinichi Uchida et al.^23^ had used gene-targeting technology to selectively disrupt UT-A2 expression without affecting the expression of other renal urea transporters. Unexpectedly, compared with UT-A1/A3-knockout and UT-B-knockout mice, UT-A2-knockout mice displayed only a mild defect in urine-concentrating ability, and this phenotype was observed only under stress conditions (low-protein diet combined with dehydration). These results indicate that UT-A2 function is “protein-diet dependent”. They suggested that the functional importance of UT-A2 may be more pronounced in wild animals with inadequate daily protein intake than in laboratory animals, whereas the standard laboratory animal housing conditions might mask UT-A2’s physiological significance. Consequently, under normal dietary conditions, the effect of UT-A2 inhibitor is not evident. Under low-protein-plus-dehydration conditions, however, the critical role of UT-A2 in maintaining a high urea concentration gradient in the inner medulla becomes apparent, thereby helping us to evaluate the selective inhibitory effect of M353-0039 in vivo. In order to verify the in vivo activity of M353-0039, we applied the same experimental conditions as those employed by Uchida, S. et al. to treat the mice, because the residues of the binding pocket of M353-0039 in human and mouse are completely identical (Supplementary Fig. 5f). After water deprivation, urine volume fell sharply and viscosity rose, so metabolic cages failed to collect accurate volumes. We therefore collected bladder urine as Uchida, S.’s method to measure urea concentration and osmolality. After the intraperitoneal injection of M353-0039 into mice that were fed a low-protein diet, we found that M353-0039 significantly reduced the urea concentration and osmolarity in the renal medulla. In contrast, under normal dietary conditions, M353-0039 had a minimal effect on the urea concentration in the renal medulla and did not affect the urea concentration or osmolarity in the urine. These results are similar to the effects observed after the knockout of UT-A2 in mice, indicating that M353-0039 can effectively inhibit UT-A2-mediated urea transport in vivo. Furthermore, Klein JD et al. have suggested the possible presence of a UT-A2 isoform protein in hepatocytes, but this has not been conclusively confirmed^25^. Here, we performed cell-based experiments and found that hepatocytes do indeed express UT-A2 protein as M353-0039 is an UT-A2 selective inhibitor, which was further confirmed by qPCR (Supplementary Fig. 5g, h). However, as to whether hepatocytes express other types of UT protein isoforms, and to what extent UT-A2 contributes to hepatic urea transport, future studies will be required. Notably, the fact that most UT inhibitors do not exhibit hepatotoxicity seems to suggest that UTs play only a minor role in the liver^26–29^. Cytotoxicity assays indicated that M353‑0039 exhibits no toxicity at high concentrations (Supplementary Fig. 5i). In addition, we determined its plasma and renal concentrations, which demonstrated that M353‑0039 accumulated mainly in the kidney 6 h after administration (Supplementary Fig. 6). Therefore, the use of M353-0039 may provide a more convenient and economical pharmaceutical intervention procedure, in addition to specific knockout mice, for studying the unique functions of the UT-A2 subtype without affecting the functions of UT-A3 and UT-B. In the meantime, as a chemical probe derived from virtual screening rather than a lead compound, M353‑0039 warrants careful attention to potential liabilities when used in biological studies.

## Methods

### Ethics statement

Animal experiments were approved by the Institutional Animal Care and Use Committee of Peking University Health Science Center (laboratory animal use license No. SYXK(JING)2021-0064; laboratory animal production license No. SCXK(JING)2021-0013). C57BL/6 mice (male, 8 weeks old) were supplied by the Experimental Animal Center, Peking University (Beijing, China). All animals were fed freely at 22  ±  2 °C with 55%  ±  5% humidity and 12 h/12 h light/dark cycles.

### Virtual screening of UT-A2 inhibitors

To identify potent inhibitors targeting UT-A2, a multi-step, structure-guided virtual screening workflow was performed using Schrödinger software (Schrödinger Suite, New York, NY, USA.). The cryo-EM structure of the human UT-A2 transporter was first prepared using the Protein Preparation Wizard, in which missing residues and side chains were rebuilt, hydrogen atoms were added, and protonation states of titratable residues were assigned to reflect physiological pH (7.0) based on PROPKA calculations^30^, followed by energy minimization under the OPLS4 force field^31^ to relieve potential steric clashes. The initial binding site was defined by integrating known functional annotations of UT-A2 with SiteMap^32^ predictions, enabling identification of an extracellular hotspot pocket characterized by favorable hydrophobic and hydrogen-bonding environments, and a cubic docking grid (15 Å per side) encompassing this region was generated.

The ligand collection originated from a commercial compound library (ChemDiv, ~1 million compounds, downloaded December 2024), from which a target-focused virtual screening library was constructed rather than screening the full database directly. All compounds were first subjected to chemical quality control, including Lipinski’s rule-of-five^33^ filtering, PAINS exclusion^34^, and removal of reactive or potentially toxic functional groups. Structure-guided enrichment was then applied based on analysis of the UT-A2 extracellular pocket and preliminary docking results, retaining compounds with pharmacophore features complementary to the hotspot pocket, such as appropriate hydrogen-bond donor/acceptor functionalities and aromatic or hydrophobic moieties compatible with the extracellular surface. The remaining ~45,000 compounds were clustered using Bemis-Murcko scaffold^35^ definitions with a Tanimoto similarity^36^ threshold of 0.6 to reduce redundancy while preserving chemical diversity, yielding a final target-focused library of ~10,000 representative compounds.

Prior to docking, all ligands were processed using LigPrep(Schrödinger Suite, New York, NY, USA.) to enumerate relevant ionization states and tautomers within a pH range of 7.0 ± 0.5, generate stereoisomers, and perform energy minimization under OPLS4 parameters. Virtual screening proceeded in a tiered manner, with an initial High-Throughput Virtual Screening (HTVS) step applied to rapidly evaluate the library, followed by Standard Precision (SP) docking of the top-ranked compounds for improved pose sampling and scoring, and subsequent Extra Precision (XP) docking of the top candidates using more stringent scoring functions; up to ten binding poses per compound were retained. Post-docking filtering combined predicted binding affinity thresholds with physicochemical criteria consistent with drug-likeness (Lipinski’s rule-of-five and Veber’s rules), and docked poses were visually inspected to assess key hydrogen-bonding and hydrophobic interactions within the hotspot pocket, leading to prioritization of top-ranking compounds for experimental testing.

Based on limited functional inhibition observed in the first-round hits and guided by subsequent structural analysis, a second-round virtual screening workflow was implemented to target a deeper pocket associated with functional channel blockade. Potential affinity-enhancing cavities were identified using the DoGSiteScorer algorithm, revealing a deeper binding region comprising the extracellular urea-binding pocket (EUBP) and an adjacent ATC pocket (ATCP), which were combined and defined as an updated hotspot pocket and visualized in Schrödinger Maestro. Key residues lining this refined pocket (F135, Q231, A270, T275, C285, F287, and A323) were used to guide docking grid construction and compound selection, prioritizing molecules containing polar or aromatic functionalities capable of forming complementary hydrogen-bonding or hydrophobic interactions. Using this updated pocket definition, the top 10,000 compounds from the first-round library were re-docked using Glide^37^ SP and re-scored with Glide XP, after which the top 100 candidates were subjected to visual inspection focusing on interaction patterns with the ATCP and EUBP subpockets and their ability to occupy the deeper channel entrance. Five representative compounds were selected for experimental validation, leading to identification of M353-0039 as a potent and selective UT-A2 inhibitor.

### Docking validation

To validate the docking workflow, retrospective benchmarking was performed using previously reported UT inhibitors, including HQA2 and its analogs (11 compounds), as actives, and 300 physicochemically matched decoys selected from the ChemDiv database following the DUD-E^38^ protocol; docking was carried out using the same Glide SP settings as applied in the virtual screening workflow. Receiver operating characteristic (ROC) curve analysis^39^ yielded an area under the curve (AUC) of 0.82 (Supplementary Fig. 7a), indicating good discrimination between active and inactive compounds, and structural validation by docking HQA2 into the UT-A2 cryo-EM structure reproduced the experimentally observed binding pose with a root-mean-square deviation (RMSD) of 1.9 Å (Supplementary Fig. 7b), which supported the validity of our docking parameters and scoring function. We have also compared the docking-predicted binding modes with the experimentally determined cryo-electron microscopy structures of the UT-A2-E822-1968 and UT-A2-M353-0039 complexes (Supplementary Fig. 7c-d). The results show similarity between the calculated predictions and experimental data. These results confirm that our docking workflow reliably predicts the correct orientation and interaction modes of the two inhibitors prior to experimental structure determination.

### Detection of the inhibitory effect by the urease reaction assay

HEK293F cells (ATCC, CRL-1573) were transfected with wild-type UTs or mutant UTs plasmids by Polyethyleneimine (PEI)^7^. After transfection for 48 h, the cells were resuspended and divided into aliquots. The aliquots were incubated with 50 mmol/L urea, then reach equilibrium by shaking at 200 rpm in 37 °C incubators. After equilibrium, cells were collected by centrifuged at 200 g and washed with HBSS buffer containing corresponding concentration inhibitor for 50 seconds. After washing, the cells were suspended in water and disrupted by ultrasonication at 300 W for 3 min to release the stored urea. To measure the urea levels, we followed the instructions provided in the BUN detection kit (BC1535, Solarbio Life Sciences, Beijing). The procedure started with centrifuging the lysed cells at 25,000 g for 15 min. Next, the supernatant obtained was mixed with Reagent I and Reagent II, and the resulting mixture was incubated at 37 °C for 10 min. Afterward, Reagent III and Reagent IV were added to the mixture, followed by a second incubation at 37 °C for 30 min. Finally, the enzyme-catalyzed reactions were measured using a microplate reader at 630 nm. The urea concentration was quantified by comparing the absorbance values to those of a standard urea solution. The inhibitory effects were then analyzed by fitting the dose-response data to a three-parameter dose-response model using GraphPad software. In addition, we also used the UHPLC-MS/MS method to perform supplementary absolute quantification of intracellular urea concentration after inhibitor treatment. The results showed that following inhibitor application, the intracellular urea concentration exhibited a significant upward trend (Supplementary Fig. 7e-f), which was consistent with the trend observed in the urease assay.

### Detection of the inhibitory effect by the UHPLC-MS/MS assay

To directly confirm the inhibition role of M353-0039, we detected the intracellular urea concentration level by UHPLC-MS/MS method. 5 μL supernatant of lysed 293 F cells as in the urease reaction assay was injected to the Shimadzu LC-30A chromatographic system (Shimadzu, Kyoto, Japan) and separated by a CORTECS® HILIC column (2.1 x 100 mm, 2.7μm). Two mobile phases were used for gradient elution: (A) 0.1 % formic acid in water and (B) acetonitrile. The gradient profile was as follows (time, %B): 0.1 min, 95%; 2 min, 95 %; 5 min, 70%; 15 min, 10%; 15.1 min, 95%; 18 min, 95%with a flow rate of 0.3 mL/min. Rention time of urea was 1.3 min. The column oven temperature was set to 40 °C, and the autosampler temperature was set to 4 °C. Instrument control and data acquisition were performed using Analyst 1.6.3 software (AB Sciex, Foster City, CA), while peak integration was done in MultiQuant (AB Sciex, Foster City, CA). For detection, the HPLC system was coupled to a Sciex QTRAP 4500 with a TurboIonspray interface (TIS). The MS system was operated in positive ionization mode. The MRM transitions of m/z 61–43.9 and were chosen for urea. The optimized mass spectrometric conditions for the urea were as follows: voltage 5000 V, TIS source temperature 450 °C, curtain gas 30 psi, GS1 40 psi, GS2 50 psi, collision energy 15 eV, declustering potential 67 eV. To quantify urea, a linear calibration function was prepared from standard solutions in solvent. The peak areas for urea were used for regression.

### Transwell assay of UT-A1 inhibition

The MDCK cell line stably expressing rat UT-A1 was seeded onto 12 mm Transwell inserts at a density of 2 × 10⁵ cells per well and cultured in a humidified incubator for 4 days^26^, during which a tight monolayer was formed. To eliminate the potential for false-negative results in the MDCK-based assays, we routinely monitored the transepithelial electrical resistance (TEER) of the MDCK cell monolayer before performing inhibition assays. Inhibition experiments were only conducted when the TEER value consistently reached above 1 kΩ·cm², ensuring the formation of a compact, intact, and leak-free cell monolayer. In addition, we included a negative control group (Vehicle) and a positive control group treated with Compound 25a to validate the reliability and feasibility of the experimental system. Test compounds, at a final concentration of 1 μM to 100 μM, were dissolved in 0.01 M PBS containing 10 μM forskolin (FSK), an adenylyl cyclase activator that promotes the translocation of UT-A1 from the cytoplasm to the plasma membrane. The solution was added to both the apical (0.25 mL) and basolateral (1 mL) chambers of the transwell and incubated for 30 minutes at 37 °C. After incubation, the basolateral solution was removed and replaced with PBS containing 15 mmol/L urea, with all other components unchanged. From the apical chamber, 5 μL of solution was collected at time points of 0, 1, 3, 5, 10, 15, 20, 30, 40, 50 and 60 minutes. Urea concentrations in the apical samples were quantified using a urea assay kit. The urea concentration-time curve was plotted using GraphPad Prism 8, and the initial rate of urea transport was determined by calculating the slope of the linear portion of the curve. Considering the rigorous urease reaction assay using 293 F cell-based system and the difficulty of UT-A2 expression in MDCK cell, we did not assess the response of the inhibitors on UT‑A2 in the MDCK-based assay.

### Erythrocyte lysis assay for determining UT-B inhibition activity

After blood was collected from male C57BL/6 mice via eyeball extraction, it was transferred into anticoagulant tubes and centrifuged at 400 g for 15 minutes^26^. The supernatant was discarded, and isotonic PBS (0.01 M PBS containing 5 mM glucose) was added to the red blood cell (RBC) pellet at a volume ten times that of the pellet. The mixture was gently resuspended and centrifuged again at 400 g for 15 minutes; this washing step was repeated twice. The RBCs were then diluted with hypertonic PBS (0.01 M PBS containing 5 mM glucose and 1.25 M urea) to obtain a 2% hematocrit suspension, which was incubated at room temperature for 2 h with gentle inversion every 30 minutes. Subsequently, 100 μl of the RBC suspension was transferred to a 96-well plate, and 1 μl of 25a or E822-1968 dissolved in DMSO was added to each well to achieve final concentrations of 10 nM, 100 nM, 1 μM, 10 μM and 100 μM. Negative and positive control wells received 1 μl of DMSO without compound. The plate was mixed using a microplate shaker and incubated at room temperature for 6 min. Then, 20 μl of RBC suspension from each well was quickly transferred into a black 96-well plate containing 180 μl of isotonic PBS (positive control wells contained 180 μl of double-distilled water), mixed thoroughly by pipetting, and the absorbance at 710 nm was measured within 5 min using a spectrophotometer. The percentage of RBC lysis (%RBC) was calculated using the formula: %RBC = 100% × (A_negative - A_sample) / (A_negative - A_positive). The IC_50_ values of the compounds were determined using GraphPad Prism software.

### Cytotoxicity assay

MDCK cells were seeded at a density of 1 × 10^4^ cells/100 µl per well in a 96-well plate. When the cells reached ~50% confluence, they were exposed to 100 µM M353-0039, while solvent control wells received DMEM containing DMSO, for 24 h. After treatment, 10 µl of CCK-8 solution was added to each well and incubated for 1 h, followed by measurement of absorbance at 450 nm. Cell viability was calculated using the following formula: Cell viability (%) = 100% × (OD_test - OD_blank) / (OD_ctrl - OD_blank), where OD_test, OD_blank, and OD_ctrl represent the absorbance values of the test, blank control, and solvent control wells, respectively.

### Expression and purification of UT-A2 proteins in SF9 cells

The recombinant baculovirus of UT-A2 was constructed using the Bac-to-Bac Baculovirus Expression System (Invitrogen) as previous reported^7,40^. In summary, the baculovirus was prepared using FuGENE HD transfection reagent (Promega). SF9 (Expression Systems, 94-001 F) insect cells were cultured in ESF921 medium at a density of 2.5 × 10⁶ cells/ml and subsequently infected with UT-A2 baculovirus. Cells were incubated for 48 h at 27 °C with shaking at 110 rpm, then collected via centrifugation, flash-frozen in liquid nitrogen, and stored at -80 °C. For purification, the cells were resuspended in lysis buffer (20 mM HEPES, pH 7.4, 100 mM NaCl, 5 mM CaCl₂, 2.5 mg/ml leupeptin, and 0.2 mg/ml benzamidine) and homogenized into membranes using a Dounce homogenizer. The cell lysate was subsequently solubilized with 0.5% (w/v) lauryl maltose neopentylglycol (LMNG; Anatrace) and 0.1% (w/v) cholesteryl hemisuccinate TRIS salt (CHS; Anatrace) for 2 h at 4 °C. The supernatant was obtained by centrifugation at 25,000 g for 30 min, and the solubilized sample was incubated with M1 anti-FLAG resin for 2.5 h at 4 °C. The sample solution was immobilized on Flag-M1 resin, loaded on a Flag-M1 column, and washed with 20 column volumes of 20 mM HEPES, pH 7.4, 100 mM NaCl, 5 mM CaCl_2_, 0.01% (w/v) LMNG, 0.002% (w/v) CHS, 2.5 mg/ml leupeptin and 0.2 mg/ml benzamidine. The protein solution was then eluted with 20 mM HEPES, pH 7.4, 100 mM NaCl, 2.5 mg/ml leupeptin, 0.2 mg/ml benzamidine, 0.0005% (w/v) LMNG, 0.0001% (w/v) CHS, 10 mM EDTA and 0.2 mg/ml FLAG peptide (GL Biochem). The protein solution was collected and concentrated. To form the complex between the inhibitor and UT, 50 μM of inhibitor was individually added to the concentrated sample solution described above and incubated on ice for a minimum of 30 min. The protein was then applied to a Superose 6 Increase 10/300 GL column (Cytiva) equilibrated with a buffer containing 20 mM HEPES (pH 7.4), 100 mM NaCl, 0.00075% (w/v) LMNG, 0.0002% (w/v) CHS, and 50 μM inhibitor. The eluted protein fractions were subsequently concentrated using a 100 kDa molecular weight cutoff (MWCO) Millipore concentrator.

### Cryo-grid preparation and EM data collection

For cryo-EM grid preparation, the purified UT-A2 protein was concentrated to 3.0 mg/ml and applied in 3 μl drops onto glow-discharged holey carbon grids. The grids were then transferred to a Titan Krios electron microscope (300 kV) fitted with a spherical aberration (Cs) corrector. Images were captured using a Falcon counting camera (Thermo Fisher Krios G4 Falcon 4i) at a nominal magnification of ×130,000, resulting in a pixel size of 0.92 Å. The automated low‑dose image acquisition was enabled by the Thermo Scientific EPU software (version 3.7, Thermo Fisher). Movie stacks were collected with a defocus range of -0.8 to -2.0 μm, an accumulated dose of 60 electrons per Å^2^, and a total of 40 frames per micrograph.

### Image processing and 3D reconstruction

The initial cryo-EM movie stacks of E822-1968-UT-A2 complex were summed and subjected to CryoSPARC (Version 4.7)^41^. A total of 6746 movie stacks were imported into CryoSPARC. We applied the patch motion correction and patch CTF estimation in CryoSPARC to preprocess the micrographs. Then, we selected micrographs with an average defocus value between 8,000 and 20,000, which were further inspected to remove images contaminated by crystalline ice or other visible contaminants. From 500 high‑quality micrographs, particles were randomly picked and then subjected to 2D classification. Classes deemed suitable upon visual inspection were extracted, bundled as templates, and used as the seed particles for Topaz training. A conventional neural network-based method, Topaz^42^, was implemented in CryoSPARC to ensure the selection of suitable particles. A total of 2,131,097 particles were extracted and subjected to 2D classification. A stack of 154,178 clean particles from 2D classification was selected and subjected to ab initio reconstruction. We then used the Remove Duplicate Particles tool in CryoSPARC to eliminate duplicate particles. Finally, 116,881 particle projections from the best class were subjected to nonuniform refinement with C3 symmetry to generate a density map with a global resolution of 2.9 Å for E822-1968-UT-A2 complex at a Fourier Shell Correlation (FSC) level of 0.143. We calculated the local resolution distribution maps using the Local Resolution Estimation module in CryoSPARC, which were visualized with Chimera^43^. The calculation of M353-0039-UT-A2 complex was done by the same processes.

### Model building and structure refinement

The initial template of UT-A2 structures were derived from the previous UT structures (PDB: 8XD9, 8XD7) using Phyre online server^44^. Models were rigidly docked into the cryo-EM density map and saved as new models with the coordinates relative to cryo-EM map using UCSF Chimera^43^. All separated coordinates were merged into one PDB file by manual building and adjustment in COOT^45^. The final model was subjected to global refinement and minimization in real space using PHENIX^46^ for cryo-EM maps. The figures were prepared with UCSF Chimera and PyMOL (http://pymol.org/).

### The binding energy calculation

To explore the binding energy of UT inhibitors to the UT-A2, the UT-A2 model were preparation in the Desmond (Schrödinger 2015.2) to set up the membrane in the Predefine SPC solvent model with an 10x10x10 Å^3^ orthorhombic box as previous reported^21^. The membrane model was set up as POPC in 300 K with 0.15 M NaCl added for charge neutralization and the force filed was set to OPLS3. The inhibitors were prepared using Schrödinger Maestro software. Minimizations of the inhibitors were carried out using the OPLS3 force field module. The binding energy calculations employed the Prime MM-GBSA (Schrödinger 2015.2) in which residues within 4.5 Å, 5.0 Å, 5.5 Å of the inhibitors were allowed to flex while minimizing the complex with the with the OPLS3 force field and VSGB solvation model^47^. The MMGBSA-dG-binding energy were applied for statistical analysis.

### In vivo selective function of UT-A2 inhibitors

Wild type male C57BL/6 J mice, aged 8 weeks, were placed in metabolic cages for 2 days for adaptation. During the adaptation period, sufficient water and food were provided, and urine was collected every 24 h. For the normal diet group, after the adaptation period, mice were divided into the treatment group and the control group based on urine volume. For the low-protein diet group, the diet was switched to low-protein food after the acclimatization period, and urine was collected every 24 h. After 7 days, mice were grouped into treatment and control groups based on urine volume during the low-protein diet period. Mice were subjected to complete water deprivation while being provided with sufficient food, and the diet type remained unchanged. After 30 h of water deprivation, the control group received 0.1 ml/10 g of solvent, while the treatment group received 0.1 ml/10 g of UT-A2 inhibitor (8 mg/ml). After 30 h of water deprivation, the residual urine was extruded by gently bladder and abdomen were gently massage to avoid influence on UT-A2 inhibitor evaluation. Then after 6 h of UT-A2 inhibitor administration, the urine was collected by gently bladder and abdomen massage again to measure urine urea concentration and urine osmolarity. After dissection, renal medulla tissue was collected in an EP tube, weighed using a balance, and homogenized with 10 volumes of double-distilled water using a homogenizer. The homogenate was then centrifuged at 13000 g for 15 minutes, and the supernatant was collected. Urea concentrations in the samples were measured using a urea assay kit, and osmolality was determined using a freezing point osmometer.

### Detection of the inhibitory effect of M353-0039 on HepG2 cells

HepG2 cells (ATCC, HB-8065) were digested down with trypsin and washed with HBSS buffer. We then centrifuged and discarded the supernatant, added the appropriate amount of HBSS to resuspend the cells, divided cells into aliquots. The aliquots were incubated with 300 mmol/L urea, then reach equilibrium by culturing in 37°C incubators for 20 min. After centrifugation, cells were collected by centrifuged at 200 g and washed with HBSS buffer containing inhibitors at a final concentration of 10 μM for 5 min. After centrifugation, the supernatant (reflecting extracellular urea levels) was aspirated into a new tube for analysis, and the cell sediment was lysed with 100 μl of water. After lysis, the cell was disrupted by ultrasonication at 300 W for 3 min to release the stored urea, the urea concentration in solution at this point represents the intracellular urea concentration. To measure the intracellular and extracellular urea levels, we followed the instructions provided in the BUN detection kit (BC1535, Solarbio Life Sciences, Beijing) as described before.

### Determination of the in vivo concentrations of M353-0039

Wild type male C57BL/6 mice, aged 12 weeks, were allowed to free access to food and water in a controlled environment of 22 ± 2 °C, and maintained under a 12 h light/dark cycle. Mice received a single dose of vehicle or M353-0039 at 80 mg/kg body weight via intraperitoneal injection respectively. At 6 h after dosing, blood samples were collected, anti-coagulated, the supernatant plasma was preserved after centrifuging and kept at -20 °C until use. The kidneys were rinsed with saline, blotted, weighted, and homogenized (5 vol/weight saline), and stored at -20 °C until use.

The analyte M353-0039 were dissolved in MeOH and gradient-diluted to generate working solutions. Then the working solutions were mixed with 9 volume of blank mouse plasma or kidney homogenate to establish calibration standards, with concentration of 10 to 2000ng/ml. The berberine dissolved in MeOH was used as internal standard (IS) at 500 ng/ml. To measure the concentrations of M353-0039 in blood and kidney, 100 μL plasma or 200 μL kidney homogenate were mixed with ten percent volume of IS berberine, and 1 ml ethyl acetate, vortexed, centrifuged at 3000 g for 7 min. The supernatants were nitrogen flushed, re-dissolved in 100 μL (for plasma sample) or 150μL (for kidney sample) acetonitrile: water (8: 2), and vortexed, centrifuged again, the processed supernatants were subjected to LC-MS analysis.

The analysis was conducted by an Agilent 6500 Series Q-TOF LC/MS coupled with UPLC, the chromatographic separation was conducted in an Eclipse Plus C18 RRED 1.8μm column at 28 °C with a flow rate of 0.4 ml/min. The mobile phrases were 0.1% (v/v) formic acid in water (A) and pure acetonitrile (B). The gradient elution program was as follows: 80% A, 0 min; 80% A, 2 min; 40% A, 4 min; 80% A, 5 min. An electrospray ionization (ESI) source in positive-ion mode was employed to quantify the analyte with the transitions of m/z from 100 to1000. The optimized fragmentor voltage is 175 V.

### Reporting summary

Further information on research design is available in the Nature Portfolio Reporting Summary linked to this article.

## Supplementary information

Supplementary Information

Reporting Summary

Transparent Peer Review file

## Source data

Source Data

## Data Availability

All data produced or analyzed in this study are included in the main text or the supplementary information. The cryo-EM density maps and atomic coordinates of E822-1968-UT-A2 complex, M353-0039-UT-A2 complex have been deposited at the Electron Microscopy Data Bank under accession codes EMD-65636, EMD-65637 and the Protein Data Bank under accession codes 9W4K9W4L. Molecular Dynamics trajectories have been uploaded to the Figshare repository (10.6084/m9.figshare.30404923). Source data are provided with this paper as a Source Data file. Source data are provided with this paper.
