# Supplementary material for: Hotspot pocket-based discovery of urea transporter selective inhibitors
Source: Nat Commun. 2026 Apr 20;17:5444. doi: 10.1038/s41467-026-71834-w (PMC13279936; doi:10.1038/s41467-026-71834-w)
Supplement: Supplementary file 1 — Supplementary Information [file 41467_2026_71834_MOESM1_ESM.pdf]

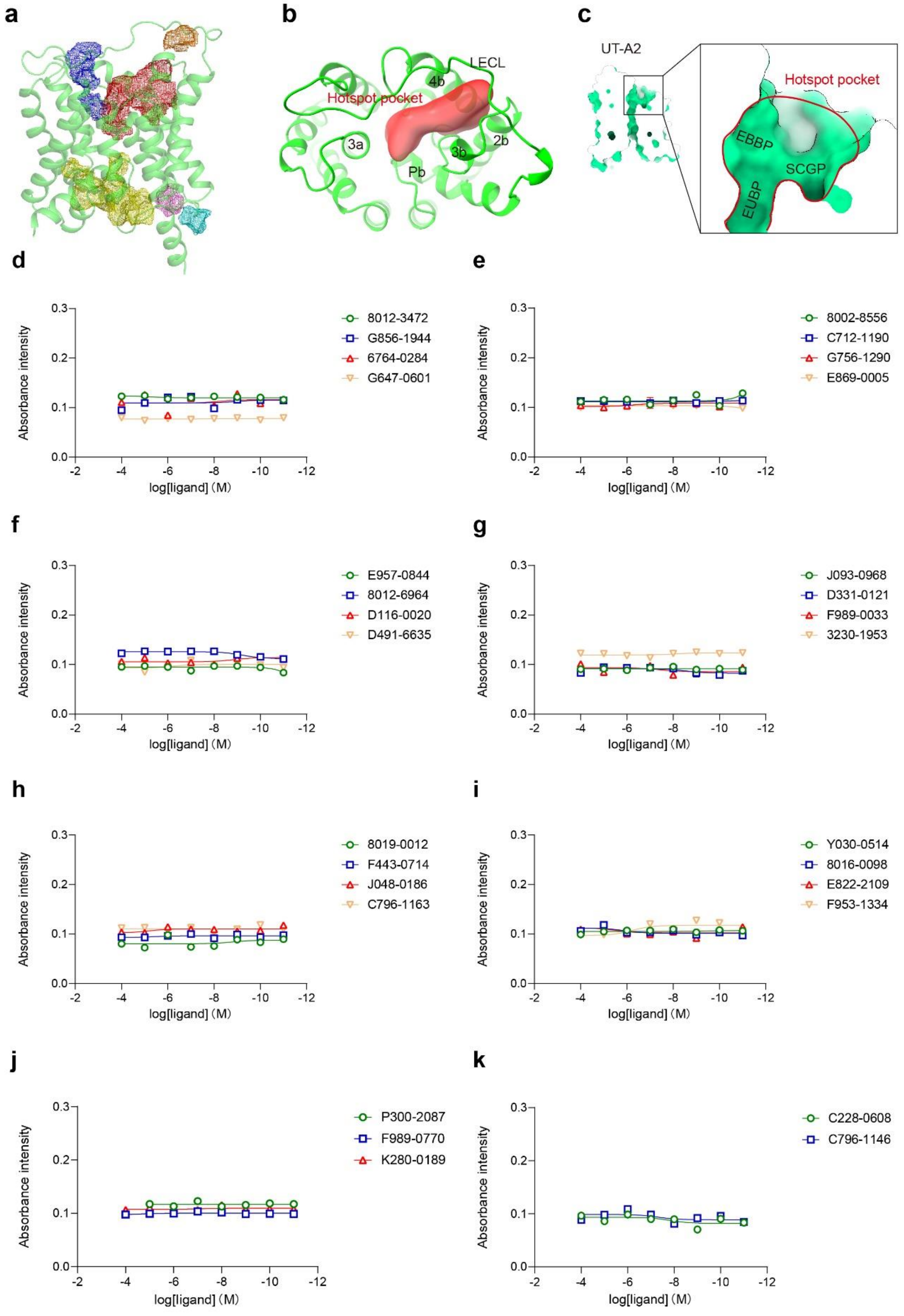

Supplementary Fig. 1 Hotspot pocket-based virtual screening for UT-A2.

a. The predicted ligand binding pockets on the UT-A2 predicted by DoGSiteScorer.

b. The identified hotspot pocket on UT-A2 is surrounded by the extracellular lateral α-helices

3a, 2b-4b, Pb and the loop LECL.

c. Cutway view of the hotspot pocket on UT-A2, which includes the regions of extracellular urea binding pocket (EUBP), extracellular blocker binding pocket (EBBP) and the "SCG" pocket (SCGP).

d-k. The dose dependent curves of the other 29 compounds in blocking urea transport mediated by UT-A2 using urease reaction assay. Values are mean ± SEM from three independent experiments (n = 3).

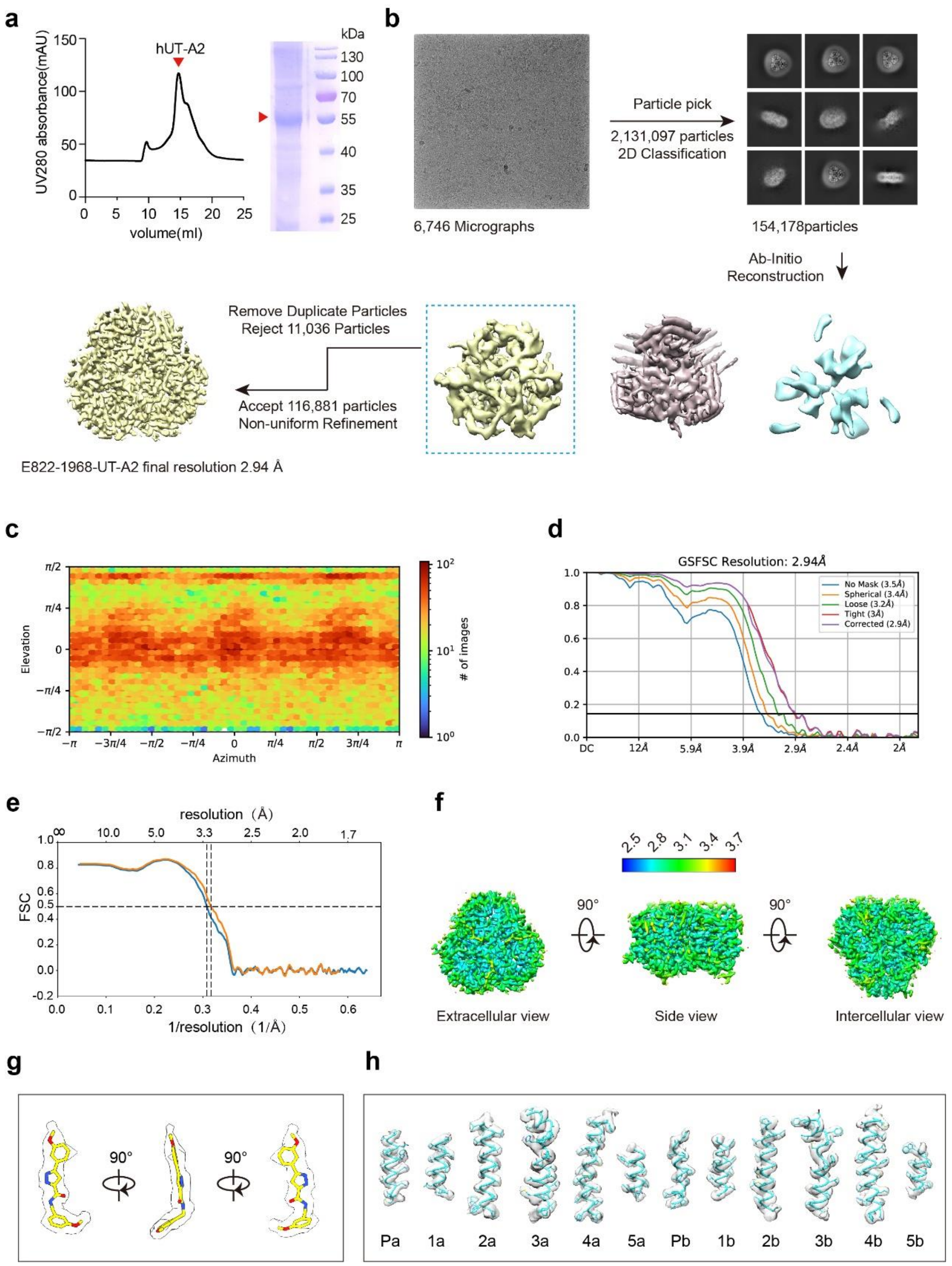

Supplementary Fig. 2 Calculation processes of the E822-1968-UT-A2 complex.

a. Representative size-exclusion chromatography elution profiles of the purified UT-A2 complex. The elution volume of size-exclusion chromatography peak of UT-A2 was at approximately 14.8 ml, indicating a homotrimer states. The Coomassie brilliant blue staining of UT-A2 was shown on the right.

b-d. Calculation processes of the E822-1968-UT-A2 structure using the cryoSPARC. Representative cryo-EM micrograph, 2D class averages and the refined map of E822-1968-hUT-A2 structure were shown (**b**) with the orientation distribution histogram figures (**c**) and the Fourier shell correlation curves (**d**) for the final 3D density map.

e. The map-model FSC curves of E822-1968-UT-A2 complex validated by Phenix.

f. The map of E822-1968-UT-A2 complex was further colored according to local resolution (Å).

g-h. The cryo-EM density maps and models were shown for the E822-1968 (**g**) and the main helices of E822-1968-UT-A2 structure (**h**).

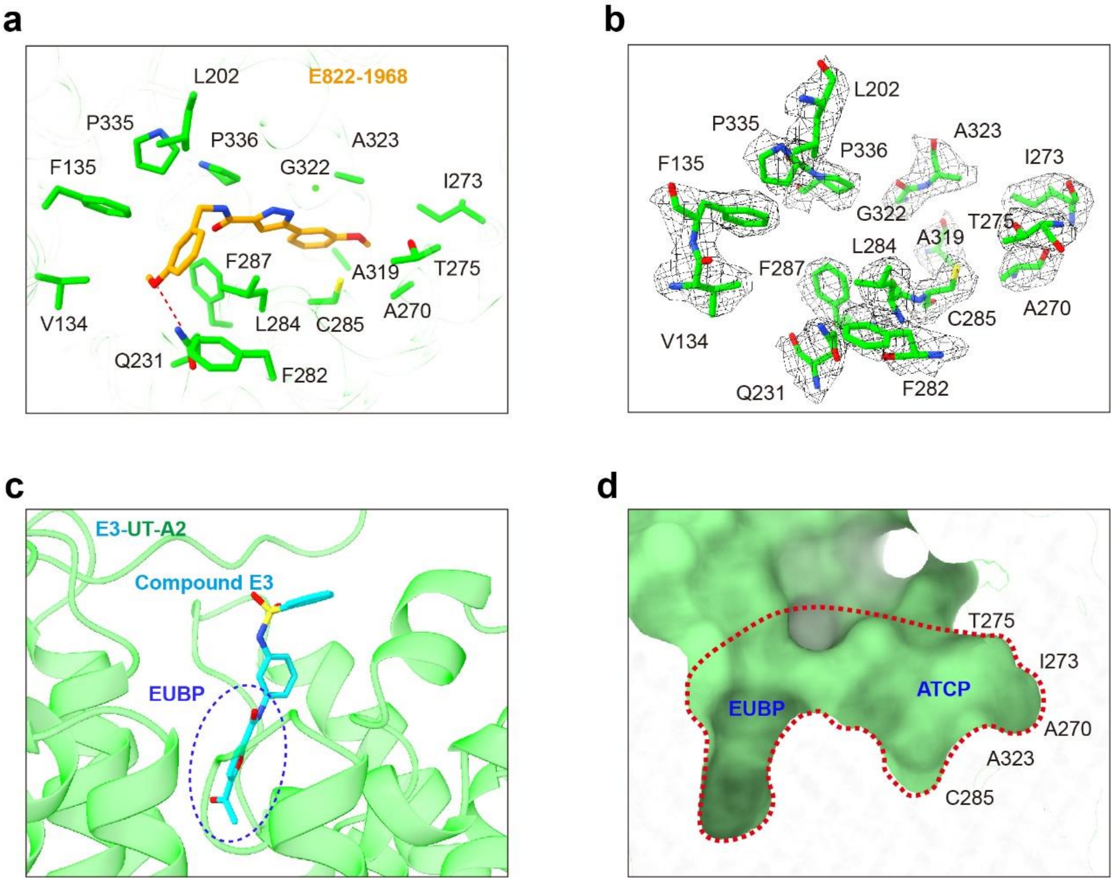

Supplementary Fig. 3 The binding pocket of E822-1968 and the refreshed hotspot pocket.

a. The key residues of UT-A2 in E822-1968 binding pocket are shown. The hydrogen bond is shown as red dash line.

b. The cryo-EM density (gray mesh) of the key residues of the E822-1968 binding pocket at the contour level of 0.220 V in UCSF Chimera.

c. The compound E3 interacts with the EUBP (blue dashed circle) of UT-A2.

d. The refreshed hotspot pocket is primarily localized to the regions associated with EUBP and ATCP.

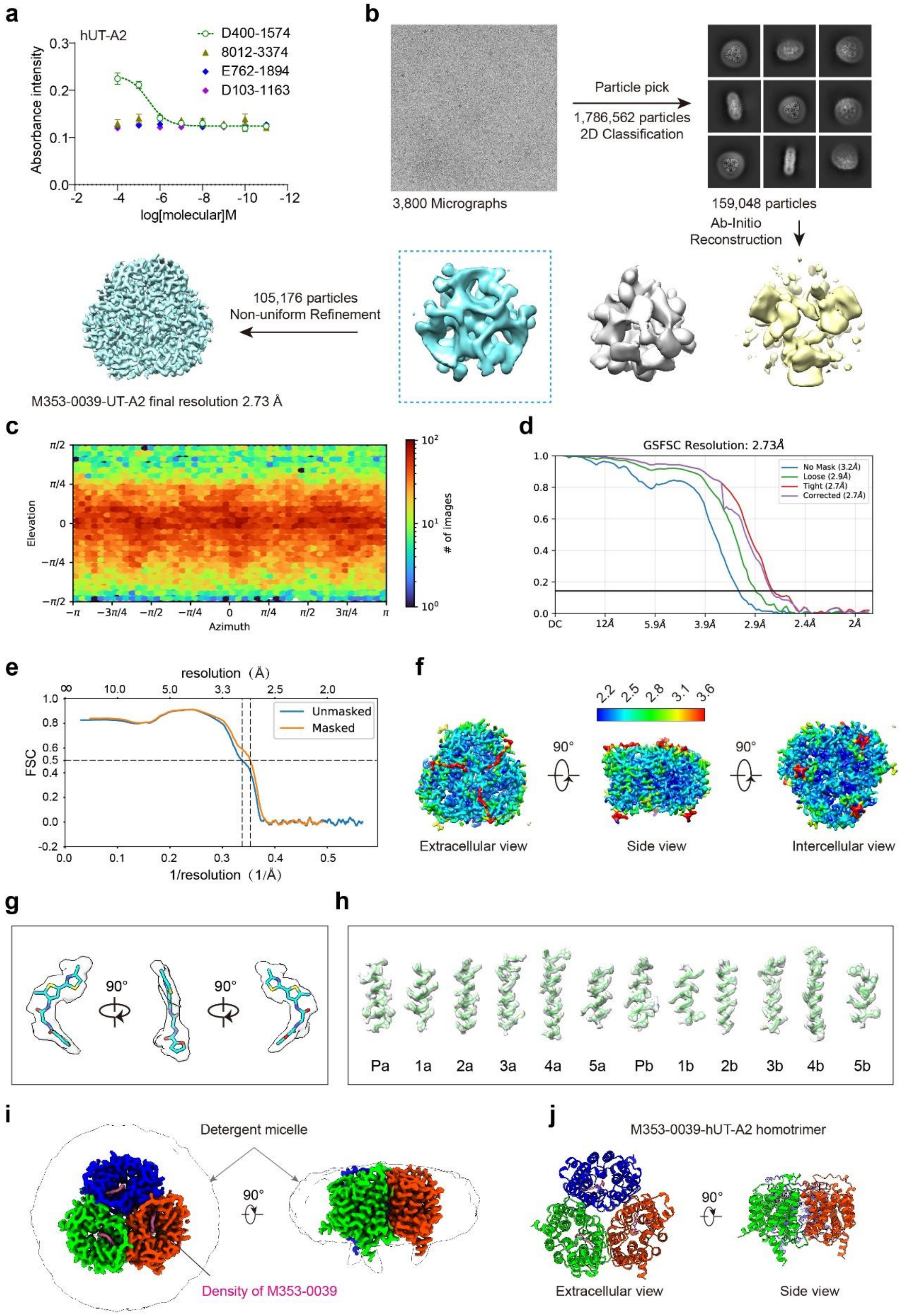

Supplementary Fig. 4 Calculation processes of the M353-0039-UT-A2 complex.

a. The dose dependent curves of the other candidate compounds for UT-A2 selective inhibitors in rescreening. The compound D400-1574 showed inhibitory potency to UT-A2 with IC_{50} value of 3.42 ± 0.96 μM. Values are mean ± SEM from three independent experiments (n = 3).

b-d. Calculation processes of the M353-0039-UT-A2 structure using the cryoSPARC. Representative cryo-EM micrograph, 2D class averages and the refined map of M353-0039-UT-A2 structure were shown (**b**) with the orientation distribution histogram figures (**c**) and the Fourier shell correlation curves (**d**) for the final 3D density map.

e. The map-model FSC curves of M353-0039-UT-A2 complex validated by Phenix.

f. The map of M353-0039-UT-A2 complex was further colored according to local resolution (Å).

g-h. The cryo-EM density maps and models were shown for the M353-0039 (**g**) and the main helices of M353-0039-UT-A2 structure (**h**).

i. Cryo-EM densities of M353-0039-UT-A2 homotrimer from the extracellular view (left) and the side view (right), respectively. The EM densities of UT homotrimer are color green, blue and red surrounded by the detergent micelle. The EM densities of inhibitor M353-0039 bound to the extracellular sides of UT-A2 are shown with violet color.

j. Structural representation of M353-0039-UT-A2 homotrimer from the extracellular view (left) and the side view (right), respectively.

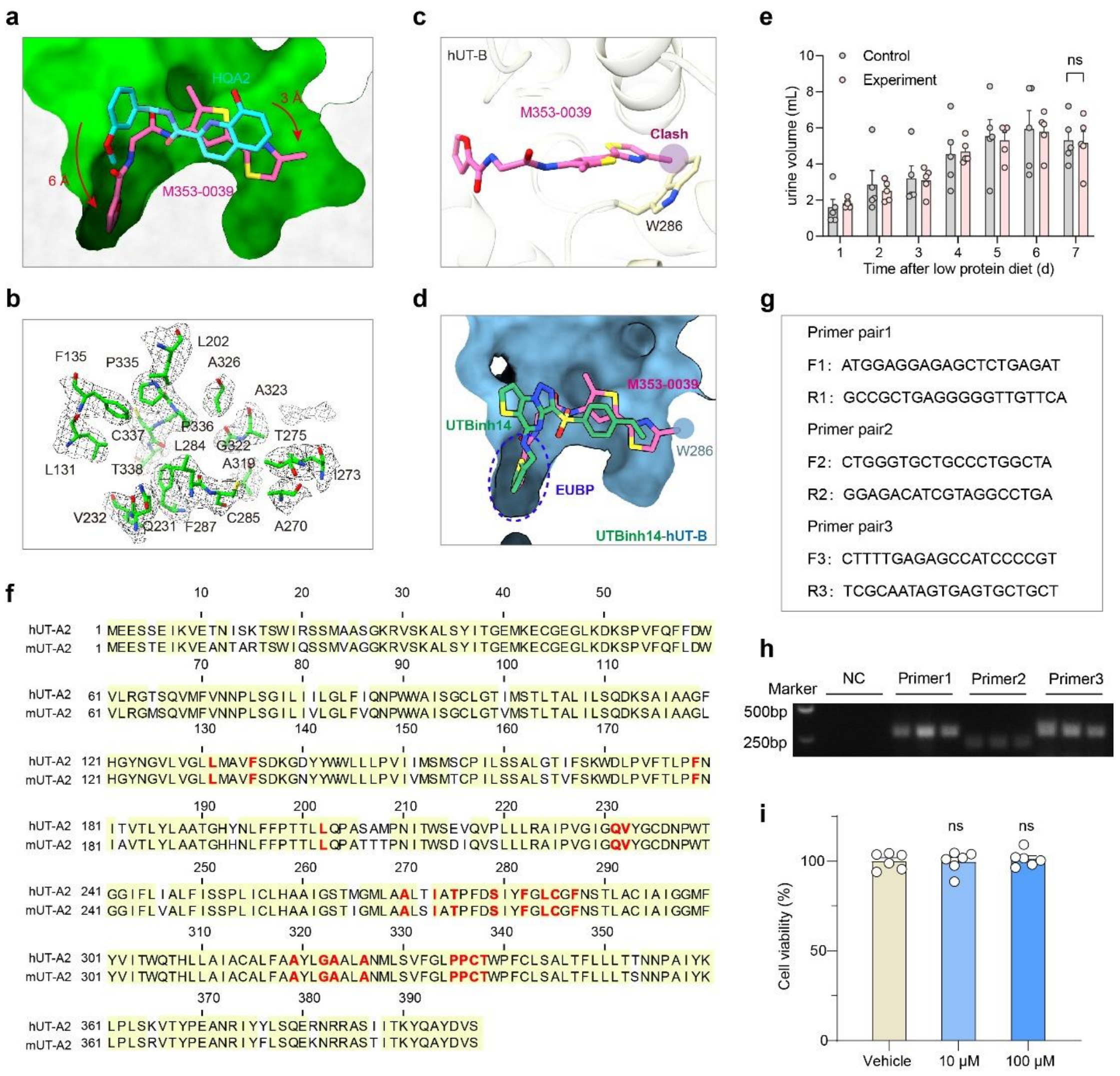

Supplementary Fig. 5 Structural basis of M353-0039's selective interaction with UT-A2.

a. Compared with HQA2, the furan ring of M353-0039 is 6 Å deeper into the urea transport channel, and the thiazole ring is 3 Å deeper on the non-channel side.

b. The cryo-EM density (gray mesh) of the key residues in UT-A2 of the M353-0039 binding pocket at the contour level of 0.120 V in UCSF Chimera.

c. The W286 of UT-B cause steric hindrance (purple dot) to bind with M353-0039.

d. Comparison between UTBinh14 (green sticks) and M353-0039 (magenta sticks) in the ligand binding pocket in UTBinh14-UT-B complex (gray blue). The 2-thienyl group of UTBinh14 inserts into EUBP (blue-dashed line circle), which is similar to M353-0039, but fails to form hydrogen bond with the tryptophan on position 286 (W286, gray blue dot).

e. The urine volume of mice treated with low protein diet. ns, no significant; statistical differences were determined by the two-sided unpaired Student's t-test. Data were shown as

the mean ± SEM (n=5).

f. Amino acid sequence alignment of human UT-A2 and mouse UT-A2. The same amino acids were shown as light yellow background. The residues of the binding pocket (colored red) of M353-0039 in such two different species are completely identical.

g. Three primer pairs for qPCR detection of human UT-A2 expression in HepG2 cell.

h. The electrophoresis band of qPCR product of HepG2 cell UT-A2 expression. NC, negative control, use ultrapure water as the template and primer 1 as the primer for qPCR.

i. The CCK-8 cytotoxicity assay results indicated that M353-0039 treatment at the tested concentrations did not show significant cytotoxicity. Statistical differences were determined by the two-sided unpaired Student's t-test compared with the vehicle.

a

Animal	Blood (ng/ml)	Kidney (μg/g)
1	16.74	0.17
2	19.67	0.59
3	7.29	0.44
Mean±SEM	14.57±3.74	0.40±0.12

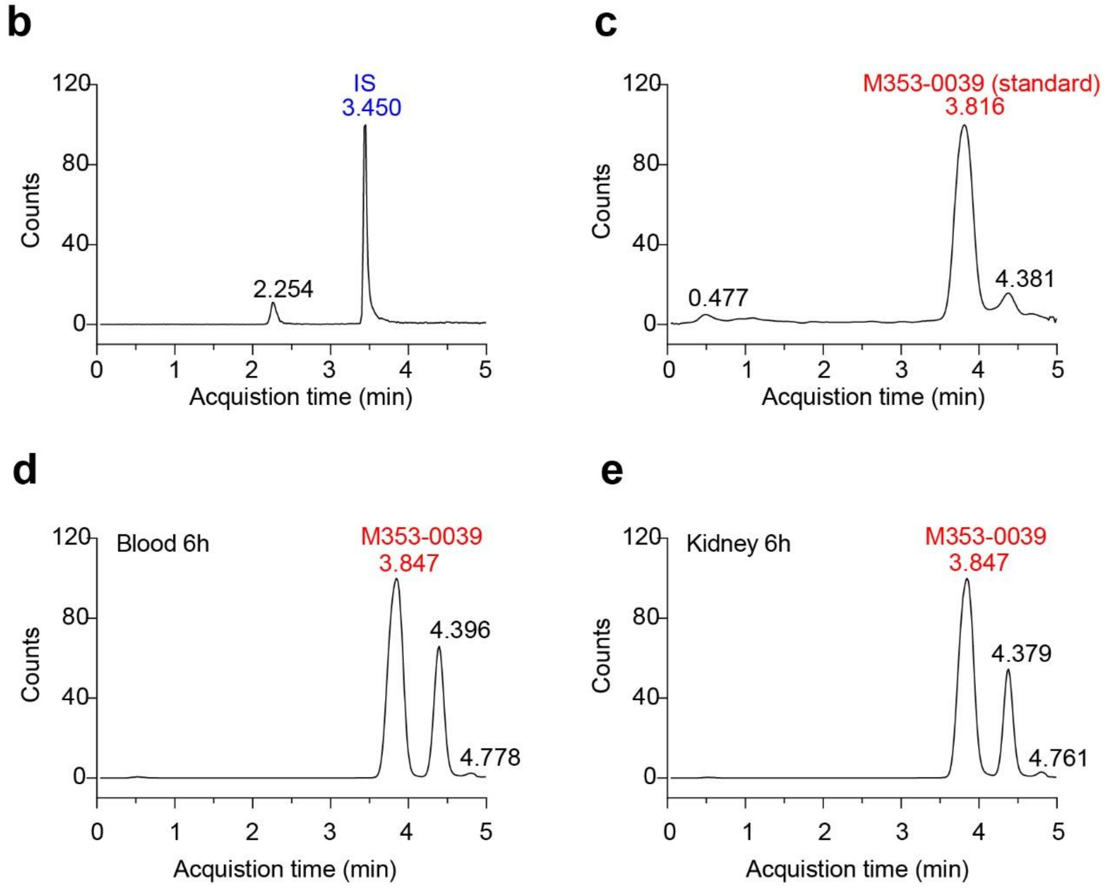

Supplementary Fig. 6 The concentration of M353-0039 in mouse blood and kidney after 6 h intraperitoneal administration.

a. The calculated M353-0039 concentration of blood and kidney sample from three mice.

b. Representative UPLC chromatogram of Internal standard berberine.

c. Representative UPLC chromatogram of M353-0039 standard.

d. Representative UPLC chromatogram of M353-0039 in blood sample after 6 h administration.

e. Representative UPLC chromatogram of M353-0039 in kidney sample after 6 h administration.

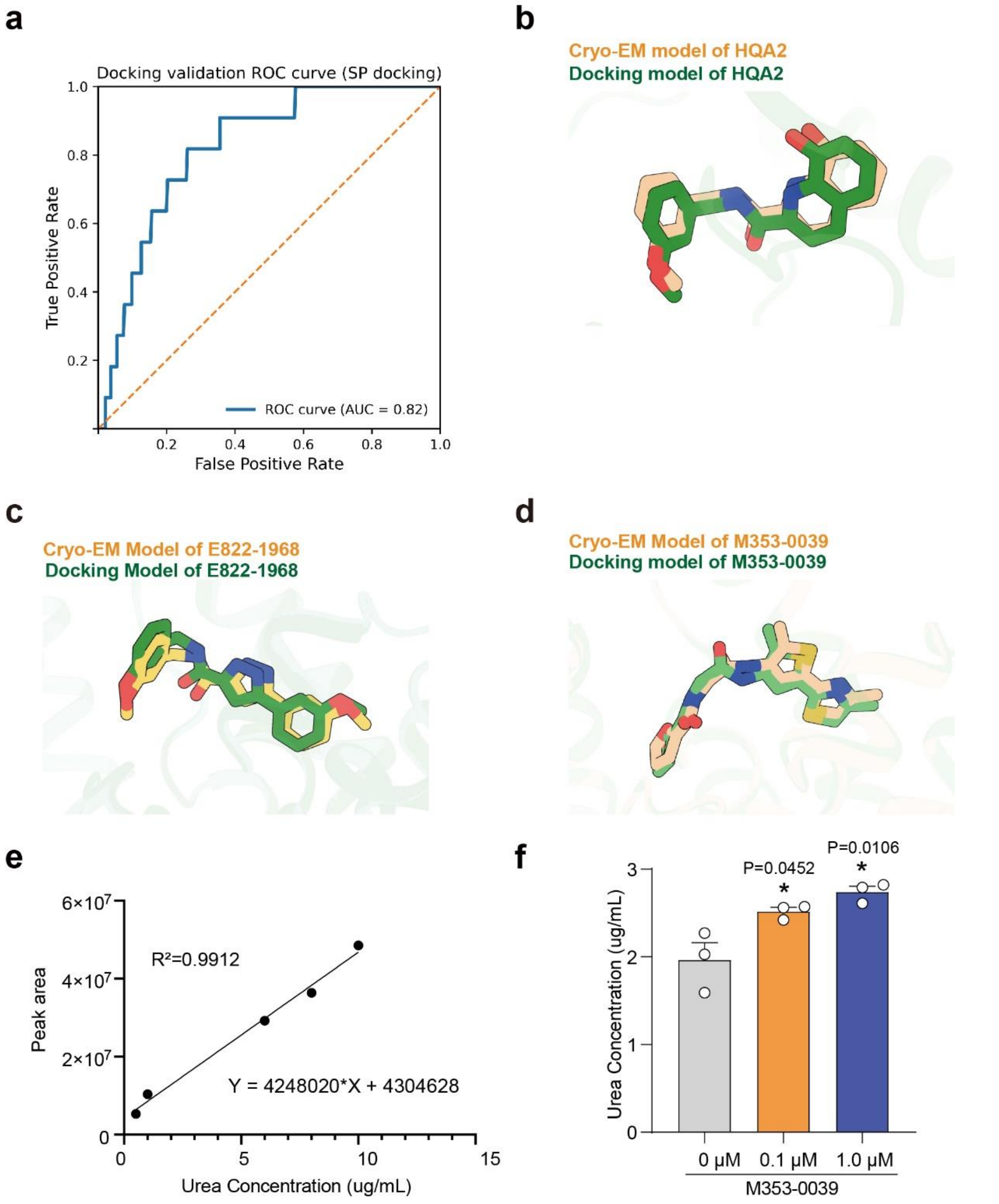

Supplementary Fig. 7 Validation of docking performance and the urease reaction assay.

a. Receiver operating characteristic (ROC) curve evaluating the performance of SP docking in distinguishing active compounds from decoys. The area under the curve (AUC) is 0.82, indicating good discriminatory power of the docking protocol.

b. Structural comparison of ligand HQA2 in the cryo-EM model (orange) and the corresponding docking-predicted binding pose (green). The overall conformation and orientation of the ligand are highly consistent between the experimental structure and the docking model.

c. Comparison of the cryo-EM-derived pose (orange) and the docking-predicted pose (green)

of ligand E822-1968.

d. Comparison of the cryo-EM-derived pose (orange) and the docking-predicted pose (green) of ligand M353-0039.

e. Standard curve of peak area vs. urea concentration.

f. 293F cells overexpressing UT-A2 were incubated with 50 mmol/L urea and washed with HBSS containing various concentrations of M353-0039; M353-0039 treatment markedly elevated intracellular urea levels. *$P < 0.05$, One-way ANOVA with Tukey's test (compared with the 0 μM). Values are mean ± SEM from three independent experiments (n = 3).

Supplementary Table 1. The diverse pockets of urea transporter UT-A2 for inhibitor interaction.

Pockets	Universal name	Description	Reference
EUBP	The extracellular urea binding pocket	This urea molecule binding pocket is located in the region of the UT-A2 urea transport channel near the extracellular side, primarily comprising the amino acids L131, F179, Q231, V232, F287, C337 and T338.	Nat Commun. 2024 Nov 26;15(1):10226.
EBBP	The extracellular blocker binding pocket	Located on the extracellular side of urea transport channel in the UT molecular, the primary binding region for inhibitors includes two sub-pockets, EBBP1 and EBBP2, primarily comprising the amino acids F135, L202, F282, L284, P335, and P336.	Nat Commun. 2024 Nov 26;15(1):10226.
SCGP	"SCG" pocket	This pocket is completely independent of the urea transport channel of UT-A2 and is composed of T275, S279, C285, G322 and A326 from UT-A2. The residues of the SCG pocket region are not conserved among different UTs, particularly at positions S279, C285 and G322, making it a potential target pocket for developing UT-selective inhibitors.	Nat Commun. 2024 Nov 26;15(1):10226.
ATCP	"ATC" pocket	Located on the extracellular side of UT-A2 and independent of both the urea transport channel and the EBBP region, this subpocket is enclosed by residues A270, I273, T275, C285, and A323.	This article
"T-T" pocket	The binding pocket of the thiophene-thiazole group	Located on the extracellular side of UT-A2 and independent of its urea transport channel, this region serves as the binding site for the thiophene-thiazole group of inhibitor M353-	This article

		0039, involving residues A326, A319, C285, T275, A270, I273, G322, A323, L284 and P336.	
L-P pocket	L-P pocket	The region near L202 and P335 on the EBBP of UT-A2, where these two residues are not conserved across different UT subtypes, represents a potential binding pocket for developing UT-selective inhibitors.	Nat Commun. 2024 Nov 26;15(1):10226.

Supplementary Table 2. Details of the molecules involved in developing UTA2-selective inhibitors.

Molecule	Universal name	Structural formula	Description	Reference
E822; E822-1968;	3-(3-methoxyphenyl)-N-[(3-methoxyphenyl)methyl]-1H-pyrazole-5-carboxamide	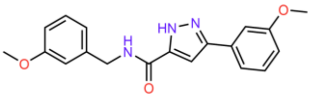	This study reports E822-1968 with IC_{50} values of 0.44 ± 0.03 μM for UT-A2 and 3.42 ± 0.35 μM for UT-A3, respectively.	This article
M353;M353-0039	N-(2-{[2-methyl-5-(4-methyl-1,3-thiazol-2-yl)thiophen-3-yl]amino}-2-oxoethyl)furan-2-carboxamide	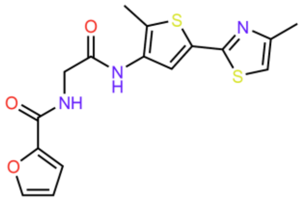	This study reports M353-0039 with an IC_{50} of 0.35 ± 0.03 μM against UT-A2.	This article
25a	N-(4-acetamidophenyl)-5-acetylfuran-2-carboxamide	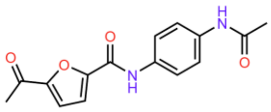	This UT inhibitor is reported the IC_{50} values of 0.14 μM for rat UT-B, 0.48 μM for mouse UT-B and 0.58 μM for human UT-A2.	Eur J Med Chem.15:226:113859 Nat Commun. 15(1):10226.
HQA2	8-hydroxy-N-(3-methoxybenzyl)quinoline-2-carboxamide	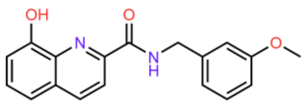	Mildly selective UT-A selective inhibitors. This UT inhibitor is reported the IC_{50} of 5.3 μM against UT-A1, 3.66 ± 0.09 μM against UT-A2 with less inhibition against UT-B.	Chem Biol. 20(10):1235-44.; Nat Commun. 15(1):10226.

E3	5-acetyl-N-[3-(phenylsulfonamido)phenyl]furan-2-carboxamide	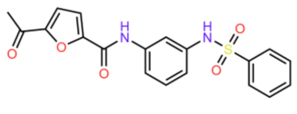	This UT inhibitor is reported the IC_{50} values of 0.22 ± 0.03 μM for UT-A2 and 0.52 ± 0.02 μM for UT-A3, respectively.	Acta Pharmacol Sin. 46(11):2989-2997.; Biomedicines. 13(4):992.
D400-1574	N-{2-[3-(4-methoxyphenyl)-1,2,4-oxadiazol-5-yl]phenyl}-1-(2-methylphenyl)-5-oxopyrrolidine-3-carboxamide	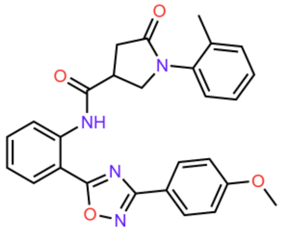	This study reports D400-1574 with an IC_{50} of 3.42 ± 0.96 μM against UT-A2.	This article
E762-1894	2-ethyl-N-[(5-methylfuran-2-yl)methyl]-2,3-dihydro[1,4]oxazino[2,3,4-hi]indole-5-carboxamide	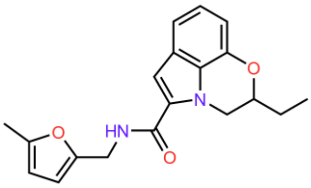	The molecular shows no inhibitory effect on UTs.	This article
8012-3374	2-[(5-amino-1,3,4-thiadiazol-2-yl)sulfanyl]-1-([1,1'-biphenyl]-4-yl)ethan-1-one	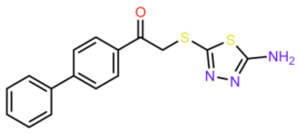	The molecular shows no inhibitory effect on UTs.	This article
D103-1163	7-methyl-N-[2-(4-methylbenzoyl)-1-benzofuran-3-yl]-4-oxo-4H-1-benzopyran-2-carboxamide	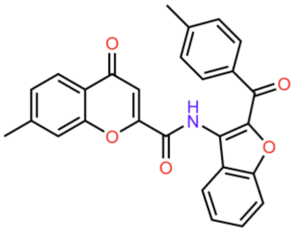	The molecular shows no inhibitory effect on UTs.	This article

Supplementary Table 3. Small molecule screening data.

Category	Parameter	Description
Assay	Type of assay	Cell-based
	Target	UT-A2
	Primary measurement	Detection of absorbance by microplate reader at 630nm

	Key reagents	BUN detection kit (BC1535, Solarbio Life Sciences, Beijing)
	Assay protocol	HEK293F cells were transfected with wild-type UTs or mutant UTs plasmids by Polyethyleneimine (PEI). After transfection for 48 h, the cells were resuspended and divided into aliquots. The aliquots were incubated with 50 mmol/L urea, then reach equilibrium state by shaking at 200 rpm in 37℃ incubators. After equilibrium, cells were collected by centrifuged at 200 g and washed with HBSS buffer containing corresponding concentration inhibitor for 50 seconds. After washing, the cells were suspended in water and disrupted by ultrasonication at 300W for 3 minutes to release the stored urea. To measure the urea levels, we followed the instructions provided in the BUN detection kit (BC1535, Solarbio Life Sciences, Beijing). The procedure started with centrifuging the lysed cells at 25,000 g for 15 minutes. Next, the supernatant obtained was mixed with Reagent I and Reagent II, and the resulting mixture was incubated at 37°C for 10 minutes. Afterward, Reagent III and Reagent IV were added to the mixture, followed by a second incubation at 37°C for 30 minutes. Finally, the enzyme-catalyzed reactions were measured using a microplate reader at 630 nm. The urea concentration was quantified by comparing the absorbance values to those of a standard urea solution. The inhibitory effects were then analyzed by fitting the dose-response data to a three-parameter dose-response model using GraphPad software.
	Additional comments	Huang SM, Huang ZZ, Liu L, Xiong MY, Zhang C, Cai BY, Wang MW, Cai K, Jia YL, Wang JL, Zhang MH, Xie YH, Li M, Zhang H, Weng CH, Wen X, Li Z, Sun Y, Yi F, Yang Z, Xiao P, Yang F, Yu X, Tie L, Yang BX, Sun JP. Structural insights into the mechanisms of urea permeation and distinct inhibition modes of urea transporters. Nat Commun. 2024 Nov 26;15(1):10226.
Library	Library size	30
	Library composition	Compound, SMILES: 8012-3472 (O=S(=O)(Cc1ccccc1)c1cc(Oc2ccccc2)cc(Oc2ccccc2)c1C#N), G856-1944 ([O-][N+](=O)c1ccc(cc1)-n1ccnc1SCC(=O)Nc1ccc2OCOc2c1), 6764-0284 (COc1ccccc1\C=C\C=c1/sc2nc(=O)c(Cc3ccc(Cl)cc3)nn2c1=O), G647-0601 (Cn1c(SCc2cc(=O)n3nc([nH]c3n2)-c2ccccc2)nnc1-c1cccs1), 8002-8556 (Nc1cccc(Oc2ccc3C(=O)N(C(=O)c3c2)c2cccc(N)c2)c1), C712-1190 (O=C(NCc1cccs1)c1cc2COc3ccccc3-c2s1), G756-1290 (Cc1ccn2c(c1)nc(COc1cc(NC(=O)Nc3ccc4OCOc4c3)ccc1C)cc2=O), E869-0005 (O=C(c1ccccc1)c1ccc2nc(Nc3ccc4OCOc4c3)c3nncn3c2c1), E957-0844 (COc1ccc(cc1)N1CCN(CC1)C(=O)c1oc2ccccc2c1NC(=O)c1ccc(F)cc

		1), 8012-6964[O-][N+](=O)c1cccc(c1)-n1sc2c3C(=O)c4ccccc4C(=O)c3ccc2c1=O D491-6635Brc1ccc(s1)-c1nsc(NC(=O)c2cc(=O)c3ccccc3o2)n1 D116-0020 O=C(c1cccs1)n1nc(nc1NCc1cccs1)-c1ccco11NC(=O)C1CN(C(=O)C1)c1ccccc1C), J093-0968 (FC(F)(F)c1cc(nc2nc(nn12)C(=O)NCc1cccs1)-c1cccs1), D331-0121 (Fc1ccc(CNc2nc(nn2C(=O)COc2ccccc2)-c2ccco2)cc1), F989-0033 (COC(=O)c1ccc(NC(=O)CSc2nc3ccccc3n3cnnc23)cc1), 3230-1953 (COc1ccc(NC2=C(Cl)C(=O)N(C2=O)c2ccc(Br)cc2)cc1), 8019-0012 (COc1ccc(NC(=O)C(=O)NCc2ccc(Br)o2)cc1OC), F443-0714 (Fc1cccc(CNC(=O)c2ccc(cc2)-n2nc(OCc3cccc(F)c3)ccc2=O)c1), J048-0186 (Clc1ccc(cc1)-c1ccc(CCC(=O)NCc2ccccc2)o1), C796-1163 (Fc1ccc(Cn2nc(cc2C(=O)NCc2cccs2)-c2cccs2)cc1), Y030-0514 (COc1ccc(CNC(=O)CCCOc2ccc(Cl)cc2Cl)cc1), 8016-0098 (Nc1nn(nc1C(=O)OCc1cccc(F)c1)-c1ccccc1), E822-2109 (Cc1ccc(CNC(=O)c2cc(n[nH]2)-c2ccccc2)cc1), F953-1334 (Cc1c(oc2c(C)cccc12)-c1nnc(o1)C(=O)NCc1cccs1), P300-2087 (Fc1ccc(Cn2c3cc(ccc3n3nccc3c2=O)C(=O)NCc2cccc(F)c2)cc1), F989-0770 (COc1cccc(CNC(=O)CSc2nc3ccccc3n3c(nnc23)-c2ccccc2)c1), K280-0189 (Cc1ccc(CNC(=O)CSc2nc3ccccc3c3nc(nn23)-c2ccccc2)cc1), C228-0608 (COc1ccc(CNC(=O)c2cc(nc3ccccc23)-c2ccc(Cl)s2)cc1), C796-1146 (Cc1ccc(CNC(=O)c2cc(nn2Cc2ccc(F)cc2)-c2cccs2)cc1), E822-1968 (COc1cccc(CNC(=O)c2cc(n[nH]2)-c2cccc(OC)c2)c1).
	Source	ChemDiv compound library (San Diego, CA, USA)
	Additional comments	> 90% purity
Screen	Format	96-well round bottom transparent plate
	Concentration(s) tested	10pM-100μM compound
	Plate controls	standard urea solution
	Reagent/ compound dispensing system	Manual dispensing using multi-pipettes
	Detection instrument and software	Tecan Austria GmbH INFINITE 200 PRO microplate reader, Tecan i-control 2.0
	Assay validation/QC	Previous reported UT-A2 inhibitor 25a
	Correction factors	no
	Normalization	no
	Additional comments	Stock solution 50mM in DMSO
Post-HTS	Hit criteria	Dose-dependent inhibition of urea transmembrane transport

analysis		
	Hit rate	3.3%
	Additional assay(s)	Erythrocyte lysis assay
	Confirmation of hit purity and structure	^{1}H NMR spectra
	Additional comments	Full screening results are displayed in Supplementary Fig.1d-k.

Supplementary Table 4. The ^{1}H NMR spectrogram of compound E822-1968 and M353-0039.

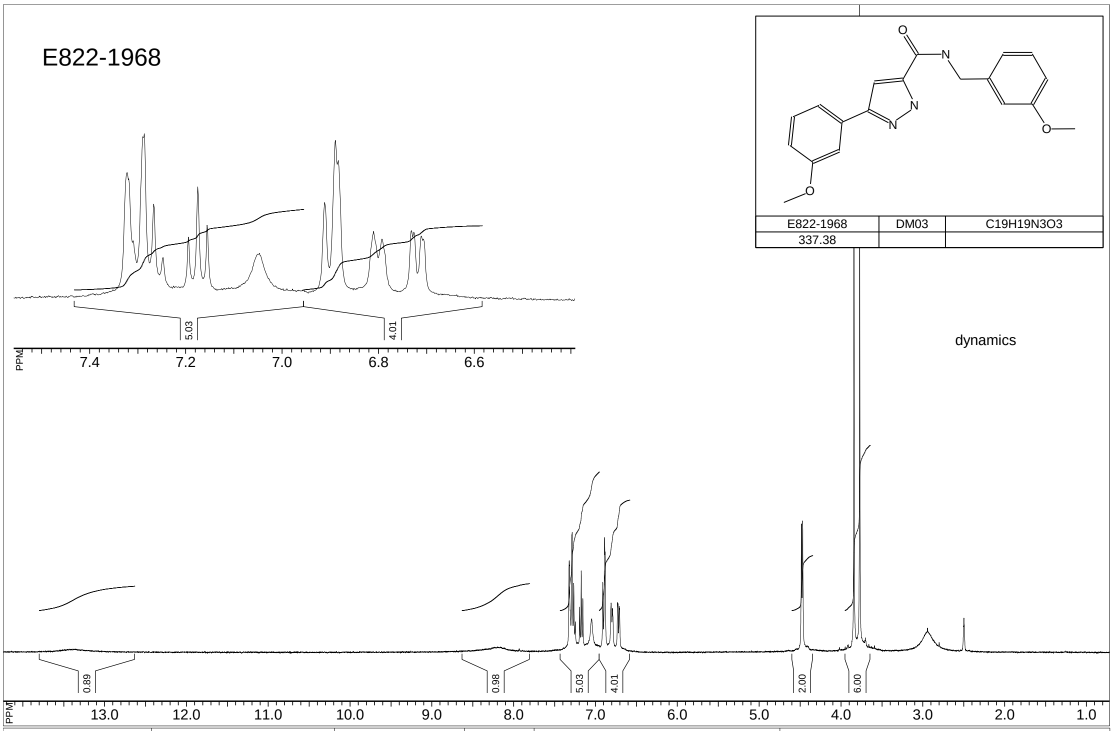

**File name: E822-1968**	Operator: PEB	SF: 399.9525 MHz	NSC: 0	PW: 12.00 usec, RG: 28, SI: 16384	**Grade: OK(00)**
Date: 04-Aug-2004	Solvent: DMSO-d6 + CCl4	SW: 7502 Hz	TE: 300 K	AQ: 1.09 sec, RD: 1.50 sec	***E822-1968.04-Aug-2004.00***

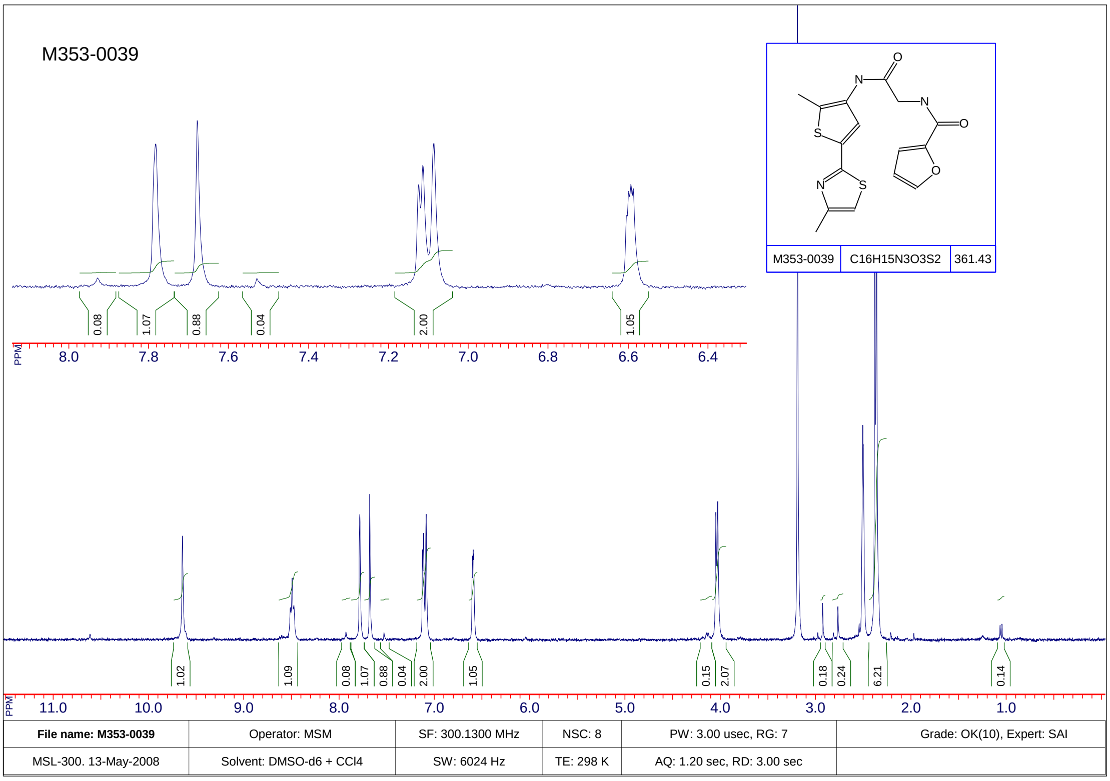

Supplementary Table 5. Data collection and refinement statistics of the inhibitors bound UT-A2 complex

	**E822-1968-UT-A2**	**M353-0039-UT-A2**
Magnification	130,000	130,000
Voltage (kV)	300	300
Electron exposure ($e^{-}/Å^{2}$)	60	60
Defocus range (μm)	-0.8 ~ -2.0	-0.8 ~ -2.0
Pixel size (Å)	0.92	0.92
Symmetry imposed	C3	C3
Initial particle images (no.)	2,131,097	1,786,562
Final particle images (no.)	116,881	105,176
B-factors of map-sharping	-99.2	-94.7
Map resolution (Å)	2.9	2.7
FSC threshold	0.143	0.143
Map resolution range (Å)	2.7 ~12.3	2.2 ~9.9
Initial model used (PDB code)	8XD9, 8XD7	8XD9, 8XD7
**Model composition**		
Non-hydrogen atoms	7620	7656
Protein residues	990	990

Ligands	3	3
Q-score	0.5570	0.5670
**B factors (Å^2)**		
Protein	58.67	51.86
Ligand	57.68	54.96
**R.m.s. deviations**		
Bond lengths(A)	0.009	0.008
Bond angles (°)	1.293	1.218
**Validation**		
MolProbity score	2.24	1.65
Clash score	9.11	9.05
**Ramachandran Plot**		
Favored	95.43	97.05
Allowed	4.75	2.95
Disallowed	0	0

Supplementary Table 6. The interactions between E822-1968 and UT-A2

Residues	Interaction	Distance (Å)
V134	Hydrophobic contacts≤4.0 Å	3.7
L202		3.5
A270		3.7
I273		3.7
F282		3.7
L284		3.6
F287		3.8
A319		3.3
G322		3.4
A323		3.4
P335		3.9
P336		3.6
F135	π-π interaction	3.4
T275	Polar interaction ≤4.0 Å	3.9

C285		3.6
Q231	Hydrogen Bond ≤3.5 Å	3.0

Supplementary Table 7. The interactions between M353-0039 and UT-A2

Residues	Interaction	Distance (Å)
L131	Hydrophobic contacts≤4.0 Å	3.5
F135		3.4
L202		3.7
V232		3.6
A270		3.8
I273		4.0
T275		3.6
L284		3.5
F287		3.6
A319		3.2
G322		3.7
A326		3.7
P335		4.0
P336		3.7
C337		3.7
T338		3.8
Q231	Polar interaction ≤4.0 Å	3.7
A323		3.9
C285	Hydrogen Bond ≤3.5 Å	3.5
