# Supplementary material for: Hotspot pocket-based discovery of urea transporter selective inhibitors
Source: Nat Commun. 2026 Apr 20;17:5444. doi: 10.1038/s41467-026-71834-w (PMC13279936; doi:10.1038/s41467-026-71834-w)
Supplement: Supplementary file 2 — Reporting Summary [file 41467_2026_71834_MOESM2_ESM.pdf]

nature portfolio

Corresponding author(s): Shenming Huang

Last updated by author(s): Mar 6, 2026

Reporting Summary

Nature Portfolio wishes to improve the reproducibility of the work that we publish. This form provides structure for consistency and transparency in reporting. For further information on Nature Portfolio policies, see our Editorial Policies and the Editorial Policy Checklist.

Statistics

For all statistical analyses, confirm that the following items are present in the figure legend, table legend, main text, or Methods section.

n/a	Confirmed	
☐	☒	The exact sample size (*n*) for each experimental group/condition, given as a discrete number and unit of measurement
☐	☒	A statement on whether measurements were taken from distinct samples or whether the same sample was measured repeatedly
☐	☒	The statistical test(s) used AND whether they are one- or two-sided<br>*Only common tests should be described solely by name; describe more complex techniques in the Methods section.*
☒	☐	A description of all covariates tested
☒	☐	A description of any assumptions or corrections, such as tests of normality and adjustment for multiple comparisons
☐	☒	A full description of the statistical parameters including central tendency (e.g. means) or other basic estimates (e.g. regression coefficient) AND variation (e.g. standard deviation) or associated estimates of uncertainty (e.g. confidence intervals)
☐	☒	For null hypothesis testing, the test statistic (e.g. *F*, *t*, *r*) with confidence intervals, effect sizes, degrees of freedom and *P* value noted<br>*Give P values as exact values whenever suitable.*
☒	☐	For Bayesian analysis, information on the choice of priors and Markov chain Monte Carlo settings
☒	☐	For hierarchical and complex designs, identification of the appropriate level for tests and full reporting of outcomes
☒	☐	Estimates of effect sizes (e.g. Cohen's *d*, Pearson's *r*), indicating how they were calculated

Our web collection on statistics for biologists contains articles on many of the points above.

Software and code

Policy information about availability of computer code

Data collection	Thermo Scientific EPU
Data analysis	CryoSPARC v4.5; PHENIX version 1.14-3260-000, WinCoot 0.8.9, Pymol 1.7, ImageJ 1.8.0, GraphPad Prism 8.0, chimera-1.18-win64, ChimeraX-1.8

For manuscripts utilizing custom algorithms or software that are central to the research but not yet described in published literature, software must be made available to editors and reviewers. We strongly encourage code deposition in a community repository (e.g. GitHub). See the Nature Portfolio guidelines for submitting code & software for further information.

Data

Policy information about availability of data

All manuscripts must include a data availability statement. This statement should provide the following information, where applicable:

- Accession codes, unique identifiers, or web links for publicly available datasets
- A description of any restrictions on data availability
- For clinical datasets or third party data, please ensure that the statement adheres to our policy

All data produced or analyzed in this study are included in the main text or the supplementary materials. The cryo-EM density maps and atomic coordinates of E822-1968-UT-A2 complex, M353-0039-UT-A2 complex have been deposited at the Electron Microscopy Data Bank under accession code EMD-65636, EMD-65637 and the Protein Data Bank under accession code 9W4K, 9W4L. Molecular Dynamics trajectories have been uploaded to the Figshare repository [https://doi.org/10.6084/m9.figshare.30404923].

Research involving human participants, their data, or biological material

Policy information about studies with human participants or human data. See also policy information about sex, gender (identity/presentation), and sexual orientation and race, ethnicity and racism.

Reporting on sex and gender	This study does not involve human data.
Reporting on race, ethnicity, or other socially relevant groupings	This study does not involve human data.
Population characteristics	This study does not involve human data.
Recruitment	This study does not involve human data.
Ethics oversight	This study does not involve human data.

Note that full information on the approval of the study protocol must also be provided in the manuscript.

Field-specific reporting

Please select the one below that is the best fit for your research. If you are not sure, read the appropriate sections before making your selection.

[x] Life sciences [ ] Behavioural & social sciences [ ] Ecological, evolutionary & environmental sciences

For a reference copy of the document with all sections, see nature.com/documents/nr-reporting-summary-flat.pdf

Life sciences study design

All studies must disclose on these points even when the disclosure is negative.

Sample size	The sample size was determined based on general standards for experimental cell biology, which were performed as indicated in related Figure legends.
Data exclusions	No data were excluded from the analysis.
Replication	For cell-based functional assays, each experiment with sufficient reproducibility was repeated independently at least three times.
Randomization	Since this is not an animal or clinical case/control study, randomization was attempted or needed. Due to the quantitative nature of biological research studies, no covariates were considered and the independent covariates tested were sufficient for the functional interpretation in this study.
Blinding	Blinding was not necessary or possible for either structure determination or cell-based functional assays, because in most cases, data were collected and analyzed by the same individual investigators.

Reporting for specific materials, systems and methods

We require information from authors about some types of materials, experimental systems and methods used in many studies. Here, indicate whether each material, system or method listed is relevant to your study. If you are not sure if a list item applies to your research, read the appropriate section before selecting a response.

Materials & experimental systems

n/a	Involved in the study	
[x]	[ ]	Antibodies
[ ]	[x]	Eukaryotic cell lines
[x]	[ ]	Palaeontology and archaeology
[ ]	[x]	Animals and other organisms
[x]	[ ]	Clinical data
[x]	[ ]	Dual use research of concern
[x]	[ ]	Plants

Methods

n/a	Involved in the study	
[x]	[ ]	ChIP-seq
[x]	[ ]	Flow cytometry
[x]	[ ]	MRI-based neuroimaging

Eukaryotic cell lines

Policy information about cell lines and Sex and Gender in Research

Cell line source(s)	HEK293 cells(ATCC,CRL-1573); Spodoptera frugiperda cell (Sf9)(Expression Systems,94-001F)；HepG2 cell(ATCC,HB-8065)

Authentication	All of the cell lines are maintained by the supplier. No additional authentication was performed by the authors of this study.
Mycoplasma contamination	All of the cell lines used in this study were tested and verified negative for mycoplasma contamination.
Commonly misidentified lines (See ICLAC register)	None

Animals and other research organisms

Policy information about studies involving animals; ARRIVE guidelines recommended for reporting animal research, and Sex and Gender in Research

Laboratory animals	C57BL/6J, about 8 weeks old
Wild animals	None
Reporting on sex	Male
Field-collected samples	Under ethically permissible conditions, animal data were collected in accordance with experimental requirements.
Ethics oversight	Animal experiments were proven by the Institutional Animal Care and Use Committee of Peking University Health Science Center (laboratory animal use license No. SYXK(JING)2021-0064; laboratory animal production license No. SCXK(JING)2021-0013).

Note that full information on the approval of the study protocol must also be provided in the manuscript.

Plants

Seed stocks	None
Novel plant genotypes	None
Authentication	None
