# Supplementary material for: Hotspot pocket-based discovery of urea transporter selective inhibitors
Source: Nat Commun. 2026 Apr 20;17:5444. doi: 10.1038/s41467-026-71834-w (PMC13279936; doi:10.1038/s41467-026-71834-w)
Supplement: Supplementary file 3 — Transparent Peer Review file [file 41467_2026_71834_MOESM3_ESM.pdf]

nature portfolio

Peer Review File

# Hotspot pocket-based discovery of urea transporter selective inhibitors

Corresponding Author: Professor Shenming Huang

**This file contains all reviewer reports in order by version, followed by all author rebuttals in order by version.**

Version 0:

Reviewer comments:

Reviewer #1

(Remarks to the Author)
This is a data rich and extremely technical manuscript that identifies and provides evidence for an apparently specific inhibitor of the UT-A2 urea transporter. The methodology appears sound but the manuscript could be improved by adding some details, defining some acronyms, and enhancing the discussion.
Specific suggestions:
Introduction
In introduction, the authors disclose the relationship between UT-B and extrarenal pathology. They also say that UT-B shares about 40% sequence difference with UT-A2.
They then suggest that inhibition the sequence similarities between UT-B and UT-A2 might represent an opportunity for inhibitors of UT-A2 to have off target extra-renal effects. This is offered as justification for developing inhibitors with high specificity for UT-A2.
Results
Line 112 is there a definition for the “SCG” pocket?
Line 135 the initial mention of HQA2 requires a reference that provides the initial description.
Line 156 is confusing. “into an extended subpocket out of the ebbp…. Consider rewording. Ie., into a subpocket contiguous with but outside of the EBBP and SCG pockets.
Line 159 “direct interactions with 16 amino acids of E822-1068 The inhibitor is not composed of amino acids so the wording makes understanding very difficult. Revise to say interactions between 16 amino acids in the UT-A2 pockets with E822-1068 …
The descriptions of the specific amino acid side chain bindings to the rings and R groups of the inhibitors are very detailed and informative, although they make a complicated read.
Line 264-267 After describing the UT-A2 knockout mice the description of the next experiment (low protein, water deprivation) needs to specify what mice are being treated to be compared to the UT-A2 mice. Insert “control” or “wild type’ or similar to clarify.
Concerning the experiment with low protein diet +/- M353 cmpd. Clearly there is an effect of the inhibitor, but urine volume is not explained and would impact the interpretation of these data. In a water deprivation study there would be drastic changes in urine volume and would vary depending on the protein load.
Diacussion
The discussion is limited. The first part essentially restates the key points in the results section without extending these results into a deeper discussion of the potential meaning of these results. For example, UT-A2 is essentially identical with one half of the UT-A1 molecule. A discussion of how the inhibitor fails to inhibit UT-A1 while successfully inhibiting UT-A2 could be explored. The potential for the MDCK testing medium for UT-A1 inhibition to provide a potential false negative. What happens if you put UT-A2 in MDCK cells and repeat the inhibition protocol? Would this provide more rigor to the data.
In the discussion of in vivo effects of the inhibitor, the authors again just restate the results observed without exploring the potential explanation for the observations. Why is there an apparent difference between the normal protein load and the low protein load in the observed effect of the inhibitor? Also, explain why the animals were water deprived?
Figures & Legends
Figure 1
Multiple spelling errors in Figure 1a
1b, refine the English (ie., Finally, 1 hit was identified to have UT-A2 inhibitory activity) Again, define the phrase “SCG” pocket.

1c, Just to be sure, the 30 compounds were selected based on their binding to the UT-A2 target (this is the 'selective filter' used to choose these potential inhibitors? And with all 30 showing binding capacity, only 1 compound show ANY inhibitory activity? Either the heatmap is an inadequate way to show the background inhibition by these other compounds, or it calls into question the selection methods. This one out of the 30 binding protein result should be addressed/explained in the discussion.
1d. nice clear information
Figure2
2a change centrifugal to centrifugation. Identify the type of cells that are being studied.
2b Line 672, type-0 IC5 should be IC50
2c the color for hUT-A3 is too light. Please change to something darker.
2e the positive control "25a" is not described either in the legend or the methods section. It needs to be added to the methods and properly referenced
2f if cmpd E822 was synthesized from HQA2, then fine. If not, remove the green arrow between the compound structures.
2g Most of this data is from previous published work. Was UT-A1 actually measured in this work? Since it shows that it was not measured with the E822 compound but was not detected with the M353 compound. If the assay was performed with the M353 compound, why are the results that were positive (UT-A2) not shown?
Figure 3
3e. This reviewer is not an expert in special analysis of molecules so these images are difficult to interpret, however, the legend text might include a color description of the two compounds that are being compared (ie., HQA2 is drawn in turquoise while E822 is rendered in gold) to help the novice reader.
3g Again, EBBP is defined, but SCG and ATC are not. Be sure they are defined earlier in the manuscript as they appear to be crucial to the mechanism of inhibition that is the object of this manuscript. Also, strongly suggest that the "SCG pocket" be colored something other than green as it disappears against the overall green background of the protein structure.
In addition, the T-T pocket is not described or defined other than "another binding pocket" but since it appears to be important in distinguishing effectiveness of M353 vs E 822, a more complete description of this pocket should appear somewhere in the text.
Figure 4
4c Where did compound E3 come from? It does not appear to be compared with the the two inhibitors that are the subject of the manuscript. Perhaps it would be helpful to provide IC50 for this compound against UT-A2 as a description for comparison? Apologies if it is given elsewhere in the manuscript and I missed it.
Figure 5
5c include y axis units
Figure 6
6a OK, although it is unclear in the methods section whether this is complete water restriction or a decreased, measured provision of water. This should be added to the methods section.
6b – 6c the axis is concentration units but the papilla is not a liquid. Some additional description is needed in the legend (ie….measured in papillary lysates from mice under…)
6c and 6e the y axes should read osmolality or osmolarity
6 d and 6 e The concentration of urine urea and the urine osmolality would be more informative if there was data on the volume of urine under these different experimental conditions. Is this data available?

Reviewer #2

(Remarks to the Author)
Authors present UT-A specific inhibitors developed with their pipeline of urea transporter drug discovery, and characterise the inhibition structurally and biophysically with cryo-EM and assays. Their inhibitor discovery methods, validation and findings are substantial, solid and well-presented. M353-0039, their main compound output from the work, will be a very useful chemical tool for studying UT-A2 function.

There are some major concerns, however most of these (if not all) can be addressed by updating text without additional experiments. The general concern is that the authors are overstating the novelty of their work.

1. While the work is substantial, the scientific advance is relatively narrow. The general workflow is very standard for a target-based drug discovery, so some of the statements (e.g line 46 – "lay a strategic foundation for…") may need to be toned down. Mildly selective UT-A selective inhibitors, some of which the authors use in this work, have been available for a while, and there are also specific (but less selective) inhibitors. The cryo-EM structures and urease-coupled assays are not novel either, given their more substantial Nat Comm (2024) paper. While the improved selectivity for UT-A2 (vs. UT-A3 and UT-B) of the presented inhibitors is noted, this is still an iterative work and should be presented as such, rather than overstating the advances made (e.g. lines 95-98, 306-312).

2. The similarity between HQA2 and E822-1968 is understated (line 133-136, figure 2f) - one half of them are identical, and the other half are structurally not that different either. The old approach could efficiently find a similar inhibitor series ten years ago, and this "new" strategy basically ended up reinventing the wheel. Wouldn't a conventional SBDD/SAR approach (which often involves "scaffold-hopping", line 312) have been a more efficient way of improving HQA2 to E822-1968-like molecules?

3. Authors' claim that targeting UT-A2 is superior to inhibiting other urea transporters is self-contradictory. In the introduction, the rationale for targeting UT-A2 is that its expression is largely limited to the kidney. However, they then go on to find UT-A2

expression in hepatic cells. Authors only make a passing mention of this in Discussions.

Minor points:
1. There are typos in the main figures (e.g. Figure 1a – "In vtrio" instead of "in vitro"; Figure 6a has “water deprvstion”), and in texts (e.g. line 515 - "ab initio" needs to be italicised; line 532 - use actual superscript instead of "^" sign). Please double-check.

2. The inhibitor for UT-B, UTBinh-14, has a 2-thienyl group that inserts into EUBP, which is similar to M353-0039. It will be worth adding a figure panel (main or supplementary) and some statements making this comparison (e.g. both molecules appear to interact with a cysteine residue given Figure 5h).

3. Figure 6g has a misleading y-axis – the intensity between M353 positive/negative samples is only ~15%. While the baseline does not need to be zero, this seems a bit extreme given the actual level of difference and the small number of replicates.

4. Also the data on Figure 6g – is this the best way to analyse the data? Wouldn’t it be better to measure cytoplasmic/supernatant urea levels of each sample, rather than measuring difference between samples for cytoplasm and supernatant separately? If anything, the other approach will show even more significance, so the authors’ method is a bit perplexing.

5. All figures are full-page panels, yet the texts and panels are often too small to read/see without zooming in. Authors should consider “decluttering” the figures for better legibility.

6. Have the authors tried measuring/calculating Hill coefficient?

7. EM sample preparation (line 497-505): What Falcon camera version is this? Also, data table and image processing workflow figures (in supplementary file) suggest that both datasets were collected with a Falcon camera – it is unusual for a K3 camera to have 0.92 A pixel size. Why is K3 mentioned? EPU software version needs to be stated. If “customized specific scripts” were used, a description of what these scripts are would be useful.

8. Cryo-EM image processing and structure refinement (line 507 onwards): please add the versions of the softwares used (and company name). Line 510 – “depending on the image processing software” – what does this mean? Also, line 511 – “Each micrograph was manually inspected to remove images contaminated by crystalline ice or other visible contaminants” – It is hard to believe that authors manually inspected every one of 6,746 images. Topaz needs its own citation.

9. Supplementary Table 1, and Supplementary Figures 2 and 4 – Please add Q-score for each dataset. Also, it is hard to believe that the local resolutions went all the way to 1.9 – 2.0 A (this is basically to the Nyquist limit) while the nominal resolutions are nearly an angstrom higher. Upon manual inspection of the map, I do not see any region that looks like 2.0 A-ish resolution. Authors will need to clarify what methods were used to calculate the range, and if possible, use stricter criteria as the numbers do not match the actual maps. Given the disparity, they should also detail this in the methods section.

10. Supplementary Figure 2 – specific to this dataset, GSFSC curve suggests presence of duplicated particles. Please try removing duplicate particles with spacing at least the size of the protein (Cryosparc has this function) then redo the reconstruction. If any particle drops out, the map/model will need to be redeposited and this figure set updated. (While all other figures will also need to be updated accordingly in theory, I will leave it to the editors to determine whether this is necessary – I expect the changes to be insignificant, and the updated ones will probably look almost identical to the current ones).

11. Targeting UT-B, despite its wide distribution, may not be as bad as the authors make it out to be in the introduction (line 84 – 90). There are already humans with SLC14A1 knockouts and knockdowns – Jk(null) and Jk(weak) blood group phenotypes. Apart from mild diuresis, which the authors are actually aiming for, health effects are yet to be reported for these individuals.

12. Regarding the observations with mouse studies (effect only in low-protein diet group), is there a possibility that the compound's effect on the liver is contributing to this?

13. Please check citations and add/update as necessary. For example, no citation is provided for HQA2. DoGsitescorer (which I believe the authors are referring to - last "r" is missing) needs citation as well.

Reviewer #3

(Remarks to the Author)
Huang et al. report the discovery and structural characterization of small-molecule inhibitors of the human urea transporter UT-A2. Through virtual screening and functional characterization, they identify two chemotypes (E822-1968 and M353-0039) that bind selectively to UT-A2 and solve cryo-EM co-structures at 2.9 Å and 2.7 Å resolution. M353-0039 inhibits UT-A2 with an $IC_{50} \approx 0.35$ µM and shows marked selectivity over UT-A1/A3 and UT-B. Structural analyses implicate non-conserved residues C285 and G322 in a “T–T” subpocket as the determinants of selectivity, confirmed by mutagenesis. In vivo administration in mice reduces renal medullary urea and osmolality, and HepG2 assays indicate cellular inhibition of

urea transport. The study provides a credible structural and functional basis for selective UT-A2 probes and a possible route toward next-generation diuretics.
The manuscript is technically sound and addresses an unmet pharmacological need, but several aspects of experimental rigor, nomenclature, and mechanistic interpretation should be revised before publication.
Major Comments
1. The lead compound alternates between M353-0039, M353-0059, and M353-0093 across figures and methods (e.g., Fig. 1, Methods §2.3, Extended Data Fig. 2). The authors must verify all identifiers and ensure uniform labelling throughout the text and supplementary materials.
2. Many panels employ unpaired two-tailed t-tests with n = 4–5. Please clarify whether n represents biological or technical replicates, whether randomization/blinding was applied, and provide justification or use ANOVA with multiple-comparison correction. Report animal sex, age, and inclusion/exclusion criteria.
3. The 80 mg $kg^{-1}$ i.p. dosing lacks formulation, PK/PD, and tolerability information. Please include vehicle composition, exposure data, and, if possible, plasma or kidney compound concentrations. A UT-A2-knockout control (or demonstration of no further effect) would confirm on-target activity.
4. Line 255: The term allosteric pocket implies non-competitive inhibition, yet no competition or urea-dependence analyses (i.e. Schild plot) are presented. Unless such pharmacological evidence is added, replace “allosteric pocket” with “extrachannel subpocket on the extracellular face” or similar, and explicitly note that competitive versus non-competitive mechanism has not been established.
5. Mean B-factors of 3 $Å^2$ for protein/ligand are implausibly low. Please confirm refinement parameters, describe map-sharpening settings, and provide map–model FSC curves. Ensure that the deposited PDB/EMD entries correspond to these maps and include validation statistics.
6. Functional assays use human UT-A2, while in vivo studies use mouse. Include cross-species sequence alignment to show conservation of interacting residues.
7. The predominant hepatic UT isoform in HepG2 is uncertain. Authors should at minimum present qPCR or Western blot data confirming UT-A2 expression in HepG2 cells.
8. No hERG, CYP, or cytotoxicity data are reported. At minimum, include a statement clarifying that these compounds serve as chemical probes rather than leads, and note any in-silico or panel screens used to flag potential liabilities.
9. The urease-coupled assay provides an indirect readout of urea flux. While the cryo-EM structures convincingly establish direct compound binding to UT-A2, an orthogonal functional assay (e.g., radiolabeled or LC–MS urea flux, or stopped-flow light scattering) would help confirm that this structural binding event indeed translates to transport inhibition rather than a non-conducting conformational lock.”

Minor Comments
• Unify abbreviations for binding pockets (ATCP, EUBP, EBBP, T–T). Provide a schematic in the Supplementary.
• Language and typos:
o Fig. 1 A: correct “in vitro” and “structural analysis.”
o Line 95: “our works” → “our work.”
o Line 97: “...as next-generation diuretics.”
o Line 255: replace “allosteric pocket” per above.
• Supplementary Fig. 5d (CCK-8): specify cell line, seeding density, exposure time, vehicle, n, and statistical test
• Supplementary Table 1: verify mean B-factors and include map-sharpening parameters.
• Minor style issues: e.g., “Data was” → “Data were,” “reach equilibrium state” → “reach equilibrium”

Reviewer #4

(Remarks to the Author)
This manuscript reports the discovery of novel selective urea transporter inhibitors through an integrated approach combining high-throughput screening, pharmacological validation, and structural characterization. While the overall strategy is sound and the results are highly interesting, several aspects require clarification and additional detail prior to publication:

Major Comments

1.) Custom database design (line ~113): The custom-designed database is insufficiently described in the main text and also the Methods section. Please provide detailed information on how this database was constructed, what criteria were used for compound selection, and what distinguishes it from the original database used.

2.)Docking validation: The screening workflow appears to lack validation. Was a ROC curve analysis or similar benchmarking performed to assess the docking protocol's performance? Validation against known actives and decoys would strengthen the computational predictions.

3.)Hit selection (line ~117): The manuscript identifies certain residues as pivotal for binding but then excludes molecules that interact with these regions. This seems contradictory—please clarify the reasoning for excluding molecules that engage with the defined binding pocket.

4.)Screening workflow (line ~179): The second screening workflow targeting the deeper pocket needs more detailed description.

5.)Comparison with experimental data: It would strengthen the manuscript to compare your docking predictions with

available cryo-EM data. How well do the predicted binding modes align with experimental structures?

Minor Comments

1.)SI Figure 1c: Please label more clearly that this is a sectional/cut view of the protein from the side. The orientation isn't immediately apparent.

3.)Line ~240 - Hydrophobic interactions terminology: When describing protein-ligand interactions, consider using "hydrophobic contacts" rather than focusing solely on van der Waals forces. The term "van der Waals forces" describes only the enthalpic component and disregards the entropic energy gains from releasing water from the binding pocket, which is actually the main driving force for these interactions.

4.)Line ~179 - "Filtration" terminology: The term "filtration" sounds imprecise in this context. Consider rephrasing to "we implemented a filtering strategy" or similar.

5.)Line ~405 - "Enhanced sampling": This term is typically used for enhanced sampling in molecular dynamics simulations (metadynamics, replica exchange, etc.). If you're not referring to these techniques, please use alternative phrasing to avoid confusion.

6.)Figure 4 - Chemical structures: The structure drawing of compound E762-1894 (and to a lesser degree the other compounds) does not adhere to standard conventions for chemical structure representation. Please redraw using software such as ChemDraw with proper bond angles, consistent formatting.

Version 1:

Reviewer comments:

Reviewer #1

(Remarks to the Author)
This is a data rich and extremely technical manuscript that identifies and provides evidence for an apparently specific inhibitor of the UT-A2 urea transporter. In response to the original review, the manuscript has been improved by adding some details, defining some acronyms, and enhancing the discussion. There are a couple of remaining small issues in the text that were apparent, but mostly all original comments have been addressed. Below is a list of the comment responses.
Responses to reviewer #1:
Line 87
Rephrase “urea concentration in renal” perhaps renal urea concentration or urine urea concentration in kidney
Intro revision OK
Results:
Comment 1. OK ;Comment 2. OK ; Comment 3. OK ; Comment 4. OK
Comment 5. No changes required.
Comment 6. Nice addition, OK ; Comment 7. OK
Discussion
Comment 8. Need to correct type-o’s or wording in some places, ie., physiological instead of physiologically and little vs litter. (lines 359 – 361)
Comment 9. Adequate although the response mentions that MDCK cells were not able to be effectively transfected with UT-A2, this was not apparently added to the text. This is not of sufficient importance to require additional experimentation.
Comment 10. Interesting addition and worth adding
Figures and Legends.
Comment 11. OK ; Comment 12. OK ; Comment 13. .1 E. OK ; 13.2 G. OK ; 13.3 OK
Comment 14. OK ; Comment 15. OK
Comment 16. 16.1 OK 16.2 Change ‘Papillary’ to ‘the papilla’ ; 16.3 OK ; 16.4 OK
Suggest double checking the wording on the figure legends.
Overall, nice job in responding to this reviewer’s queries.

Reviewer #2

(Remarks to the Author)
Authors have mostly addressed my concerns. They have also markedly expanded the manuscript with additional analysis and literature review. Only a few minor comments:

1. Discussion section is now much longer. If the journal format allows, it will be great to split it into subsections.

2. Several citations are still missing, particularly in the methods section. For example, revision has led to UCSF Chimera being mentioned earlier, but it is not cited there (line 677).

3. Some figures are still a bit difficult to read. For example, Fig. 3g has some residues in light brown (e.g. A326) which

makes them illegible. Similar with panels Fig. 4a and 5f.

Reviewer #3

(Remarks to the Author)
The points raised in the previous round of review were addressed and I have no further concerns regarding this manuscript.

Reviewer #4

(Remarks to the Author)
Almost all comments have been addressed sufficiently.

However, some minor comments still needs reworking:

1.) (2.) Docking validation): For visual clarity, please ensure that the ROC curve originates at the origin, i.e. remove the whitespace at the bottom and on the left, and move the 0s for x and y to the origin.

2.) (10. Figure 4 – Chemical Structures): When creating figures containing 2D depictions of molecules, please refer to the style guidelines (e.g. problematic bond angles in HQA2 and many other molecules) (https://www.nature.com/documents/nr-chemical-structures-guide.pdf). ChemDraw, for example, allows you to 'clean up' the structure.

**Open Access** This Peer Review File is licensed under a Creative Commons Attribution 4.0 International License, which permits use, sharing, adaptation, distribution and reproduction in any medium or format, as long as you give appropriate credit to the original author(s) and the source, provide a link to the Creative Commons license, and indicate if changes were made.
In cases where reviewers are anonymous, credit should be given to 'Anonymous Referee' and the source.
The images or other third party material in this Peer Review File are included in the article's Creative Commons license, unless indicated otherwise in a credit line to the material. If material is not included in the article's Creative Commons license and your intended use is not permitted by statutory regulation or exceeds the permitted use, you will need to obtain permission directly from the copyright holder.
To view a copy of this license, visit https://creativecommons.org/licenses/by/4.0/

**Responses to reviewers' comments**

We thank the reviewers for their valuable time in reviewing our manuscript and the helpful suggestions that they have provided. We have carefully taken these comments and suggestions from the reviewers into careful consideration in preparing our revised version, which has resulted in a more thorough and clear manuscript. Please find below a point-by-point response to the comments with our responses in ***Blue*** and the referees' comments in ***black***.

**REVIEWER COMMENTS**

Reviewer #1 (Remarks to the Author)

This is a data rich and extremely technical manuscript that identifies and provides evidence for an apparently specific inhibitor of the UT-A2 urea transporter. The methodology appears sound but the manuscript could be improved by adding some details, defining some acronyms, and enhancing the discussion.

**Reply:** We thank the reviewer for his/her positive comments.

Specific suggestions:

Introduction

In introduction, the authors disclose the relationship between UT-B and extrarenal pathology. They also say that UT-B shares about 40% sequence difference with UT-A2. They then suggest that inhibition the sequence similarities between UT-B and UT-A2 might represent an opportunity for inhibitors of UT-A2 to have off target extra-renal effects. This is offered as justification for developing inhibitors with high specificity for UT-A2.

**Reply:** We thank the reviewer for his/her valuable suggestion. We have revised the original text to clarify that "*Structural analysis reveals that UT-B exhibits approximately 40% sequence difference with UT-A2, suggesting that their sequence divergence likely underlies distinct functional specializations. Importantly, these differences offer an opportunity for developing selective inhibitors of UT-A2 to avoid the off target extra-renal effects from the non-selective*

*UT inhibitors that can block both UT-A and UT-B.*” at line 89-93.

Results

1. Line 112 is there a definition for the “SCG” pocket?

**Reply:** We thank the reviewer for his/her valuable suggestion. We have provided a detailed description of all the pockets involved in this study in Supplementary Table 1 (also see Table R1).

Table R1. The diverse pockets of urea transporter UT-A2 for inhibitor interaction.

| Pockets | Universal name | Description | Reference |
|---|---|---|---|
| EUBP | The extracellular urea binding pocket | This urea molecule binding pocket is located in the region of the UT-A2 urea transport channel near the extracellular side, primarily comprising the amino acids L131, F179, Q231, V232, F287, C337 and T338. | Nat Commun. 2024 Nov 26;15(1):10226. |
| EBBP | The extracellular blocker binding pocket | Located on the extracellular side of urea transport channel in the UT molecular, the primary binding region for inhibitors includes two sub-pockets, EBBP1 and EBBP2, primarily comprising the amino acids F135, L202, F282, L284, P335, and P336. | Nat Commun. 2024 Nov 26;15(1):10226. |
| SCGP | “SCG” pocket | This pocket is completely independent of the urea transport channel of UT-A2 and is composed of T275, S279, C285, G322 and A326 from UT-A2. The residues of the SCG pocket region are not conserved among different UTs, particularly at positions S279, C285 and G322, making it a potential target pocket for developing UT-selective inhibitors. | Nat Commun. 2024 Nov 26;15(1):10226. |
| ATCP | “ATC” pocket | Located on the extracellular side of UT-A2 and independent of both the urea transport channel and the EBBP region, this subpocket is enclosed by residues A270, I273, T275, C285, and A323. | This article |
| “T-T” pocket | The binding pocket of the thiophene-thiazole group | Located on the extracellular side of UT-A2 and independent of its urea transport channel, this region serves as the binding site for the thiophene-thiazole group of inhibitor M353-0039, involving residues A326, A319, C285, T275, A270, I273, G322, A323, L284 and P336. | This article |
| L-P pocket | L-P pocket | The region near L202 and P335 on the EBBP of UT-A2, where these two residues are not | Nat Commun. |

| | | | |
|---|---|---|---|
| | | conserved across different UT subtypes, represents a potential binding pocket for developing UT-selective inhibitors. | 2024 Nov 26;15(1):10226. |

2.Line 135 the initial mention of HQA2 requires a reference that provides the initial description.

**Reply:** We thank the reviewer for his/her valuable suggestion. We have provided the detailed descriptions and reference of all the small molecules involved in this study, and included their detailed information in Supplementary Table 2 (also see Table R2);

Table R2. Details of the molecules involved in developing UTA2-selective inhibitors.

| Molecule | Universal name | Structural formula | Description | Reference |
|---|---|---|---|---|
| E822; E822-1968; | 3-(3-methoxyphenyl)-N-[(3-methoxyphenyl)methyl]-1H-pyrazole-5-carboxamide | 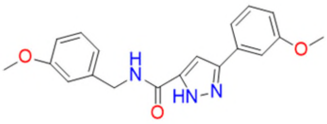 | This study reports E822-1968 with $IC_{50}$ values of 0.44 ± 0.03 µM for UT-A2 and 3.42 ± 0.35 µM for UT-A3, respectively. | This article |
| M353; M353-0039 | N-(2-{[2-methyl-5-(4-methyl-1,3-thiazol-2-yl)thiophen-3-yl]amino}-2-oxoethyl)furan-2-carboxamide | 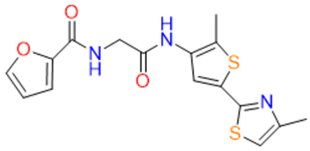 | This study reports M353-0039 with an $IC_{50}$ of 0.35 ± 0.03 µM against UT-A2. | This article |
| 25a | N-(4-acetamidophenyl)-5-acetylfuran-2-carboxamide | 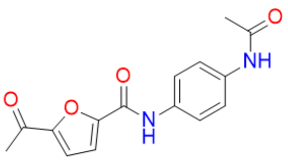 | This UT inhibitor is reported the $IC_{50}$ values of 0.14 µM for rat UT-B, 0.48 µM for mouse UT-B and 0.58 µM for human UT-A2. | Eur J Med Chem.15:226:113859 Nat Commun. 15(1):10226. |
| HQA2 | 8-hydroxy-N-(3-methoxybenzyl)quinoline-2-carboxamide | 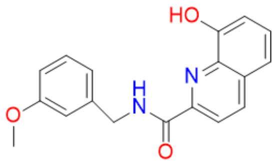 | Mildly selective UT-A selective inhibitors. This UT inhibitor is reported the $IC_{50}$ of 5.3 µM against UT-A1, 3.66 ± 0.09 µM against UT-A2 with less inhibition against | Chem Biol. 20(10):1235-44.; Nat Commun. 15(1):10226. |

| | | | | |
|---|---|---|---|---|
| | | | UT-B. | |
| E3 | 5-acetyl-N-[3-(phenylsulfonamido)phenyl]furan-2-carboxamide | 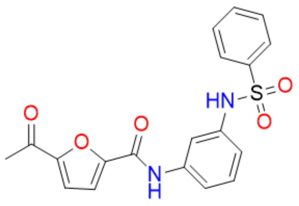 | This UT inhibitor is reported the $IC_{50}$ values of 0.22 ± 0.03 μM for UT-A2 and 0.52 ± 0.02 μM for UT-A3, respectively. | Acta Pharmacol Sin. 46(11):2989-2997.; Biomedicines. 13(4):992. |
| D400-1574 | N-{2-[3-(4-methoxyphenyl)-1,2,4-oxadiazol-5-yl]phenyl}-1-(2-methylphenyl)-5-oxopyrrolidine-3-carboxamide | 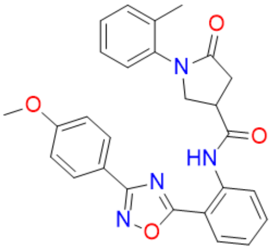 | This study reports D400-1574 with an $IC_{50}$ of 3.42 ± 0.96 μM against UT-A2. | This article |
| E762-1894 | 2-ethyl-N-[(5-methylfuran-2-yl)methyl]-2,3-dihydro[1,4]oxazino[2,3,4-hi]indole-5-carboxamide | 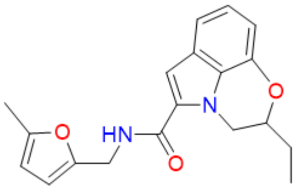 | The molecular shows no inhibitory effect on UTs. | This article |
| 8012-3374 | 2-[(5-amino-1,3,4-thiadiazol-2-yl)sulfanyl]-1-([1,1'-biphenyl]-4-yl)ethan-1-one | 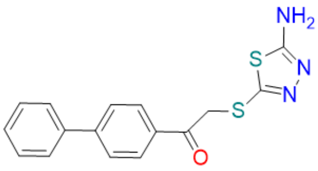 | The molecular shows no inhibitory effect on UTs. | This article |
| D103-1163 | 7-methyl-N-[2-(4-methylbenzoyl)-1-benzofuran-3-yl]-4-oxo-4H-1-benzopyran-2-carboxamide | 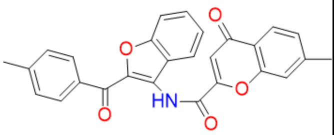 | The molecular shows no inhibitory effect on UTs. | This article |

3.Line 156 is confusing. “into an extended subpocket out of the ebbp…. Consider rewording.

Ie., into a subpocket contiguous with but outside of the EBBP and SCG pockets.

**Reply:** We thank the reviewer for his/her valuable suggestion. We have revised the manuscript according to your suggestion at line 162-163.

4.Line 159 "direct interactions with 16 amino acids of E822-1068 The inhibitor is not composed of amino acids so the wording makes understanding very difficult. Revise to say interactions between 16 amino acids in the UT-A2 pockets with E822-1068 …

**Reply:** We thank the reviewer for his/her valuable suggestion. We have revised the word to "*Sixteen residues of UT-A2 formed direct interactions with E822-1968, two more than the number interacting with HQA2*" at line 166-167.

5.The descriptions of the specific amino acid side chain bindings to the rings and R groups of the inhibitors are very detailed and informative, although they make a complicated read.

**Reply:** We thank the reviewer for his/her comment.

6.Line 264-267 After describing the UT-A2 knockout mice the description of the next experiment (low protein, water deprivation) needs to specify what mice are being treated to be compared to the UT-A2 mice. Insert "control" or "wild type' or similar to clarify.

**Reply:** We thank the reviewer for his/her valuable suggestion. In this study, we utilized wild-type mice administered the UT-A2 selective inhibitor M353-0039 to simulate the effects observed in UT-A2 knockout mice. The results demonstrated that M353-0039-treated wild-type mice exhibited responses comparable to those of UT-A2 knockout mice previously reported by Uchida, S. et al. (Mol Cell Biol. 25(16):7357-63). We have provided necessary supplementary information of the mice in the section "M353-0039 selectively inhibits UT-A2 *in vivo*". We have also included more descriptions as follows: "*Therefore, we administered an intraperitoneal injection of 80 mg/kg M353-0039 to validate the in vivo selective potency in wild-type mice with a low-protein diet and water deprivation to simulate the effects observed in UT-A2 knockout mice.*" at line 277-280.

7. Concerning the experiment with low protein diet +/- M353 cmpd. Clearly there is an effect of the inhibitor, but urine volume is not explained and would impact the interpretation of these

data. In a water deprivation study there would be drastic changes in urine volume and would vary depending on the protein load.

**Reply:** We thank the reviewer for his/her valuable suggestion. The animal study was designed as a validation experiment to assess the *in vivo* selective inhibition of UT-A2 by M353-0039. We have recorded the urine output of mice on a low-protein diet for 7 days (Figure R1, also Supplementary Fig. 5e). From day 7 onward, animals underwent water deprivation; during the following 24 h, urine volume was markedly reduced and its viscosity increased substantially. The urine collection via metabolic cages incurred substantial systematic error, making the measurements statistically unreliable. As a result, these measurements could not be reliably recorded. In line with the method of Uchida, S. et al. (Mol Cell Biol. 25(16):7357–63), we collected urine samples from bladder-retained urine for urea concentration and osmolality analyses. We have discussed these words at line 389-394 in the revised manuscript at follow: *"In order to verify the in vivo activity of M353-0039, we applied the same experimental conditions as those employed by Uchida, S. et al. to treat the mice, because the residues of the binding pocket of M353-0039 in human and mouse are completely identical. After water deprivation, urine volume fell sharply and viscosity rose, so metabolic cages failed to collect accurate volumes. We therefore collected bladder urine as Uchida, S.'s method to measure urea concentration and osmolality."*

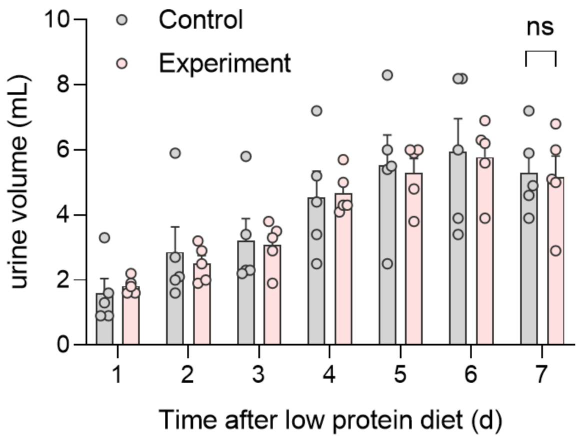


Figure R1. The urine volume of mice treated with low protein diet. ns, no significant; statistical differences were determined by the two-sided unpaired Student's t-test. Data were shown as the means ± SEM (n=5).

Diacussion

8. The discussion is limited. The first part essentially restates the key points in the results section without extending these results into a deeper discussion of the potential meaning of these results. For example, UT-A2 is essentially identical with one half of the UT-A1 molecule. A discussion of how the inhibitor fails to inhibit UT-A1 while successfully inhibiting UT-A2 could be explored.

**Reply:** We thank the reviewer for his/her valuable suggestion. Although UT-A1 shares identical amino acid sequence composition with the combined UT-A2 and UT-A3 domains, its quaternary structure in the cell membrane remains structurally uncharacterized. Gamma Chi et al. attempting to resolve UT-A1's structure exclusively identified a trimeric assembly derived from the N-terminal UT-A3 segment, with no discernible electron density corresponding to the C-terminal UT-A2 portion (Sci Adv. 2023 Sep 29;9(39): eadg8229). Concordantly, our prior work demonstrated that while UT-A2 and UT-A3 exhibit robust membrane localization upon heterologous overexpression, UT-A1 shows negligible membrane integration under identical conditions (Nat Commun. 2024 Nov 26;15(1):10226.). These findings suggest that the physiologically structure of UT-A on cell membrane is likely not a simple modular assembly of its constituent UT-A2 and UT-A3 subunits, which may lead to the functional phenomenon that inhibitors selective for UT-A2, like compound M353-0039, exhibit litter efficacy against UT-A1. Therefore, further investigation in the future on UT-A1 are need to underlying the mechanisms. We have expanded the discussion at line 349-362 as follow:

*"As the first reported UT-A2 selective inhibitor, we unexpectedly observed that M353-0039 lacks appreciable activity against UT-A1, although UT-A2 is essentially identical with one half of the UT-A1 molecule. UT-A1 shares identical amino acid sequence composition with the combined UT-A2 and UT-A3 domains, but the quaternary structure of UT-A1 remains structurally uncharacterized. Gamma Chi et al. attempting to resolve UT-A1's structure exclusively identified a trimeric assembly derived from the N-terminal UT-A3 segment, with no discernible electron density corresponding to the C-terminal UT-A2 portion. Concordantly, our prior work demonstrated that while UT-A2 and UT-A3 exhibit robust membrane localization upon heterologous overexpression, UT-A1 shows negligible membrane integration under*

*identical conditions. These findings suggest that the physiologically structure of UT-A on cell membrane is likely not a simple modular assembly of its constituent UT-A2 and UT-A3 subunits, which may lead to the functional phenomenon that inhibitors selective for UT-A2, like compound M353-0039, exhibit litter efficacy against UT-A1."*

In addition, we have also expanded the discussion to include details about the virtual screening methodology, in vivo experiments and so on in the revised manuscript.

9. The potential for the MDCK testing medium for UT-A1 inhibition to provide a potential false negative. What happens if you put UT-A2 in MDCK cells and repeat the inhibition protocol? Would this provide more rigor to the data.

**Reply:** We thank the reviewer for his/her valuable suggestion.

(I) To eliminate the potential for false-negative results in the MDCK-based assays, we routinely monitored the transepithelial electrical resistance (TEER) of the MDCK cell monolayer before performing inhibition assays. Inhibition experiments were only conducted when the TEER value consistently reached above 1 $k\Omega \cdot cm^2$, ensuring the formation of a compact, intact, and leak-free cell monolayer. In addition, we included a negative control group (Vehicle) and a positive control group treated with Compound 25a to validate the reliability and feasibility of the experimental system. The method of using MDCK cells stably expressing UT-A1 to investigate the effects of inhibitor molecules on UT-A1 has been employed in multiple studies *(Am J Physiol Cell Physiol. 2004 Jun;286(6):C1264-70; Am J Physiol Renal Physiol. 2008 Mar;294(3):F518-24; Acta Pharm Sin B. 2021 Jan;11(1):181-202)*. The experiments in this study were conducted following the methodologies described in these references. We have included these details in the Methods section of the revised manuscript at line 573-579.

(II) The MDCK cell stably overexpressing UT-A1 used in this study was established by Professor Yang's group many years through multiple rounds of screening. We also attempted to overexpress the UT-A2 protein in MDCK cells; however, despite extensive efforts, the expression level of UT-A2 in MDCK cells remained very low to reach the threshold required for reliable experimental assays. This limitation is likely attributable to the inherently poor transfection efficiency of nucleic acids in MDCK cells. Consequently, we were unable to assess

the response of the inhibitors on UT-A2 in the MDCK system. For this reason, we developed an alternative 293F cell-based system, as shown in Figure 2a, to characterize the inhibitory activity of the compounds on UT-A2.

10. In the discussion of in vivo effects of the inhibitor, the authors again just restate the results observed without exploring the potential explanation for the observations. Why is there an apparent difference between the normal protein load and the low protein load in the observed effect of the inhibitor? Also, explain why the animals were water deprived?

**Reply:** We thank the reviewer for his/her valuable suggestion. The focus of this study is the strategy for screening selective inhibitors of UT-A2. We discovered that compound M353-0039 exhibits selective inhibitory activity against UT-A2 in *vitro*, and therefore conducted mouse experiments to verify whether it also exerts selective effects *in vivo*. To investigate the role of UT-A2 in the inner renal medulla with respect to urea handling and its impact on urine-concentrating capacity, Shinichi Uchida et al. used gene-targeting technology to selectively disrupt UT-A2 expression without affecting the expression of other renal urea transporters. Unexpectedly, compared with UT-A1/A3-knockout and UT-B-knockout mice, UT-A2-knockout mice displayed only a mild defect in urine-concentrating ability, and this phenotype was observed only under stress conditions (low-protein diet combined with dehydration). These results indicate that UT-A2 function is "protein-diet dependent". Uchida S. et al. suggested that the functional importance of UT-A2 may be more pronounced in wild animals with inadequate daily protein intake than in laboratory animals, whereas the standard laboratory animal housing conditions might mask UT-A2's physiological significance (Mol Cell Biol.25(16):7357–7363). Consequently, under normal dietary conditions, the effect of UT-A2 inhibitor is not evident. Under low-protein-plus-dehydration conditions, however, the critical role of UT-A2 in maintaining a high urea concentration gradient in the inner medulla becomes apparent, thereby helping us to evaluate the selective inhibitory effect of M353-0039 *in vivo*. We have added this description to the Discussion section in the revised manuscript at line 376-390 as follow:

*"Shinichi Uchida et al. had used gene-targeting technology to selectively disrupt UT-A2 expression without affecting the expression of other renal urea transporters. Unexpectedly,*

*compared with UT-A1/A3-knockout and UT-B-knockout mice, UT-A2-knockout mice displayed only a mild defect in urine-concentrating ability, and this phenotype was observed only under stress conditions (low-protein diet combined with dehydration). These results indicate that UT-A2 function is "protein-diet dependent". They suggested that the functional importance of UT-A2 may be more pronounced in wild animals with inadequate daily protein intake than in laboratory animals, whereas the standard laboratory animal housing conditions might mask UT-A2's physiological significance. Consequently, under normal dietary conditions, the effect of UT-A2 inhibitor is not evident. Under low-protein-plus-dehydration conditions, however, the critical role of UT-A2 in maintaining a high urea concentration gradient in the inner medulla becomes apparent, thereby helping us to evaluate the selective inhibitory effect of M353-0039 in vivo. In order to verify the in vivo activity of M353 0039, we applied the same experimental conditions as those employed by Uchida, S. et al. to treat the mice."*

Figures & Legends

11.Figure 1

Multiple spelling errors in Figure 1a

11.1. 1b, refine the English (ie., Finally, 1 hit was identified to have UT-A2 inhibitory activity) Again, define the phrase "SCG" pocket.

**Reply:** We thank the reviewer for his/her valuable suggestion. We have revised the legend as follows: "*Approximately one million compounds were used for screening; the top 1000 (by docking score) were clustered, then 30 compounds were selected manually according to compound-SCG pocket interaction profiles. These molecules were further evaluated for their UT-A2 inhibitory activity through in vitro tests. Among them, Compound E822-1968 was confirmed to inhibit UT-A2 activity*" at line 883-887. Additionally, a detailed description of the "SCG" pocket has been added to Supplementary Table 1 (also see Table R1).

11.2. 1c, Just to be sure, the 30 compounds were selected based on their binding to the UT-A2 target (this is the 'selective filter' used to choose these potential inhibitors? And with all 30 showing binding capacity, only 1 compound show ANY inhibitory activity? Either the heatmap is an inadequate way to show the background inhibition by these other compounds, or it calls into question the selection methods. This one out of the 30 binding protein result should be

addressed/explained in the discussion.

**Reply:** We thank the reviewer for his/her valuable suggestion. In the first round of virtual screening, we utilized a relatively broad definition for the hotspot pocket. While this approach facilitated the identification of diverse binding modes, it also reduced the enrichment efficiency for functionally blocking inhibitors, because the effective inhibition of urea transporters depends not only on binding affinity, but also requires the inhibitor to physically occlude the urea permeation pathway. Consequently, from 30 candidate compounds tested, only one demonstrated measurable inhibitory activity. Based on this experience, the second-round screening focused on a refined hotspot pocket targeting the ATCP/EUBP sub-pocket, which improved the hit rate and ultimately led to the successful identification of a novel scaffold inhibitor, M353-0039, with enhanced selectivity. We have expanded these discussions in the revised manuscript at line 333-341 as "*In the first round of virtual screening, we utilized a relatively broad definition for the hotspot pocket. While this approach facilitated the identification of diverse binding modes, it also reduced the enrichment efficiency for functionally blocking inhibitors, because the effective inhibitions of urea transporters depend not only on binding affinity, but also requires the inhibitor to physically occlude the urea permeation pathway. Consequently, from 30 candidate compounds tested, only one demonstrated measurable inhibitory activity. Based on this experience, the second-round screening focused on a refined hotspot pocket targeting the ATCP/EUBP sub-pocket, which improved the hit rate and ultimately led to the successful identification of a novel scaffold inhibitor, M353-0039, with enhanced selectivity*".

11.3. 1d. nice clear information

**Reply:** We thank the reviewer for his/her positive comments

12. Figure2

12.1. 2a change centrifugal to centrifugation. Identify the type of cells that are being studied. 2b Line 672, type-0 IC5 should be IC50

**Reply:** We thank the reviewer for his/her suggestion. We have made the correction to the revised manuscript.

12.2. 2c the color for hUT-A3 is too light. Please change to something darker.

**Reply:** We thank the reviewer for his/her suggestion. We have changed the color of hUT-A3 to deep brown in the revised manuscript.

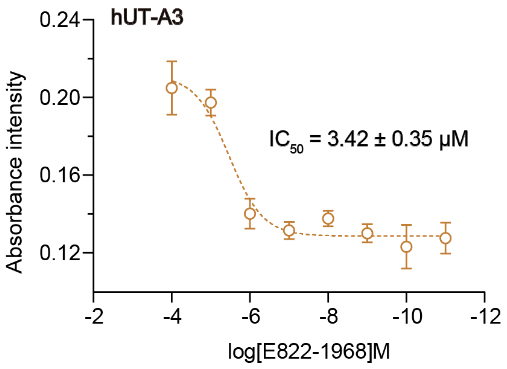


Figure R2 The inhibitory effects of E822-1968 to human UT-A3 validated by the urease reaction assay. E822-1968 showed inhibitory potency toward UT-A3 with the 3.42 ± 0.35 μM. Each data was derived from three independent experiments (n=3) to determine the $IC_{50}$ values. Data are shown as the means ± SEM.

12.3. 2e the positive control "25a" is not described either in the legend or the methods section. It needs to be added to the methods and properly referenced.

**Reply:** We thank the reviewer for his/her suggestion. We have added a detailed description of the compound 25a in the Methods section at line 600 and inserted the corresponding references. We have also included detailed information on 25a in Supplementary Table 2 (also see Table R2).

12.4. 2f if cmpd E822 was synthesized from HQA2, then fine. If not, remove the green arrow between the compound structures.

**Reply:** We thank the reviewer for his/her suggestion. Although E822 and HQA2 exhibit structural similarities, E822 is not synthesized from HQA2. We have removed the green arrow in the figure.

12.5. 2g Most of this data is from previous published work. Was UT-A1 actually measured in this work? Since it shows that it was not measured with the E822 compound but was not detected with the M353 compound. If the assay was performed with the M353 compound, why

are the results that were positive (UT-A2) not shown?

**Reply:** We thank the reviewer for his/her valuable suggestion. We initially used the system shown in Figure 2a to evaluate the inhibitory activity of the computationally screened small molecules against UT-A2, UT-A3 and UT-B. However, since UT-A1 transiently transfected in this system did not efficiently localize to the cell membrane, this approach was not suitable for assessing the effects of these compounds on UT-A1.The goal of this study was to develop selective inhibitors targeting UT-A2. During the initial screening, the E822 compound exhibited activity against both UT-A2 and UT-A3, indicating that it was not a selective UT-A2 inhibitor. Therefore, we did not pursue further investigation into its activity on UT-A1 in the previous manuscript version. Following your suggestion, we additionally evaluated the activity of E822 against UT-A1 by the transwell assay in the revised manuscript. As shown in Figure R3 (also see Figure 2f), E822 did not effectively inhibit UT-A1. We also included the testing results of the M353 compound against UT-A1 in Figure 4i, which similarly showed no significant inhibitory effect. We have discussed the possible reason of this phenomenon in the revised manuscript at line 349-362 (also see our reply to **Question 8**).

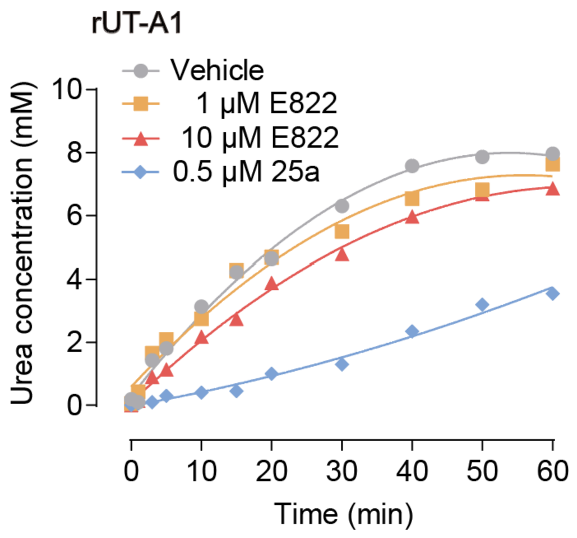


Figure R3. E822-1968 exhibited no significant inhibitory effects on rat UT-A1 stably expressing on MDCK cells in transwell assay, as compared to the positive control (compound 25a).

13. Figure 3

13.1. 3e. This reviewer is not an expert in special analysis of molecules so these images are difficult to interpret, however, the legend text might include a color description of the two compounds that are being compared (ie., HQA2 is drawn in turquoise while E822 is rendered in gold) to help the novice reader.

**Reply:** We thank the reviewer for his/her valuable suggestion. We have added detailed color descriptions to the legend of Figure 3e, and we have also included specific descriptions in other similar figures to make them easier for readers to interpret.

13.2. 3g Again, EBBP is defined, but SCG and ATC are not. Be sure they are defined earlier in the manuscript as they appear to be crucial to the mechanism of inhibition that is the object of this manuscript. Also, strongly suggest that the "SCG pocket" be colored something other than green as it disappears against the overall green background of the protein structure.

**Reply:** We thank the reviewer for his/her valuable suggestion. We have added detailed descriptions of all binding pockets to the Supplementary Table 1 (also see Table R1). We have also modified the color of SCGP to brown so that it can be clearly distinguished from the green background.

13.3. In addition, the T-T pocket is not described or defined other than "another binding pocket" but since it appears to be important in distinguishing effectiveness of M353 vs E822, a more complete description of this pocket should appear somewhere in the text.

**Reply:** We thank the reviewer for his/her valuable suggestion. We have added detailed descriptions of T-T pocket to the Supplementary Table 1 (also see Table R1).

14. Figure 4

4c Where did compound E3 come from? It does not appear to be compared with the two inhibitors that are the subject of the manuscript. Perhaps it would be helpful to provide IC50 for this compound against UT-A2 as a description for comparison? Apologies if it is given elsewhere in the manuscript and I missed it.

**Reply:** We thank the reviewer for his/her valuable suggestion. E3 is an orally active small-molecule UT inhibitor that we recently reported (Acta Pharmacol Sin. 2025 Nov;46(11):2989-

2997), with the $IC_{50}$ values of 0.22 ± 0.03 μM for UT-A2 and 0.52 ± 0.02 μM for UT-A3, respectively. We have provided the detailed descriptions and reference of E3 molecule in Supplementary Table 2 (also see Table R2)

15. Figure 5

5c include y axis units

**Reply:** We thank the reviewer for his/her valuable suggestion. We have added the unit ångström (Å) to the y-axis.

16. Figure 6

16.1. 6a OK, although it is unclear in the methods section whether this is complete water restriction or a decreased, measured provision of water. This should be added to the methods section.

**Reply:** We thank the reviewer for his/her valuable suggestion. To better evaluate the role of UT-A2 selective inhibitor, we followed the procedure in the study of Uchida, S. et al (Mol Cell Biol. 25(16):7357-63) using low-protein diet and water deprivation. In details, after seven days of sufficient water supply, the mice were not provided with water completely. We have revised the method as follows: "*Mice were subjected to complete water deprivation while being provided with sufficient food, and the diet type remained unchanged*" at line 708-710.

16.2. 6b – 6c the axis is concentration units but the papilla is not a liquid. Some additional description is needed in the legend (ie….measured in papillary lysates from mice under…)

**Reply:** We thank the reviewer for his/her valuable suggestion. According to the description in method, we have revised figure legend as follows at line 1006-1008: "*The urea concentration and osmolarity of papillary were measured in the supernatant of renal medulla homogenate supplied with 10 volume double-distilled water.*"

16.3. 6c and 6e the y axes should read osmolality or osmolarity

**Reply:** We thank the reviewer for his/her valuable suggestion. We have changed the unit to osmolarity in the y axis.

16.4. 6 d and 6 e The concentration of urine urea and the urine osmolality would be more informative if there was data on the volume of urine under these different experimental conditions. Is this data available?

**Reply:** We thank the reviewer for his/her valuable suggestion. The animal study was designed as a validation experiment to assess the *in vivo* selective inhibition of UT-A2 by M353-0039. We have recorded the urine output of mice on a low-protein diet for 7 days (see Figure R1 in Question 7). From day 7 onward, animals underwent water deprivation; during the following 24 h, urine volume was markedly reduced and its viscosity increased substantially. The urine collection via metabolic cages incurred substantial systematic error, making the measurements statistically unreliable. Consequently, these parameters were not recorded. Urine samples for urea concentration and osmolality analyses were finally taken from bladder retention. We have revised these words in line 392-394 at Discussion as follows: "*After water deprivation, urine volume fell sharply and viscosity rose, so metabolic cages failed to collect accurate volumes. We therefore collected bladder urine as Uchida, S.'s method to measure urea concentration and osmolality.*"

Reviewer #2 (Remarks to the Author)

Authors present UT-A specific inhibitors developed with their pipeline of urea transporter drug discovery, and characterise the inhibition structurally and biophysically with cryo-EM and assays. Their inhibitor discovery methods, validation and findings are substantial, solid and well-presented. M353-0039, their main compound output from the work, will be a very useful chemical tool for studying UT-A2 function.

**Reply:** We thank the reviewer for his/her positive comments.

There are some major concerns, however most of these (if not all) can be addressed by updating text without additional experiments. The general concern is that the authors are overstating the novelty of their work.

**Reply:** We thank the reviewer for his/her valuable suggestions. We have meticulously refined the manuscript according to these suggestions.

1. While the work is substantial, the scientific advance is relatively narrow. The general workflow is very standard for a target-based drug discovery, so some of the statements (e.g line 46 – “lay a strategic foundation for…”) may need to be toned down.

**Reply:** We thank the reviewer for his/her valuable suggestions. Our study primarily provides an applicable methodology for developing UT-A selective inhibitors. We have toned down relevant descriptions throughout the manuscript in line 49-51.

2. Mildly selective UT-A selective inhibitors, some of which the authors use in this work, have been available for a while, and there are also specific (but less selective) inhibitors.

**Reply:** We thank the reviewer for his/her valuable comments. HQA2 is a mildly selective UT-A selective inhibitor. We have added corresponding descriptions of these molecules in the Supplementary Table 2 (also see Table R3) of the revised manuscript.

Table R3. Details of the molecules involved in developing UTA2-selective inhibitors.

| Molecule | Universal name | Structural formula | Description | Reference |
|---|---|---|---|---|
| E822; E822-1968; | 3-(3-methoxyphenyl)-N-[(3-methoxyphenyl)methyl]-1H-pyrazole-5-carboxamide | 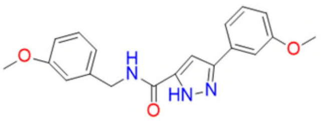 | This study reports E822-1968 with $IC_{50}$ values of 0.44 ± 0.03 µM for UT-A2 and 3.42 ± 0.35 µM for UT-A3, respectively. | This article |
| M353; M353-0039 | N-(2-{[2-methyl-5-(4-methyl-1,3-thiazol-2-yl)thiophen-3-yl]amino}-2-oxoethyl)furan-2-carboxamide | 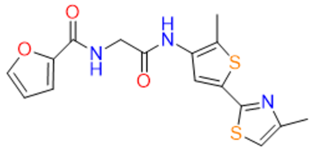 | This study reports M353-0039 with an $IC_{50}$ of 0.35 ± 0.03 µM against UT-A2. | This article |
| 25a | N-(4-acetamidophenyl)-5-acetylfuran-2-carboxamide | 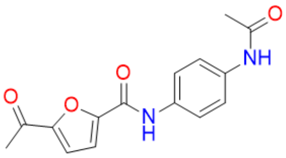 | This UT inhibitor is reported the $IC_{50}$ values of 0.14 µM for rat UT-B, 0.48 µM for mouse UT-B and 0.58 µM for human UT-A2. | Eur J Med Chem.15:226:113859 Nat Commun. 15(1):10226. |

| | | | | |
|---|---|---|---|---|
| HQA2 | 8-hydroxy-N-(3-methoxybenzyl)quinoline-2-carboxamide | 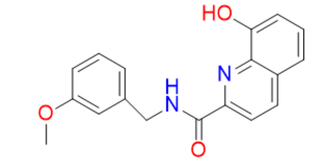 | Mildly selective UT-A selective inhibitors. This UT inhibitor is reported the $IC_{50}$ of 5.3 μM against UT-A1, 3.66 ± 0.09 μM against UT-A2 with less inhibition against UT-B. | Chem Biol. 20(10):1235-44.; Nat Commun. 15(1):10226. |
| E3 | 5-acetyl-N-[3-(phenylsulfonamido)phenyl]furan-2-carboxamide | 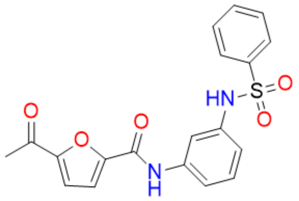 | This UT inhibitor is reported the $IC_{50}$ values of 0.22 ± 0.03 μM for UT-A2 and 0.52 ± 0.02 μM for UT-A3, respectively. | Acta Pharmacol Sin. 46(11):2989-2997.; Biomedicines. 13(4):992. |
| D400-1574 | N-{2-[3-(4-methoxyphenyl)-1,2,4-oxadiazol-5-yl]phenyl}-1-(2-methylphenyl)-5-oxopyrrolidine-3-carboxamide | 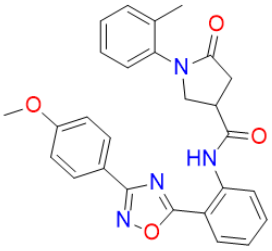 | This study reports D400-1574 with an $IC_{50}$ of 3.42 ± 0.96 μM against UT-A2. | This article |
| E762-1894 | 2-ethyl-N-[(5-methylfuran-2-yl)methyl]-2,3-dihydro[1,4]oxazino[2,3,4-hi]indole-5-carboxamide | 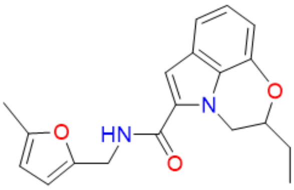 | The molecular shows no inhibitory effect on UTs. | This article |
| 8012-3374 | 2-[(5-amino-1,3,4-thiadiazol-2-yl)sulfanyl]-1-([1,1'-biphenyl]-4-yl)ethan-1-one | 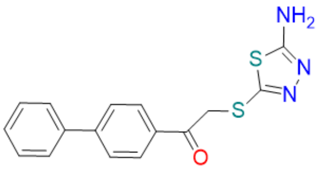 | The molecular shows no inhibitory effect on UTs. | This article |
| D103-1163 | 7-methyl-N-[2-(4-methylbenzoyl)-1-benzofuran-3-yl]-4-oxo-4H-1-benzopyran-2- | 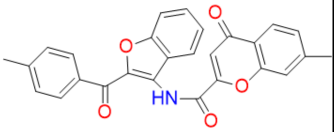 | The molecular shows no inhibitory effect on UTs. | This article |

| | | | | |
|---|---|---|---|---|
| | carboxamide | | | |

3. The cryo-EM structures and urease-coupled assays are not novel either, given their more substantial Nat Comm (2024) paper. While the improved selectivity for UT-A2 (vs. UT-A3 and UT-B) of the presented inhibitors is noted, this is still an iterative work and should be presented as such, rather than overstating the advances made (e.g. lines 95-98, 306-312).

**Reply:** We thank the reviewer for his/her valuable suggestions. As you noted, this manuscript represents an iterative extension of our paper on *Nature Communications* (2024). The advance of this study is developing an applicable methodology to find UT-A2 selective inhibitors. We have toned down relevant descriptions throughout the manuscript in line 102 and in line 328.

4. The similarity between HQA2 and E822-1968 is understated (line 133-136, figure 2f) - one half of them are identical, and the other half are structurally not that different either. The old approach could efficiently find a similar inhibitor series ten years ago, and this "new" strategy basically ended up reinventing the wheel. Wouldn't a conventional SBDD/SAR approach (which often involves "scaffold-hopping", line 312) have been a more efficient way of improving HQA2 to E822-1968-like molecules?

**Reply:** We thank the reviewer for his/her valuable suggestions. After identifying compound E822-1968, we then recognized its scaffold similarity to HQA2. This project was co-supervised by Professor Bao-Xue Yang, who has extensive research experience in developing UT inhibitors. Prior to obtaining UT-A/UT-B structural information, Professor Yang's team primarily employed scaffold-hopping strategies to develop inhibitor candidates. While this approach proved viable, it inherently carried high randomness, making it difficult to predict beneficial chemical modifications or achieve subtype selectivity. Furthermore, the synthesis and profiling of each new scaffold demanded considerable resources compared to standard library screening. Therefore, it is certainly viable to apply traditional SBDD/SAR methodologies to optimize HQA2 toward E822-1968-like molecules; however, this path would probably entail significantly increased costs. Consequently, upon acquiring UT-A/UT-B structural data, we began developing more efficient strategies for designing UT-selective inhibitors, as shown by this manuscript. We have added relevant discussion at line 341-347 of the manuscript as follow: "*By coincidence, in the first-round screening, we discovered an*

*inhibitor, E822-1968, that exhibits partial structural similarity to HQA2 in their molecular scaffolds, notably a shared methoxyphenyl group. This finding implies that conventional structure-based drug design (SBDD) and structure–activity relationship (SAR) approaches may guide optimization of HQA2 toward E822 1968 type molecules. Nevertheless, such a strategy is unlikely to afford M353 0039, owing to its completely distinct molecular framework.*"

5. Authors' claim that targeting UT-A2 is superior to inhibiting other urea transporters is self-contradictory. In the introduction, the rationale for targeting UT-A2 is that its expression is largely limited to the kidney. However, they then go on to find UT-A2 expression in hepatic cells. Authors only make a passing mention of this in Discussions.

**Reply:** We thank the reviewer for his/her valuable suggestions. UT-A2 is predominantly expressed and functions in the kidney. Klein JD et al. have suggested the possible presence of a UT-A2 isoform protein in hepatocytes, but this has not been conclusively confirmed (J Am Soc Nephrol 10, 2076-2083). Therefore, we performed cell-based experiments and found that hepatocytes do indeed express UT-A2 protein as M353 is an UT-A2 selective inhibitor. As to whether hepatocytes express other types of UT protein isoforms, and to what extent UT-A2 contributes to hepatic urea transport, our current data cannot draw definitive conclusions; future studies will be required to address these questions. We have added this discussion in the Discussion section at line 400-406 as *"Klein JD et al. have suggested the possible presence of a UT-A2 isoform protein in hepatocytes, but this has not been conclusively confirmed. Here, we performed cell-based experiments and found that hepatocytes do indeed express UT-A2 protein as M353 is an UT-A2 selective inhibitor, which was further confirmed by qPCR. However, as to whether hepatocytes express other types of UT protein isoforms, and to what extent UT-A2 contributes to hepatic urea transport, future studies will be required.*"

Minor points:

6. There are typos in the main figures (e.g. Figure 1a – "In vtrio" instead of "in vitro"; Figure 6a has "water deprvstion"), and in texts (e.g. line 515 - "ab initio" needs to be italicised; line 532 - use actual superscript instead of "^" sign). Please double-check.

**Reply:** We thank the reviewer for his/her valuable suggestions. We have corrected these errors and conducted a thorough review and revision of the entire manuscript in Figure1a, in Figure6a, in line 671 and in line 692.

7. The inhibitor for UT-B, UTBinh-14, has a 2-thienyl group that inserts into EUBP, which is similar to M353-0039. It will be worth adding a figure panel (main or supplementary) and some statements making this comparison (e.g. both molecules appear to interact with a cysteine residue given Figure 5h).

**Reply:** We thank the reviewer for his/her valuable suggestions. A new figure (Supplementary Figure 5d, also see Figure R4) has been included to show the binding mode of UTBinh-14 to the EUBP, with comparative analysis against M353-0039. Sequence alignment showed that the residue equivalent to C285 of UT-A2 in UT-B is W286, and thus UTBinh-14 failed to establish an analogous hydrogen bond with this tryptophan. Relevant explanatory text has been added to the revised manuscript at line 255-257.

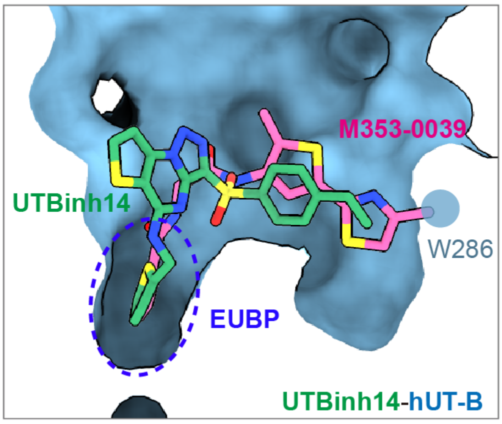


Figure R4. Comparison between UTBinh14 (green sticks) and M353-0039 (magenta sticks) in the ligand binding pocket in UTBinh14-UT-B complex (gray blue). The 2-thienyl group of UTBinh14 inserts into EUBP (blue-dashed line circle), which is similar to M353-0039, but fails to form hydrogen bond with the tryptophan on position 286 (W286, gray blue dot).

8. Figure 6g has a misleading y-axis – the intensity between M353 positive/negative samples is only ~15%. While the baseline does not need to be zero, this seems a bit extreme given the actual level of difference and the small number of replicates.

**Reply:** We thank the reviewer for his/her valuable suggestions. We have increased the number of experimental replicates, and as shown in the Figure R5 (also Figure 6g-h), the results indeed confirm that the proportion of M353-0039 positive/negative samples is only approximately 10-15%. We speculate that the possible reason may be related to UT-A2: although it is expressed

in HepG2 cells, its contribution to transmembrane urea transport in these cells is relatively low. Therefore, individually blocking UT-A2 has a limited effect on hepatic urea transport. We have added corresponding descriptions at line 400-406 in the Discussion section, also see the response provided for Question 5.

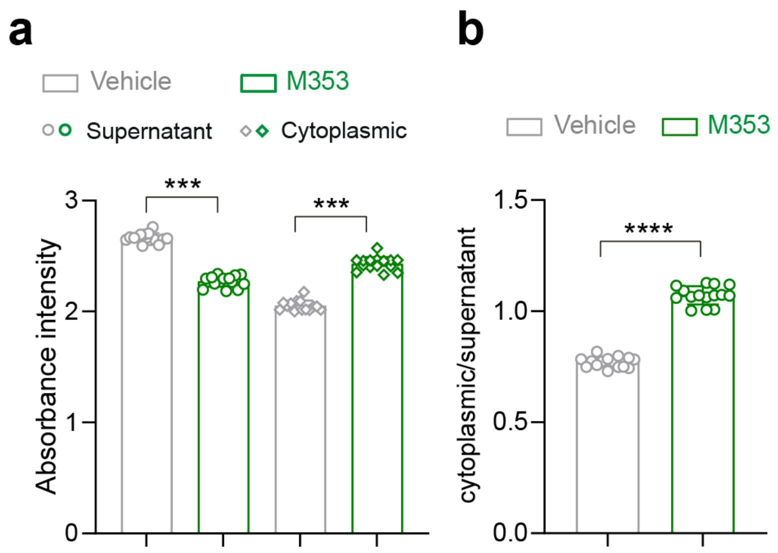


Figure R5. Effect of M353-0039 on urea transport in HepG2 cells. The data showed the urea concentration in the supernatant and cytoplasmic compartments (a), as well as in a cytoplasmic/supernatant format (b). The M353-0039 could inhibit urea efflux in HepG2 cells. ***$P < 0.001$, ****$P < 0.0001$; statistical differences were determined by the two-sided unpaired Student's t-test. Data were derived from fifteen independent experiments (n=15) and shown as the means ± SEM.

9. Also the data on Figure 6g – is this the best way to analyse the data? Wouldn't it be better to measure cytoplasmic/supernatant urea levels of each sample, rather than measuring difference between samples for cytoplasm and supernatant separately? If anything, the other approach will show even more significance, so the authors' method is a bit perplexing.

**Reply:** We thank the reviewer for his/her valuable suggestions. We have revised the contents of Fig 6g-6h to present the results in a cytoplasmic/supernatant format, as shown on Figure R5b in the response for Question 8.

10. All figures are full-page panels, yet the texts and panels are often too small to read/see without zooming in. Authors should consider "decluttering" the figures for better legibility.

**Reply:** We thank the reviewer for his/her valuable suggestions. We have adjusted the size of elements and fonts in the figures, and modified certain charts to improve readability.

11. Have the authors tried measuring/calculating Hill coefficient?

**Reply:** We thank the reviewer for his/her valuable suggestions. Since the effect of different inhibition mechanisms (competitive versus non-competitive) on inhibitory potency remains unclear, we did not pursue further investigation of the inhibitory mechanism of M353-0039, nor did we determine its Hill coefficient.

12. EM sample preparation (line 497-505): What Falcon camera version is this? Also, data table and image processing workflow figures (in supplementary file) suggest that both datasets were collected with a Falcon camera – it is unusual for a K3 camera to have 0.92 A pixel size. Why is K3 mentioned? EPU software version needs to be stated. If "customized specific scripts" were used, a description of what these scripts are would be useful.

**Reply:** We thank the reviewer for his/her valuable suggestions. We collected data using both the Gatan K3 camera and the Falcon counting camera. The structure with the current resolution was determined from data acquired on the Falcon camera. The "customized specific scripts" refer to the acquisition parameters we previously used for collecting data on the UT complex, primarily including magnification, defocus range, and electron dose settings, which are detailed in the Methods section. We have corrected these descriptions and supplemented the versions of the Falcon camera (Thermo Fisher Krios G4 Falcon 4i) and the EPU software (version 3.7, Thermo Fisher) in the revised manuscript at line 653-656.

13. Cryo-EM image processing and structure refinement (line 507 onwards): please add the versions of the softwares used (and company name). Line 510 – "depending on the image processing software" – what does this mean? Also, line 511 – "Each micrograph was manually inspected to remove images contaminated by crystalline ice or other visible contaminants" – It is hard to believe that authors manually inspected every one of 6,746 images. Topaz needs its own citation.

**Reply:** We thank the reviewer for his/her valuable suggestions. We have added the version number of the CryoSPARC software and the other software used in our study. We applied

the patch motion correction and patch CTF estimation in CryoSPARC to preprocess the micrographs. Then, we selected micrographs with an average defocus value between 8,000 and 20,000, which were further inspected to remove images contaminated by crystalline ice or other visible contaminants. Although this preprocessing step introduced a relatively large amount of manual work in the early stage, it significantly reduced the computational burden in later stages and ensured the overall data quality to facilitate high-resolution structures. In the revised manuscript, we have provided a more detailed description of the computational methods (see line 662-668). We have also added a citation to the Topaz.

14. Supplementary Table 1, and Supplementary Figures 2 and 4 – Please add Q-score for each dataset. Also, it is hard to believe that the local resolutions went all the way to 1.9 – 2.0 A (this is basically to the Nyquist limit) while the nominal resolutions are nearly an angstrom higher. Upon manual inspection of the map, I do not see any region that looks like 2.0 A-ish resolution. Authors will need to clarify what methods were used to calculate the range, and if possible, use stricter criteria as the numbers do not match the actual maps. Given the disparity, they should also detail this in the methods section.

**Reply:** We thank the reviewer for his/her valuable suggestions. We have indicated the Q-scores for the two complexes in Supplementary Table 5 (also see Table R4) based on the validation reports from the PDB Database. We calculated the local resolution distribution maps using the Local Resolution Estimation module in CryoSPARC, which were visualized with Chimera. We have updated the corresponding figures in Supplementary Figures 2 and 4 and the resolution range of the density maps in Supplementary Table 5. We have also added a detailed description of these analyses in the Methods section (see line 671-677).

Table R4. Data collection and refinement statistics of the inhibitors bound UT-A2 complex

| | **E822-1968-UT-A2** | **M353-0039-UT-A2** |
|---|---|---|
| Magnification | 130,000 | 130,000 |
| Voltage (kV) | 300 | 300 |
| Electron exposure ($e^-$/Å$^2$) | 60 | 60 |
| Defocus range (μm) | -0.8 ~ -2.0 | -0.8 ~ -2.0 |
| Pixel size (Å) | 0.92 | 0.92 |
| Symmetry imposed | C3 | C3 |

| | | |
|---|---|---|
| Initial particle images (no.) | 2,131,097 | 1,786,562 |
| Final particle images (no.) | 116,881 | 105,176 |
| B-factors of map-sharping | -99.2 | -94.7 |
| Map resolution (Å) | 2.9 | 2.7 |
| FSC threshold | 0.143 | 0.143 |
| Map resolution range (Å) | 2.7 ~12.3 | 2.2 ~9.9 |
| Initial model used (PDB code) | 8XD9, 8XD7 | 8XD9, 8XD7 |
| **Model composition** | | |
| Non-hydrogen atoms | 7620 | 7656 |
| Protein residues | 990 | 990 |
| Ligands | 3 | 3 |
| Q-score | 0.5570 | 0.5670 |
| **B factors ($Å^2$)** | | |
| Protein | 58.67 | 51.86 |
| Ligand | 57.68 | 54.96 |
| **R.m.s. deviations** | | |
| Bond lengths(A) | 0.009 | 0.008 |
| Bond angles (°) | 1.293 | 1.218 |
| **Validation** | | |
| MolProbity score | 2.24 | 1.65 |
| Clash score | 9.11 | 9.05 |
| **Ramachandran Plot** | | |
| Favored | 95.43 | 97.05 |
| Allowed | 4.75 | 2.95 |
| Disallowed | 0 | 0 |

15. Supplementary Figure 2 – specific to this dataset, GSFSC curve suggests presence of duplicated particles. Please try removing duplicate particles with spacing at least the size of the protein (Cryosparc has this function) then redo the reconstruction. If any particle drops out, the map/model will need to be redeposited and this figure set updated. (While all other figures will also need to be updated accordingly in theory, I will leave it to the editors to determine whether this is necessary – I expect the changes to be insignificant, and the updated ones will probably look almost identical to the current ones).

**Reply:** We thank the reviewer for his/her valuable suggestions. We used the Remove Duplicate Particles tool in CryoSPARC to eliminate duplicate particles, removing a total of 11,036 particles. We then performed Non-uniform Refinement using the remaining particles. The

results showed that the overall map resolution reduced slightly to 2.94 Å, compared to the original 2.86 Å. The two maps at different resolutions were nearly identical in overall features. We have refined the atomic model according to the newly calculated map. We analyzed the differences between the two topologies. Minor changes were observed in residue Q231 of the ligand binding pocket and some residues located in the N-terminal, C-terminal and LECL regions (Figure R6), which did not affect our core conclusions. We have updated the corresponding figures in the revised manuscript (including Figure 3d, 5c Supplementary Fig. 2b, 3b) based on the new model and map. Additionally, we generated a new validation report from the PDB database.

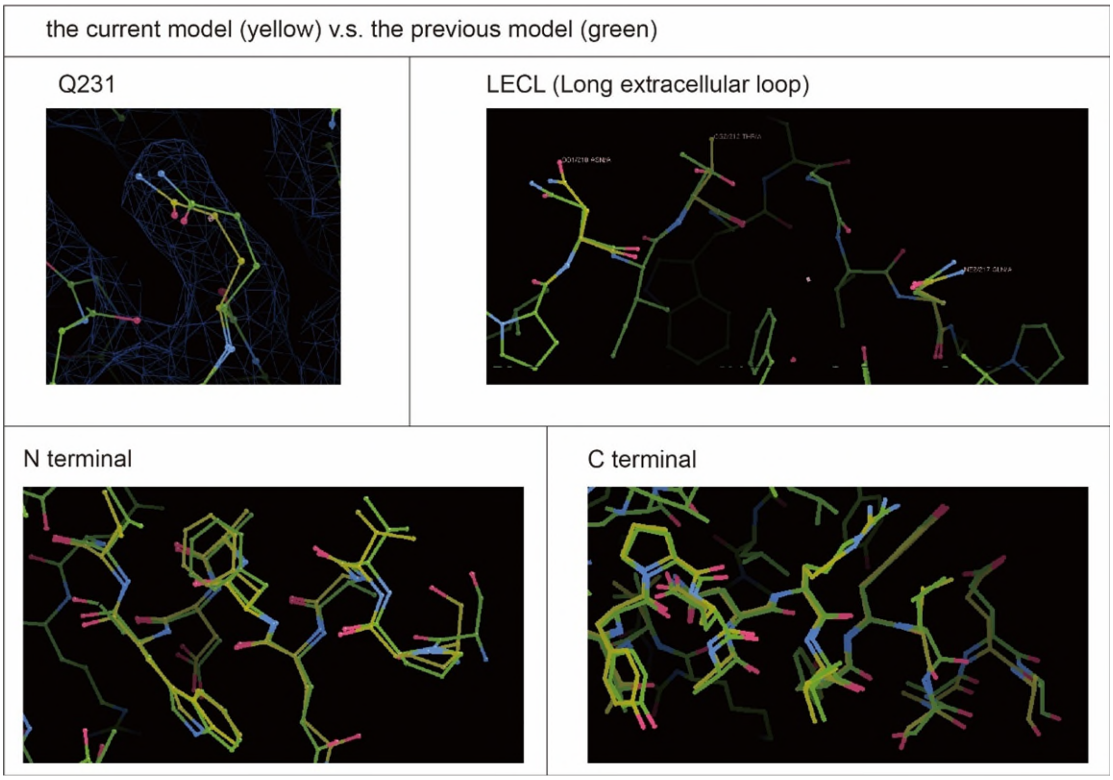


Figure R6. Comparison of major changes between the current model and the previous model on E822-1968-UT-A2 complex as shown in WinCoot. The current model was colored yellow, while the previous model was colored green.

16. Targeting UT-B, despite its wide distribution, may not be as bad as the authors make it out to be in the introduction (line 84 – 90). There are already humans with SLC14A1 knockouts and knockdowns – Jk(null) and Jk(weak) blood group phenotypes. Apart from mild diuresis, which the authors are actually aiming for, health effects are yet to be reported for these

individuals.

**Reply:** We thank the reviewer for his/her valuable suggestions. As reported in previous study, JK(null) people displayed mildly defect in maximal concentrating ability (818mosM/kg $H_2O$) compared to normal people (1025mosM/kg $H_2O$), and they did not show other obvious symptom or abnormalities (J Am Soc Nephrol. 1992 Jun;2(12):1689-96). In addition, Meng, Y. et al.'s results provided evidence that UT-B deletion causes progressive heart block in mice (Sci China Ser C 52, 474-478.; Proteomics 9, 504-511). The effect of UT-B inhibition on human needs further systematic research. We have changed relevant descriptions in the revised manuscript in line 85-89 as "*Phenotypic analyses of UT-B and UT-A knockout mice have uncovered some different roles besides the urea concentration in renal. Cardiac conduction defects were found in mice deficient in UT-B. These findings highlight potential off-target effects and extrarenal complications arising from non-selective UT inhibition.*"

17. Regarding the observations with mouse studies (effect only in low-protein diet group), is there a possibility that the compound's effect on the liver is contributing to this?

**Reply:** We thank the reviewer for his/her valuable suggestions. As we mentioned in **Question 5**, Klein JD et al. have suggested the possible presence of a UT-A2 isoform protein in hepatocytes (J Am Soc Nephrol 10, 2076-2083). We performed cell-based experiments and found that hepatocytes do indeed express UT-A2 protein as M353 is an UT-A2 selective inhibitor. As to whether hepatocytes express other types of UT protein isoforms, and to what extent UT-A2 contributes to hepatic urea transport, our current data cannot draw definitive conclusions; future studies will be required to address these questions. Although we observed an effect of M353-0039 on urea transport efficiency in HepG2 cells, the impact was notably limited. Even at sufficient concentrations, it inhibited urea transport by only 10-15% (Figure R5 in Question 8). Moreover, most UT inhibitors generally do not produce liver-related adverse effects (Biomedicines. 13(4):992; FASEB J. 38(13):e23760; Biomedicines. 11(2):607; Acta Pharm Sin B. 11(1):181-202.). We also measured the blood and kidney drug concentrations 6 hours after administration, which showed that the compound primarily accumulated in the kidneys (Figure R7, also see Supplementary Figure 6). Therefore, based on the current data, the compound's main pharmacological action is theoretically concentrated in renal tissues. We also

noted that M353-0039, being a chemical probe from virtual screening rather than a lead compound, demands careful scrutiny regarding its potential liabilities in biological studies. We have included the corresponding description to the Discussion section (line 406-416) as "*Notably, the fact that most UT inhibitors do not exhibit hepatotoxicity seems to suggest that UTs play only a minor role in the liver. Cytotoxicity assays indicated that M353-0039 exhibits no toxicity at high concentrations. In addition, we determined its plasma and renal concentrations, which demonstrated that M353-0039 accumulated mainly in the kidney 6 hours after administration. Therefore, the use of M353-0039 may provide a more convenient and economical pharmaceutical intervention procedure, in addition to specific knockout mice, for studying the unique functions of the UT-A2 subtype without affecting the functions of UT-A3 and UT-B. In the meantime, as a chemical probe derived from virtual screening rather than a lead compound, M353-0039 warrants careful attention to potential liabilities when used in biological studies.*"

A

| Animal | Blood concentration (ng/ml) | Kidney concentration (μg/g) |
|---|---|---|
| 1 | 16.74 | 0.17 |
| 2 | 19.67 | 0.59 |
| 3 | 7.29 | 0.44 |
| Mean±SEM | 14.57±3.74 | 0.40±0.12 |

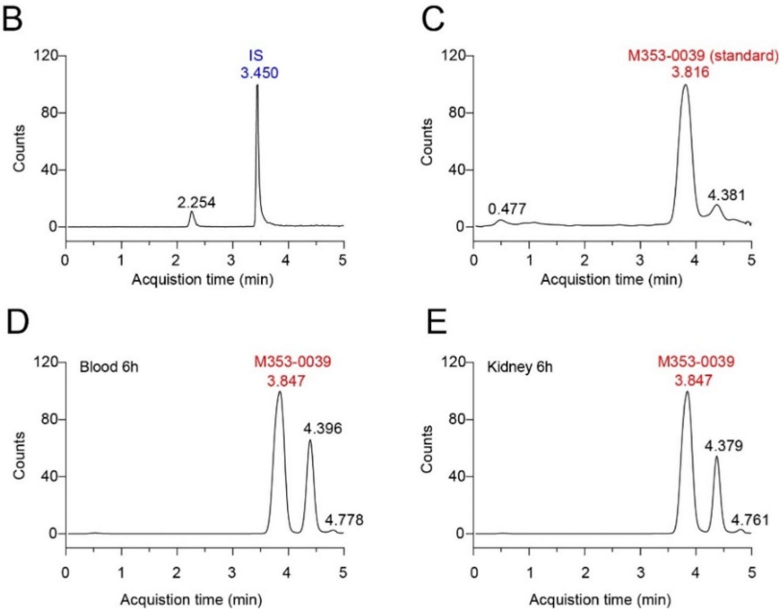

Figure R7. The concentration of M353-0039 in mouse blood and kidney after 6 h intraperitoneal administration. (A) The calculated M353-0039 concentration of blood and kidney sample from three mice. (B) Representative UPLC chromatogram of Internal standard berberine. (C) Representative UPLC chromatogram of M353-0039 standard. (D) Representative UPLC chromatogram of M353-0039 in blood sample after 6 h administration. (E) Representative UPLC chromatogram of M353-0039 in kidney sample after 6 h administration.

18. Please check citations and add/update as necessary. For example, no citation is provided for HQA2. DoGsitescorer (which I believe the authors are referring to - last "r" is missing) needs citation as well.

**Reply**: We thank the reviewer for his/her valuable suggestions. We have checked all citations throughout the manuscript and added necessary literature references where appropriate in line 111.

Reviewer #3 (Remarks to the Author):

Huang et al. report the discovery and structural characterization of small-molecule inhibitors of the human urea transporter UT-A2. Through virtual screening and functional characterization, they identify two chemotypes (E822-1968 and M353-0039) that bind selectively to UT-A2 and solve cryo-EM co-structures at 2.9 Å and 2.7 Å resolution. M353-0039 inhibits UT-A2 with an $IC_{50} \approx 0.35$ µM and shows marked selectivity over UT-A1/A3 and UT-B. Structural analyses implicate non-conserved residues C285 and G322 in a “T–T” subpocket as the determinants of selectivity, confirmed by mutagenesis. In vivo administration in mice reduces renal medullary urea and osmolality, and HepG2 assays indicate cellular inhibition of urea transport. The study provides a credible structural and functional basis for selective UT-A2 probes and a possible route toward next-generation diuretics.

The manuscript is technically sound and addresses an unmet pharmacological need, but several aspects of experimental rigor, nomenclature, and mechanistic interpretation should be revised before publication.

**Reply**: We thank the reviewer for his/her valuable comments.

Major Comments

1. The lead compound alternates between M353-0039, M353-0059, and M353-0093 across figures and methods (e.g., Fig. 1, Methods §2.3, Extended Data Fig. 2). The authors must verify all identifiers and ensure uniform labelling throughout the text and supplementary materials.

**Reply**: We thank the reviewer for his/her valuable suggestions. We have corrected these errors and conducted a thorough review and revision of the entire manuscript. The correction was seen in line 316, line 722, line 971, line 975, line 978, line 1016, line 1019 of manuscript and in the title of Supplementary Table 5 and Supplementary Table 7.

2. Many panels employ unpaired two-tailed t-tests with n = 4–5. Please clarify whether n represents biological or technical replicates, whether randomization/blinding was applied, and provide justification or use ANOVA with multiple-comparison correction. Report animal sex, age, and inclusion/exclusion criteria.

**Reply**: We thank the reviewer for his/her valuable suggestions. In the animal experiments, n represents biological replicates, as added to the figure legends. The experiment did not involve randomization or blinding; instead, we grouped the mice based on their urine volume during a 24-hour adaptation period and ensured that the urine volume of the control and treatment groups was similar before administration to minimize potential biases. Since the urine volume could not be matched one-to-one, we used an unpaired two-tailed t-test for statistical analysis. The animal sex, age, and inclusion criteria have been added to the Methods section at line 702-707.

3. The 80 mg $kg^{-1}$ i.p. dosing lacks formulation, PK/PD, and tolerability information. Please include vehicle composition, exposure data, and, if possible, plasma or kidney compound concentrations. A UT-A2-knockout control (or demonstration of no further effect) would confirm on-target activity.

**Reply**: We thank the reviewer for his/her valuable suggestions. Since the primary focus of this study is to develop the strategy for screening UT-A selective inhibitors, and M353-0039 is merely one inhibitor molecule identified as a chemical probe under this strategy, we did not conduct comprehensive druggability evaluations on it. We have added essential clarifications along with pertinent experimental data in the revised version.

(I) In the revised Methods section, we have supplemented the formulation of M353-0039 as saline+1% DMSO, which served as the vehicle composition for our experiments. Additionally, we assessed the compound's safety and found no significant cytotoxicity at a concentration of 100 μM (Supplementary Figure 5i, also see Figure R8) We also measured the plasma and kidney concentrations of M353-0039 at the sampling time point after administration, yielding values of 14.57±3.74 ng/ml (plasma) and 0.40±0.12 μg/g (kidney), respectively. These data are shown in Supplemental Fig. 6 (also see Figure R9). We also noted that M353-0039, being a chemical probe from virtual screening rather than a lead compound, demands careful scrutiny regarding its potential liabilities in biological studies in the Discussion. We have included the corresponding description to the Discussion section (line 408-416) as "*Cytotoxicity assays indicated that M353-0039 exhibits no toxicity at high concentrations. In addition, we determined its plasma and renal concentrations, which demonstrated that M353-0039 accumulated mainly in the kidney 6 hours after administration. Therefore, the use of M353-0039 may provide a more convenient and economical pharmaceutical intervention procedure, in addition to specific knockout mice, for studying the unique functions of the UT-A2 subtype without affecting the functions of UT-A3 and UT-B. In the meantime, as a chemical probe derived from virtual screening rather than a lead compound, M353-0039 warrants careful attention to potential liabilities when used in biological studies.*"

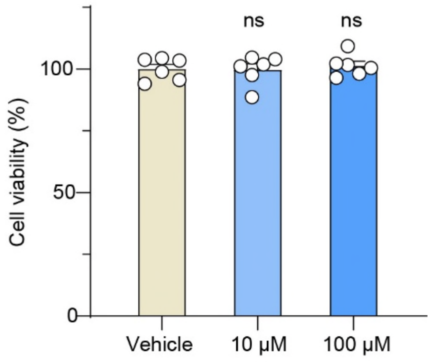


Figure R8. The CCK-8 cytotoxicity assay results indicated that M353-0039 treatment at the tested concentrations (10 μM, 100 μM) did not show significant cytotoxicity. statistical differences were determined by the two-sided unpaired Student's t-test compared with the vehicle.

A

| Animal | Blood concentration (ng/ml) | Kidney concentration (μg/g) |
|---|---|---|
| 1 | 16.74 | 0.17 |
| 2 | 19.67 | 0.59 |
| 3 | 7.29 | 0.44 |
| Mean±SEM | 14.57±3.74 | 0.40±0.12 |

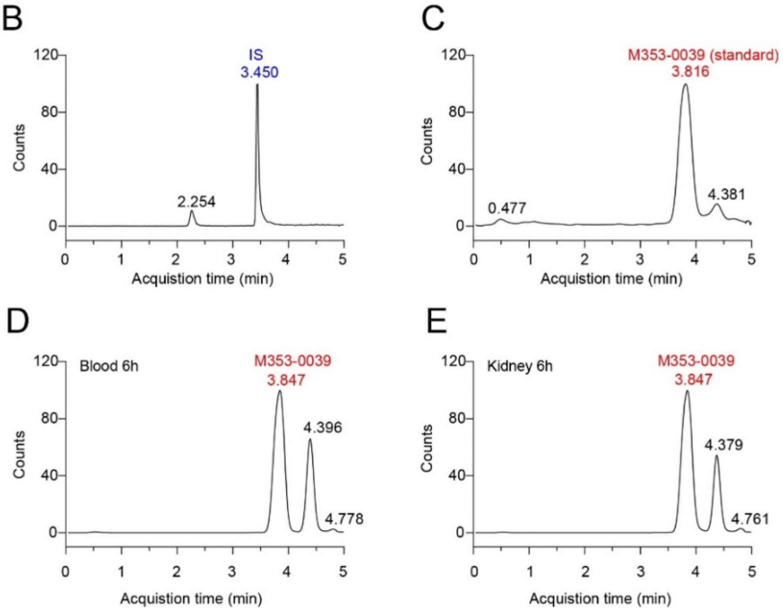


Figure R9. The concentration of M353-0039 in mouse blood and kidney after 6 h intraperitoneal administration. (A) The calculated M353-0039 concentration of blood and kidney sample from three mice. (B) Representative UPLC chromatogram of Internal standard berberine. (C) Representative UPLC chromatogram of M353-0039 standard. (D) Representative UPLC chromatogram of M353-0039 in blood sample after 6 h administration. (E) Representative UPLC chromatogram of M353-0039 in kidney sample after 6 h administration.

(II) Uchida, S. et al. have reported that renal urea transport impairment was observable in UT-A2-knockout mice without any administration of UT inhibitors (Mol Cell Biol. 25(16):7357-63). Therefore, we failed to use the UT-A2-knockout mice as the control group. In this study, we utilized wild-type mice administered the UT-A2 selective inhibitor M353-0039 to simulate the effects observed in UT-A2 knockout mice. The results demonstrated that M353-0039-treated wild-type mice exhibited responses comparable to those of UT-A2 knockout mice previously reported.

4. Line 255: The term allosteric pocket implies non-competitive inhibition, yet no competition or urea-dependence analyses (i.e. Schild plot) are presented. Unless such pharmacological evidence is added, replace "allosteric pocket" with "extrachannel subpocket on the extracellular face" or similar, and explicitly note that competitive versus non-competitive mechanism has not been established.

**Reply**: We thank the reviewer for his/her valuable suggestions. We have replaced the "allosteric pocket" with "extrachannel subpocket on the extracellular face" in the revised manuscript at line 267-268. Given that the impact of distinct action types (competitive versus non-competitive) on inhibitory effectiveness has not yet been established, we have refrained from further exploration on the inhibitory mechanism of M353-0039. We have included these descriptions to the Discussion section at line 370-374 as "*Although the action types (competitive or non-competitive) of M353-0039 have not yet been established, these observations revealed crucial regions and key residues, which provided useful information for designing selective inhibitors targeting various UT subtypes as well as guidance for conducting efficient structure-based modifications of UT inhibitors.*"

5. Mean B-factors of 3 Å² for protein/ligand are implausibly low. Please confirm refinement parameters, describe map-sharpening settings, and provide map–model FSC curves. Ensure that the deposited PDB/EMD entries correspond to these maps and include validation statistics.

**Reply**: We thank the reviewer for his/her valuable suggestions. We have verified the cryo-EM calculation parameters and corrected erroneous entries in the parameter tables (see Supplementary Table 5 or Table R5). The B-factors of map sharping were -99.2 and -94.7 for E822-1968-UT-A2 and M353-0039-UT-A2 complexes, respectively. For the E822-1968UT-A2 complex, the mean B-factors were 58.67 for the protein and 57.68 for the ligand; for the M353-0039-UT-A2 complex, the mean B-factors were 51.86 for the protein and 54.96 for the ligand; The map-model FSC curves validated by Phenix are provided in Figure R10, also see Supplementary Figure 2e and 4e. We also validated the correctness of the files submitted to PDB/EMDB for deposition

Table R5. Data collection and refinement statistics of the inhibitors bound UT-A2 complex

| | E822-1968-UT-A2 | M353-0039-UT-A2 |
|---|---|---|
| Magnification | 130,000 | 130,000 |
| Voltage (kV) | 300 | 300 |
| Electron exposure ($e^{-}$/Å$^2$) | 60 | 60 |
| Defocus range (μm) | -0.8 ~ -2.0 | -0.8 ~ -2.0 |
| Pixel size (Å) | 0.92 | 0.92 |
| Symmetry imposed | C3 | C3 |
| Initial particle images (no.) | 2,131,097 | 1,786,562 |
| Final particle images (no.) | 116,881 | 105,176 |
| B-factors of map-sharping | -99.2 | -94.7 |
| Map resolution (Å) | 2.9 | 2.7 |
| FSC threshold | 0.143 | 0.143 |
| Map resolution range (Å) | 2.7 ~12.3 | 2.2 ~9.9 |
| Initial model used (PDB code) | 8XD9, 8XD7 | 8XD9, 8XD7 |
| **Model composition** | | |
| Non-hydrogen atoms | 7620 | 7656 |
| Protein residues | 990 | 990 |
| Ligands | 3 | 3 |
| Q-score | 0.5570 | 0.5670 |
| **B factors (Å$^2$)** | | |
| Protein | 58.67 | 51.86 |
| Ligand | 57.68 | 54.96 |
| **R.m.s. deviations** | | |
| Bond lengths(A) | 0.009 | 0.008 |
| Bond angles (°) | 1.293 | 1.218 |
| **Validation** | | |
| MolProbity score | 2.24 | 1.65 |
| Clash score | 9.11 | 9.05 |
| **Ramachandran Plot** | | |
| Favored | 95.43 | 97.05 |
| Allowed | 4.75 | 2.95 |
| Disallowed | 0 | 0 |

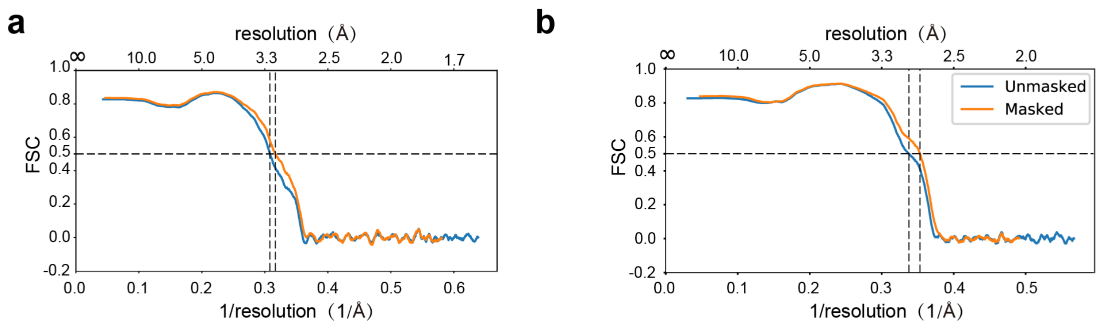


Figure R10. The map-model FSC curves of E822-1968-UT-A2 (a) and M353-0039-UT-A2 (b) complexes validated by Phenix.

6. Functional assays use human UT-A2, while in vivo studies use mouse. Include cross-species sequence alignment to show conservation of interacting residues.

**Reply**: We thank the reviewer for his/her valuable suggestions. A pairwise sequence alignment was carried out for the human and murine proteins. The interacting residues (colored red) of the two different species are completely identical. The comparative data are displayed in Supplementary Figure 5f (also see Figure R11).

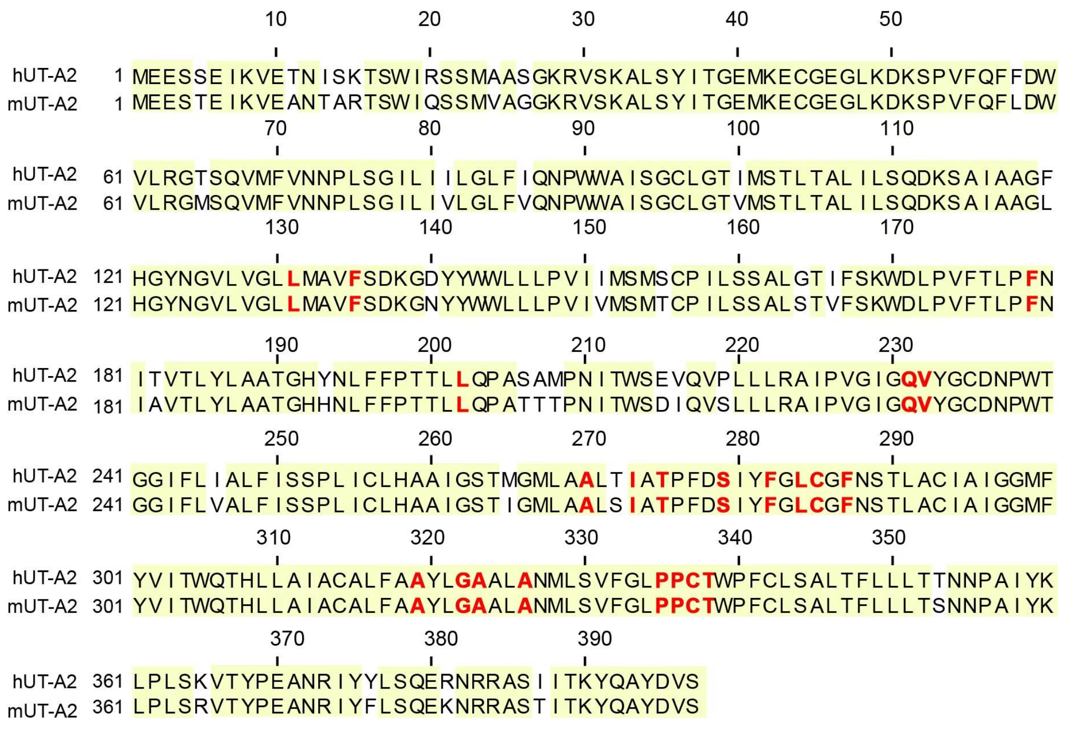


Figure R11. Amino acid sequence alignment of human UT-A2 and mouse UT-A2. The same amino acids were shown as light yellow background. The residues of the binding pocket (colored red) of M353-0039 in such two different species are completely identical.

7. The predominant hepatic UT isoform in HepG2 is uncertain. Authors should at minimum present qPCR or Western blot data confirming UT-A2 expression in HepG2 cells.

**Reply**: We thank the reviewer for his/her valuable suggestions. We have performed the qPCR to confirm the expression of UT-A2 in HepG2 cells. All results from three specific primer pairs indicated that UT-A2 expression could be detected in HepG2 cells. The primers and electrophoresis data are displayed in Supplementary Figure 5g-h (also see Figure R12).

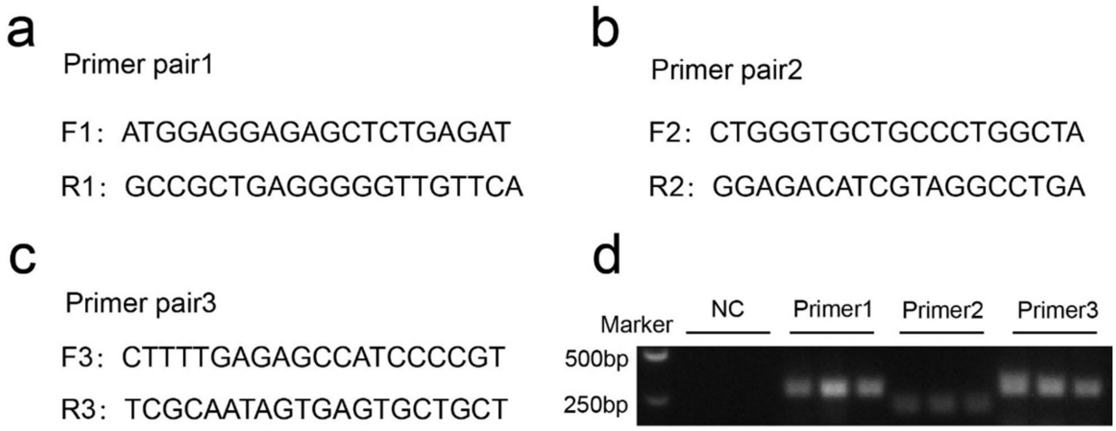


Figure R12. Detection of UT-A2 expression in HepG2 cells by RT-qPCR. Three primer pairs for human UT-A2 (a-c) and the electrophoresis band of qPCR product were shown as above (d). NC, negative control, use ultrapure water as the template and primer 1 as the primer for qPCR.

8. No hERG, CYP, or cytotoxicity data are reported. At minimum, include a statement clarifying that these compounds serve as chemical probes rather than leads, and note any in-silico or panel screens used to flag potential liabilities.

**Reply**: We thank the reviewer for his/her valuable suggestions. We assessed cytotoxicity and found that M353-0039 was not cytotoxic even at a high concentration (100 μM) in Figure R8. We also added a clarification in the Discussion section: *"In the meantime, as a chemical probe derived from virtual screening rather than a lead compound, M353-0039 warrants careful attention to potential liabilities when used in biological studies."* (line 414-416)

9. The urease-coupled assay provides an indirect readout of urea flux. While the cryo-EM structures convincingly establish direct compound binding to UT-A2, an orthogonal functional

assay (e.g., radiolabeled or LC–MS urea flux, or stopped-flow light scattering) would help confirm that this structural binding event indeed translates to transport inhibition rather than a non-conducting conformational lock."

**Reply**: We thank the reviewer for his/her valuable suggestions. We performed absolute quantitative analysis of intracellular urea concentrations before and after antagonist treatment using the UHPLC-MS/MS method. The results showed that, compared with the control group, M353-0039 significantly increased the intracellular urea concentration level (Figure R13, also see Supplementary Fig. 7e-f). These findings were consistent with the results of the urease assay, indicating that M353-0039 effectively blocks the rapid transmembrane transport of urea mediated by UT.

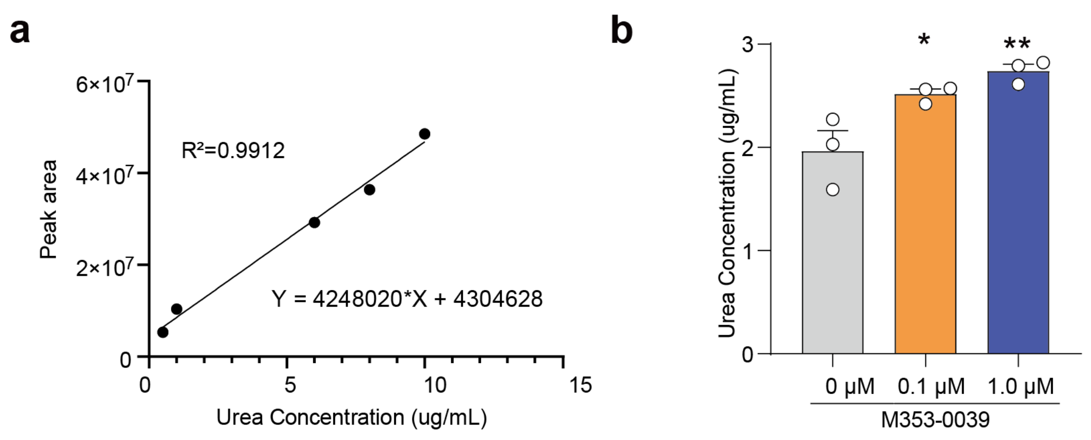


Figure R13. Determination of the absolute urea concentration. (a) Standard curve of peak area vs. urea concentration. (b) 293F cells overexpressing UT-A2 were incubated with 50 mmol/L urea and washed with HBSS containing various concentrations of M353-0039; M353-0039 treatment markedly elevated intracellular urea levels. $^{**}P < 0.01$; $^{*}P < 0.05$, One-way ANOVA with Tukey's test (compared with the 0 μM). Values are mean ± SEM from three independent experiments (n = 3).

Minor Comments

10. • Unify abbreviations for binding pockets (ATCP, EUBP, EBBP, T–T). Provide a schematic in the Supplementary.

**Reply:** We thank the reviewer for his/her valuable suggestion. We have provided a detailed description of all the pockets involved in this study in Supplementary Table 1 (also see Table

R8).

Table R8. The diverse pockets of urea transporter UT-A2 for inhibitor interaction.

| Pockets | Universal name | Description | Reference |
|---|---|---|---|
| EUBP | The extracellular urea binding pocket | This urea molecule binding pocket is located in the region of the UT-A2 urea transport channel near the extracellular side, primarily comprising the amino acids L131, F179, Q231, V232, F287, C337 and T338. | Nat Commun. 2024 Nov 26;15(1):10226. |
| EBBP | The extracellular blocker binding pocket | Located on the extracellular side of urea transport channel in the UT molecular, the primary binding region for inhibitors includes two sub-pockets, EBBP1 and EBBP2, primarily comprising the amino acids F135, L202, F282, L284, P335, and P336. | Nat Commun. 2024 Nov 26;15(1):10226. |
| SCGP | “SCG” pocket | This pocket is completely independent of the urea transport channel of UT-A2 and is composed of T275, S279, C285, G322 and A326 from UT-A2. The residues of the SCG pocket region are not conserved among different UTs, particularly at positions S279, C285 and G322, making it a potential target pocket for developing UT-selective inhibitors. | Nat Commun. 2024 Nov 26;15(1):10226. |
| ATCP | “ATC” pocket | Located on the extracellular side of UT-A2 and independent of both the urea transport channel and the EBBP region, this subpocket is enclosed by residues A270, I273, T275, C285, and A323. | This article |
| “T-T” pocket | The binding pocket of the thiophene-thiazole group | Located on the extracellular side of UT-A2 and independent of its urea transport channel, this region serves as the binding site for the thiophene-thiazole group of inhibitor M353-0039, involving residues A326, A319, C285, T275, A270, I273, G322, A323, L284 and P336. | This article |
| L-P pocket | L-P pocket | The region near L202 and P335 on the EBBP of UT-A2, where these two residues are not conserved across different UT subtypes, represents a potential binding pocket for developing UT-selective inhibitors. | Nat Commun. 2024 Nov 26;15(1):10226. |

11. • Language and typos:

o Fig. 1 A: correct “in vitro” and “structural analysis.”

o Line 95: “our works” → “our work.”

o Line 97: "...as next-generation diuretics."

o Line 255: replace "allosteric pocket" per above.

**Reply**: We thank the reviewer for his/her valuable suggestions. We have corrected these errors in Figure 1a, in line 101, in line 103, in line 267-268, and conducted a thorough review and revision of the entire manuscript.

12. • Supplementary Fig. 5d (CCK-8): specify cell line, seeding density, exposure time, vehicle, n, and statistical test

**Reply**: We thank the reviewer for his/her valuable suggestions. We have added a description of this part in the Methods section (line 612-620), under the subsection titled Cytotoxicity assay.

13. • Supplementary Table 1: verify mean B-factors and include map-sharpening parameters.

**Reply**: We thank the reviewer for his/her valuable suggestions. We have verified the cryo-EM calculation parameters and corrected erroneous entries in the parameter tables (see Supplementary Table 5 or Table R5). The B-factors of map sharpening were -99.2 and -94.7 for E822-1968-UT-A2 and M353-0039-UT-A2 complexes, respectively. For the E822-1968UT-A2 complex, the mean B-factors were 58.67 for the protein and 57.68 for the ligand; for the M353-0039-UT-A2 complex, the mean B-factors were 51.86 for the protein and 54.96 for the ligand.

14. • Minor style issues: e.g., "Data was" → "Data were," "reach equilibrium state" → "reach equilibrium"

**Reply**: We thank the reviewer for his/her valuable suggestions. We have corrected these errors and conducted a thorough review and revision of the entire manuscript.

Reviewer #4 (Remarks to the Author):

This manuscript reports the discovery of novel selective urea transporter inhibitors through an integrated approach combining high-throughput screening, pharmacological validation, and structural characterization. While the overall strategy is sound and the results are highly interesting, several aspects require clarification and additional detail prior to publication:

**Reply**: We thank the reviewer for his/her valuable comments.

Major Comments

1. Custom database design (line ~113): The custom-designed database is insufficiently described in the main text and also the Methods section. Please provide detailed information on how this database was constructed, what criteria were used for compound selection, and what distinguishes it from the original database used.

**Reply**: We thank the reviewer for his/her valuable suggestions. The "custom-designed database" described in this article refers to a specialized virtual screening compound library targeting UT-A2, constructed by systematically screening the commercial compound database ChemDiv (approximately 1 million compounds) based on structural and physicochemical properties. The database construction process includes the following steps:

First, physicochemical property and chemical quality control screening were performed. All ChemDiv compounds were evaluated for drug-likeness according to Lipinski's rule of five, combined with PAINS (pan-assay interference compounds) filtering and removal of reactive or potentially toxic functional groups, to eliminate non-drug-like, chemically unstable molecules or those that might introduce experimental false positives, thus ensuring the reliability of subsequent virtual screening.

Subsequently, target-structure-based screening and enrichment were conducted. By analyzing the structure of the UT-A2 binding pocket and combining it with the residues of UT-A2 hotspot identified in preliminary molecular docking results, pharmacophore constraints matching this pocket were constructed. Based on this, compounds with features complementary to the UT-A2 extracellular pocket were retained, including rationally distributed hydrogen bond donor/acceptor sites and aromatic or hydrophobic structural units compatible with the pocket's hydrophobic patches, thereby achieving target-centric compound enrichment.

After completing the above screening, the remaining approximately 45,000 compounds underwent further structural diversity control. Specifically, Bemis-Murcko framework definitions were used to cluster the compounds based on their scaffolds (Tanimoto similarity threshold set to 0.6) to reduce structural redundancy and maximize scaffold diversity. Finally,

approximately 10,000 representative compounds were selected to form the custom database for the first round of virtual screening.

Compared to the original ChemDiv database, this custom database exhibits distinct target-centric and function-oriented characteristics: while retaining chemical diversity, it significantly reduces the redundancy of general drug-like compounds and enriches molecules that are structurally more likely to fit the UT-A2 extracellular binding pocket and possess potential functional blocking capabilities. In the second round of screening, based on the further clarified ATCP and EUBP sub-pockets derived from structural analysis, we followed the same database construction process, but refined the pocket definition and screening constraints to achieve more focused structure-guided screening and improve the enrichment efficiency of functional inhibitors.

The above database construction process and related parameters have been added to the Methods in the “Virtual screening of UT-A2 inhibitors” section of the revised manuscript at line 472-511.

2. Docking validation: The screening workflow appears to lack validation. Was a ROC curve analysis or similar benchmarking performed to assess the docking protocol's performance? Validation against known actives and decoys would strengthen the computational predictions.

**Reply**: We thank the reviewer for his/her valuable suggestions. We used the previously reported inhibitor HQA2 and its analogues as the "active ingredient" and randomly selected 300 matching decoys from the ChemDiv database using the DUD-E protocol. Using the same docking settings as the virtual screening, ROC curve analysis yielded an AUC of 0.82 (Figure R14), indicating good distinguishability between active and inactive molecules. Further comparison showed that HQA2 binds in a similar pose in the cryo-EM structure and the docking model, with an RMSD of only 1.9 Å, which supported the validity of our docking parameters and scoring function. These results confirm that our docking workflow can reliably identify potential binding poses in UT-A2 hotspot pockets. We have added this validation description and ROC curve as Supplementary Figure 7a-b, and cited in the Methods section "Docking Validation" at line 512-527: “*To validate the docking workflow, retrospective benchmarking was performed using previously reported UT inhibitors, including HQA2 and its analogs (11*

*compounds), as actives, and 300 physicochemically matched decoys selected from the ChemDiv database following the DUD-E protocol; docking was carried out using the same Glide SP settings as applied in the virtual screening workflow. Receiver operating characteristic (ROC) curve analysis yielded an area under the curve (AUC) of 0.82 (Supplementary Fig. 7a), indicating good discrimination between active and inactive compounds, and structural validation by docking HQA2 into the UT-A2 cryo-EM structure reproduced the experimentally observed binding pose with a root-mean-square deviation (RMSD) of 1.9 Å (Supplementary Fig. 7b), which supported the validity of our docking parameters and scoring function. We have also compared the docking-predicted binding modes with the experimentally determined cryo-electron microscopy structures of the UT-A2-E822-1968 and UT-A2-M353-0039 complexes (Supplementary Fig. 7c-d). The results show similarity between the calculated predictions and experimental data. These results confirm that our docking workflow reliably predicts the correct orientation and interaction modes of the two inhibitors prior to experimental structure determination.*"

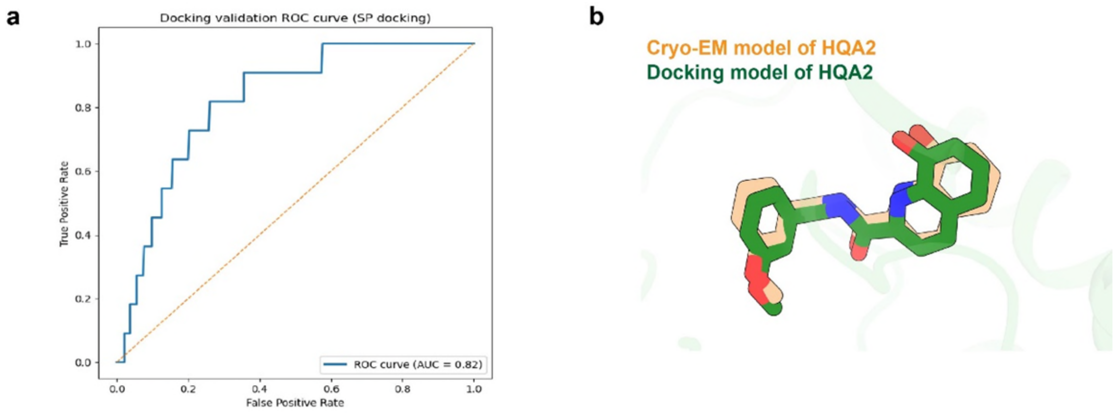


Figure R14. Validation of the docking workflow.

**a.** ROC curve evaluating the performance of the docking protocol using previously reported UT inhibitors (HQA2 and its analogs) as actives and 300 physicochemically matched decoys selected from the ChemDiv database following the DUD-E protocol. Docking was performed using the same Standard Precision (SP) settings as applied in the virtual screening workflow. The resulting area under the curve (AUC) of 0.82 indicates good discrimination between active and inactive compounds.

**b.** Structural validation of the docking protocol by comparison of the predicted docking pose

of HQA2 with its experimentally determined cryo-EM binding mode in UT-A2. The docked pose closely overlaps with the cryo-EM model, with a root-mean-square deviation (RMSD) of 1.9 Å, supporting the reliability of the docking parameters and scoring function.

3. Hit selection (line ~117): The manuscript identifies certain residues as pivotal for binding but then excludes molecules that interact with these regions. This seems contradictory—please clarify the reasoning for excluding molecules that engage with the defined binding pocket.

**Reply**: We thank the reviewer for his/her valuable suggestions. In our previous work, we demonstrated that the residues in the SCG pocket are not conserved across UT isoforms (UT-A2, UT-A3, and UT-B) (Nat Commun. 2024 Nov 26;15(1):10226.). Consequently, targeting this pocket for small-molecule screening enhances the probability of identifying selective inhibitors. A corresponding description has been inserted at line 122-124 of the manuscript as follow: "*Notably, the residues identified in the "SCG" pocket were considered pivotal for inhibitor selectivity, because the residues lining this pocket are non-conserved across different UT subtypes.*"

4. Screening workflow (line ~179): The second screening workflow targeting the deeper pocket needs more detailed description.

**Reply**: We thank the reviewer for his/her valuable suggestions. In the revised manuscript, we expanded the description of the second round of the "updated hotspot pocket" virtual screening workflow, which specifically targeted the EUBP and the newly discovered ATCP sub-pockets in UT-A2 that may increase affinity. This cavity was defined as a new binding pocket using the DoGSiteScorer algorithm and visualized in Schrödinger Maestro. Based on this improved pocket, we reconstructed a target-focused compound library by selecting molecules containing polar or aromatic groups complementary to residues F135, Q231, A270, T275, C285, F287, and A323, which define the new hotspot pocket and are simplified into a pocket grid; the 10,000 compounds from the first round of the ChemDiv library were rescored using the Glide SP method combined with XP docking. The top 100 compounds were visually sorted based on their interactions with ATCP and EUBP, and finally, five compounds were selected for testing and analysis. Ultimately, compound M353-0039 showed potent and highly selective UT-A2 inhibitory activity. These extended descriptions have been added to the " Virtual screening of

UT-A2 inhibitors " subheading in the "Methods" section (revised lines 496-511).

5. Comparison with experimental data: It would strengthen the manuscript to compare your docking predictions with available cryo-EM data. How well do the predicted binding modes align with experimental structures?

**Reply**: We thank the reviewer for his/her valuable suggestions. We have compared the docking-predicted binding modes with the experimentally determined cryo-electron microscopy structures of the UT-A2-E822-1968 and UT-A2-M353-0039 complexes. The results show similarity between the calculated predictions and experimental data. These results confirm that our docking workflow reliably predicts the correct orientation and interaction modes of the two inhibitors prior to experimental structure determination. The corresponding plot and quantitative comparison values have been added as Supplementary Figure 7c-d and are described in the Results and Methods section at line 522-527.

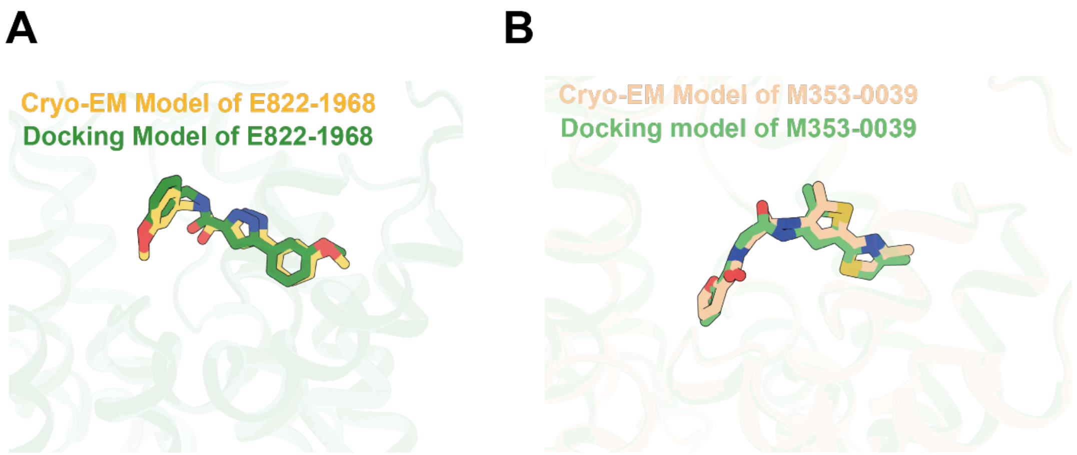


Figure R15. Comparison between docking-predicted and cryo-EM binding conformations of UT-A2 inhibitors. Superposition of the docking model and cryo-EM structure of E822-1968(a) and M353-0039(b) bound to UT-A2, showing similarity between the predicted and experimentally determined binding poses.

Minor Comments

6. SI Figure 1c: Please label more clearly that this is a sectional/cut view of the protein from the side. The orientation isn't immediately apparent.

**Reply**: We thank the reviewer for his/her valuable suggestions. We have updated SI Figure 1c as requested; the revised version is shown below (Figure R16).

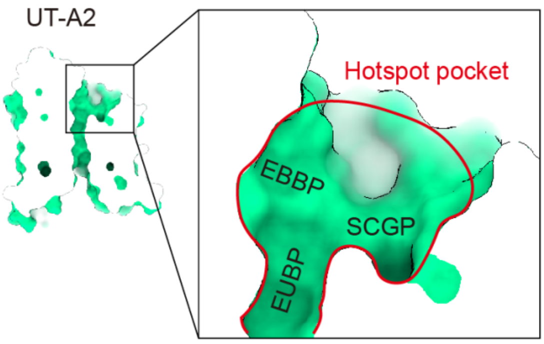


Figure R16. Cutway view of the hotspot pocket on UT-A2, which includes the regions of extracellular urea binding pocket (EUBP), extracellular blocker binding pocket (EBBP) and the "SCG" pocket (SCGP).

7. Line ~240 - Hydrophobic interactions terminology: When describing protein-ligand interactions, consider using "hydrophobic contacts" rather than focusing solely on van der Waals forces. The term "van der Waals forces" describes only the enthalpic component and disregards the entropic energy gains from releasing water from the binding pocket, which is actually the main driving force for these interactions.

**Reply**: We thank the reviewer for his/her valuable suggestions. We have replaced the term “van der Waals forces” with “hydrophobic interaction” in describing protein-ligand interactions within these two pockets in line 250.

8. Line ~179 - "Filtration" terminology: The term "filtration" sounds imprecise in this context. Consider rephrasing to "we implemented a filtering strategy" or similar.

**Reply**: We thank the reviewer for his/her valuable suggestions. We have replaced the term “filtration” with “filtering” in line 187.

9. Line ~405 - "Enhanced sampling": This term is typically used for enhanced sampling in molecular dynamics simulations (metadynamics, replica exchange, etc.). If you're not referring to these techniques, please use alternative phrasing to avoid confusion.

**Reply**: We thank the reviewers for pointing out this potential misleading information. We did not use molecular dynamics-enhanced sampling methods (such as metadynamics or replica exchange). We have updated the detailed methods in “Virtual screening of UT-A2 inhibitors” of the revised manuscript at line 484-511.

10. Figure 4 - Chemical structures: The structure drawing of compound E762-1894 (and to a lesser degree the other compounds) does not adhere to standard conventions for chemical structure representation. Please redraw using software such as ChemDraw with proper bond angles, consistent formatting.

**Reply**: We thank the reviewer for his/her valuable suggestions. All chemical structures presented in the article have been re-drawn with ChemDraw (Figure R17) and incorporated into the corresponding figures.

M353-0039

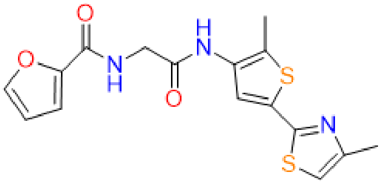

D400-1574

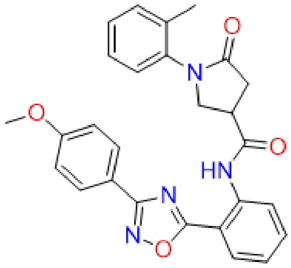

8012-3374

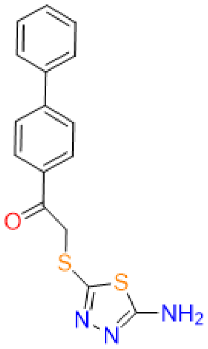

D103-1163

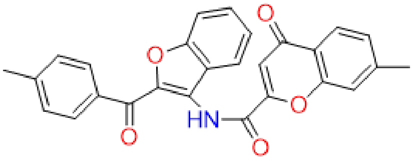

E822-1968

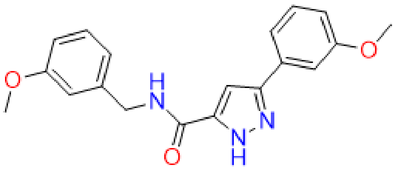

E762-1894

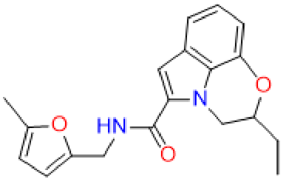

E3

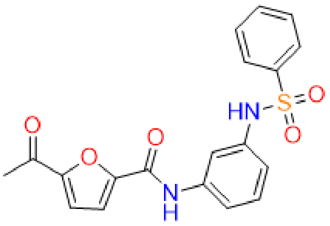

HQA2

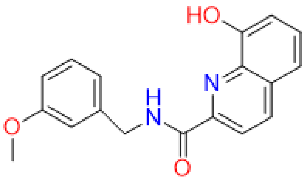

25a

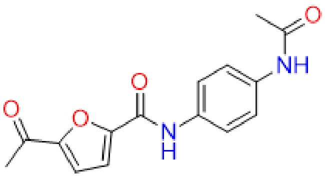

Figure R17. All chemical structures presented in the article have been re-drawn with ChemDraw

**Responses to reviewers' comments**

We thank the reviewers for their valuable time in reviewing our manuscript and the helpful suggestions that they have provided. We have carefully taken these comments and suggestions from the reviewers into careful consideration in preparing our revised version, which has resulted in a more thorough and clear manuscript. Please find below a point-by-point response to the comments with our responses in ***Blue*** and the referees' comments in ***black***.

**Reviewer's Comments:**

Reviewer #1 (Remarks to the Author)

This is a data rich and extremely technical manuscript that identifies and provides evidence for an apparently specific inhibitor of the UT-A2 urea transporter. In response to the original review, the manuscript has been improved by adding some details, defining some acronyms, and enhancing the discussion. There are a couple of remaining small issues in the text that were apparent, but mostly all original comments have been addressed. Below is a list of the comment responses.

**Reply:** We thank the reviewer for his/her positive comments.

Responses to reviewer #1:

Line 87 Rephrase "urea concentration in renal" perhaps renal urea concentration or urine urea concentration in kidney

**Reply:** We thank the reviewer for his/her suggestion. We have rephrased the "urea concentration in renal" to "urine urea concentration in kidney" at line 85.

Intro revision OK

**Reply:** We thank the reviewer for his/her positive comments.

**Results**:

Comment 1. OK ;

**Reply:** We thank the reviewer for his/her positive comments.

Comment 2. OK ;

**Reply:** We thank the reviewer for his/her positive comments.

Comment3. OK ;

**Reply:** We thank the reviewer for his/her positive comments.

Comment 4. OK

**Reply:** We thank the reviewer for his/her positive comments.

Comment 5. No changes required.

**Reply:** We thank the reviewer for his/her positive comments.

Comment 6. Nice addition, OK ;

**Reply:** We thank the reviewer for his/her positive comments.

Comment 7.OK

**Reply:** We thank the reviewer for his/her positive comments.

**Discussion**

Comment 8. Need to correct type-o's or wording in some places, ie., physiological instead of physiologically and little vs litter. lines 359 - 361)

**Reply:** We thank the reviewer for his/her positive comments. We have corrected the spell errors at line 357-359.

Comment 9. Adequate although the response mentions that MDCK cells were not able to be effectively transfected with UT-A2, this was not apparently added to the text. This is not of sufficient importance to require additional experimentation.

**Reply:** We thank the reviewer for his/her positive comments. We have added the explanation of lacking UT-A2 transfected MDCK cell-based assay in the methods at line 599-601 as follows: "*Considering the rigorous urease reaction assay using 293F cell-based system and the difficulty of UT-A2 expression in MDCK cell, we did not assess the response of the inhibitors on UT-A2 in the MDCK-based assay.*".

Comment 10. Interesting addition and worth adding

**Reply:** We thank the reviewer for his/her positive comments.

**Figures and Legends.**

Comment 11. OK ;

**Reply:** We thank the reviewer for his/her positive comments.

Comment 12. OK :

**Reply:** We thank the reviewer for his/her positive comments.

Comment 13..1E. OK ; 13.2 G. OK ; 13.3 OK

**Reply:** We thank the reviewer for his/her positive comments.

Comment 14. OK ;

**Reply:** We thank the reviewer for his/her positive comments.

Comment 15. OK

**Reply:** We thank the reviewer for his/her positive comments.

Comment 16. 16.1 OK 16.2 Change 'Papillary'to 'the papilla; 16.3 OK; 16.4 OK

**Reply:** We thank the reviewer for his/her positive comments. We have changed “Papillary” to “the papilla” at line 1015.

Suggest double checking the wording on the figure legends.

**Reply:** We thank the reviewer for his/her suggestions. We have checked the wording on the figure legends again and corrected those related to figure changes.

Overall, nice job in responding to this reviewer’s queries.

**Reply:** We thank the reviewer for his/her positive comments.

Reviewer #2 (Remarks to the Author) Authors have mostly addressed my concerns. They have also markedly expanded the manuscript with additional analysis and literature review.

**Reply:** We thank the reviewer for his/her positive comments.

Only a few minor comments:

1. Discussion section is now much longer. If the journal format allows, it will be great to split it into subsections.

**Reply:** We thank the reviewer for his/her suggestions. We appreciate the reviewer’s comment regarding splitting the Discussion section into subsections. However, after careful consideration, we believe that maintaining the current structure helps preserve the logical flow and coherence of the discussion. Therefore, we have kept the Discussion section as a single continuous section.

2. Several citations are still missing, particularly in the methods section. For example, revision has led to UCSF Chimera being mentioned earlier, but it is not cited there (line 677).

**Reply:** We thank the reviewer for his/her suggestions. We have added the citation of UCSF Chimera at line 689.

3. Some figures are still a bit difficult to read. For example, Fig. 3g has some residues in light

brown (e.g. A326) which makes them illegible. Similar with panels Fig. 4a and 5f.

**Reply:** We thank the reviewer for his/her suggestions. We have revised the figures of Fig3g, Fig 4a, and Fig 5f to improve readability. In details, the residue color of SCG pocket, including A326, was changed to yellow in the Figure R1(also Fig 3g). And the color of compound label was changed to black in Figure R2(also Fig 4a). In addition, the residue color of "T-T" sub-pocket was changed to magenta in Figure R3(also Fig 5f).

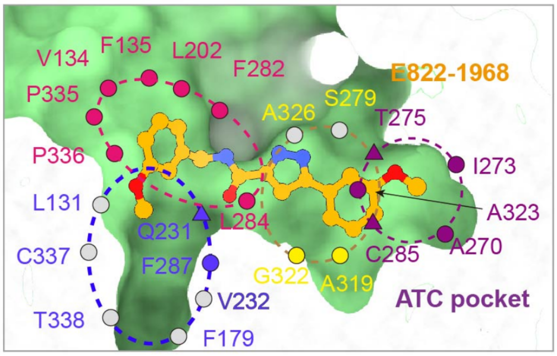


Figure R1. Cutaway view of the E822-1968 binding pockets in UT-A2. E822-1968 occupied the EBBP (extracellular blocker binding pocket), the SCG pocket and the ATC pocket, represented by magentas, yellow and purple dashed line circles respectively. Residues forming polar interactions or hydrogen bonds with E822-1968 are depicted using triangles. Residues forming hydrophobic or van der Waals interactions are depicted as solid round circles, while residues that do not interact with E822-1968 are shown as gray circles.

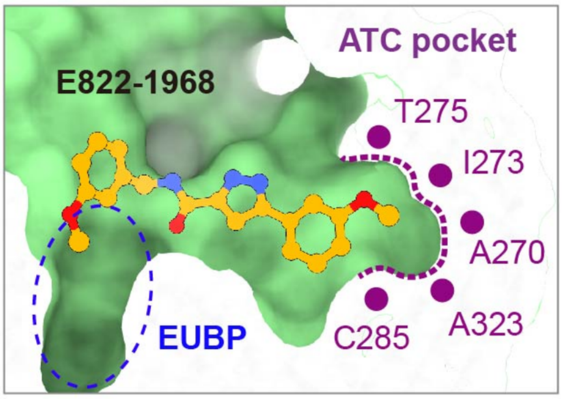


Figure R2. Cutaway view of the ATC pocket and the EUBP (extracellular urea binding pocket).

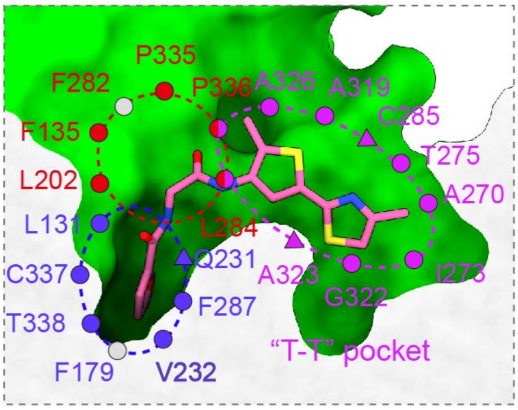


Figure R3. The cutaway views of binding pockets of M353-0039 in UT-A2. The furan moiety of M353-0039 occupies the EUBP, forming critical interactions for urea transport inhibition (highlighted by blue dashed circles). The EBBP is demarcated by red dashed circles, while the thiazole-thiophene moiety engages the "T-T" subpocket (magenta dashed circles). Residues forming polar interactions or hydrogen bonds with M353-0039 are depicted using triangles. Residues only forming hydrophobic or van der Waals interactions are depicted as solid dots. The residues that have no interaction with M353-0039 are shown as gray dots.

Reviewer #3 (Remarks to the Author)

The points raised in the previous round of review were addressed and I have no further concerns regarding this manuscript.

**Reply:** We thank the reviewer for his/her positive comments.

Reviewer #4 (Remarks to the Author)

Almost all comments have been addressed sufficiently. However, some minor comments still needs reworking:

**Reply:** We thank the reviewer for his/her positive comments.

1.) (2.) Docking validation): For visual clarity, please ensure that the ROC curve originates at the origin, i.e. remove the whitespace at the bottom and on the left, and move the 0s for x and y to the origin.

**Reply:** We thank the reviewer for his/her suggestions. We have revised the ROC curve originates in Figure R4 (also Supplementary Fig 7a), removed the whitespace at the bottom and on the left, and moved the 0s for x and y to the origin.

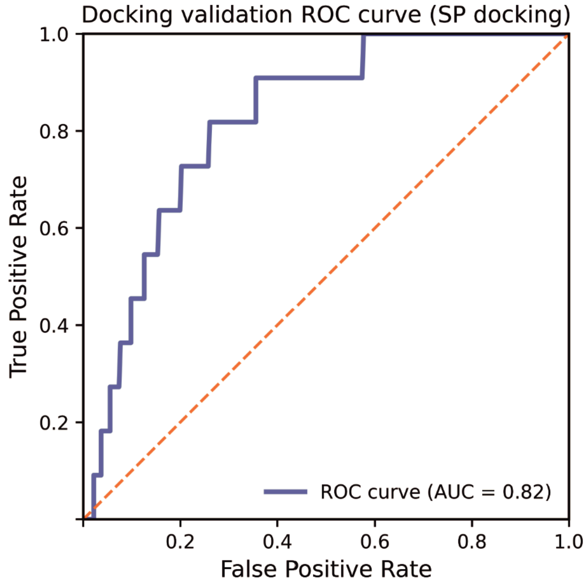


Figure R4. Receiver operating characteristic (ROC) curve evaluating the performance of SP docking in distinguishing active compounds from decoys. The area under the curve (AUC) is 0.82, indicating good discriminatory power of the docking protocol

2.) (10. Figure 4 - Chemical Structures): When creating figures containing 2D depictions of molecules, please refer to the style guidelines (e.g. problematic bond angles in HQA2 and many other molecules) (https://www.nature.com/documents/nr-chemical-structures-guide.pdf). ChemDraw,for example, allows you to 'clean up' the structure.

**Reply:** We thank the reviewer for his/her suggestions. We have carefully revised all 2D molecule structures according to the recommended style guidelines using ChemDraw software, and the bond lengths and bond angles were corrected via the “clean-up structure” function (Figure R5). Further, we have revised the 2D molecule structure of E822-1968 in Figure R6 (also Fig 1d), HQA2 and E822-1968 in Figure R7 (also Fig 2g), E3 in Figure R8 (also Fig 4c), M353-0039, D400-1574, E762-1894, 8012-3374, D103-1163 in Figure R9 (also Fig 4e). And we have updated the presentation of residue interaction with E822-1968 in Figure R10 (also Fig3f), and the presentation of residue interaction with M353-0039 in Figure R11(also Fig 5k).

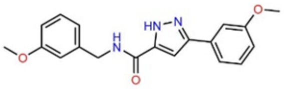

E822-1968

3-(3-methoxyphenyl)-N-[(3-methoxyphenyl)methyl]-1H pyrazole-5-carboxamide

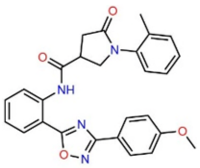

D400-1574

N-{2-[3-(4-methoxyphenyl)-1,2,4-oxadiazol-5-yl]phenyl}-1-(2-methylphenyl)-5-oxopyrrolidine-3-carboxamide

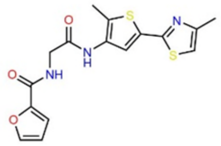

M353-0039

N-(2-{[2-methyl-5-(4-methyl-1,3-thiazol-2-yl)thiophen-3-yl]amino}-2-oxoethyl)furan-2-carboxamide

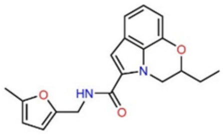

E762-1894

2-ethyl-N-[(5-methylfuran-2-yl)methyl]-2,3-dihydro[1,4]oxazino [2,3,4-hi]indole-5-carboxamide

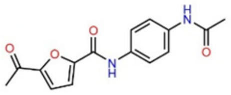

25a

N-(4-acetamidophenyl)-5-acetylfuran-2-carboxamide

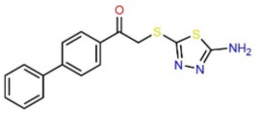

8012-3374

2-[(5-amino-1,3,4-thiadiazol-2-yl)sulfanyl]-1-([1,1'-biphenyl]-4-yl)ethan-1-one

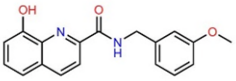

HQA2

8-hydroxy-N-(3-methoxybenzyl)quinoline-2-carboxamide

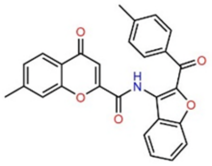

D103-1163

7-methyl-N-[2-(4-methylbenzoyl)-1-benzofuran-3-yl]-4-oxo-4H-1-benzopyran-2-carboxamide

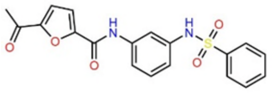

E3

5-acetyl-N-[3-(phenylsulfonamido)phenyl]furan-2-carboxamide

Figure R5. All chemical structures presented in the article have been re-drawn with ChemDraw.

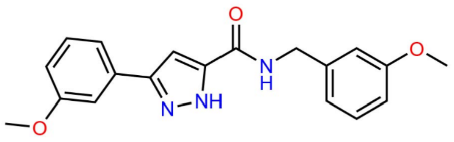

Compound E822-1968

$IC_{50}$(UT-A2) = 0.44 ± 0.03 μM

Figure R6. The molecular formula of compound E822-1968 which shows inhibitory potency toward UT-A2 with the IC50 = 0.44 ± 0.03 μM.

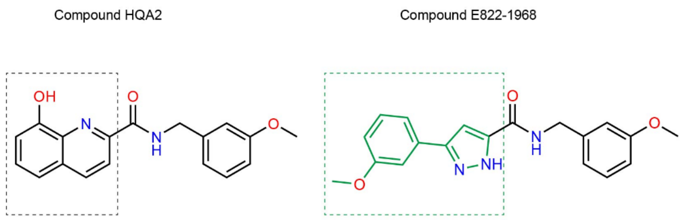


Figure R7. Structural formula comparison between E822-1968 and the previously reported UT-A inhibitor HQA2. The difference between the two compounds lies in the replacement of the hydroxyquinoline moiety in HQA2 with a pyrazole-anisole moiety of E822-1968.

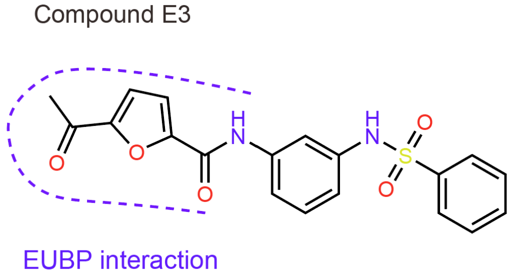


Figure R8. Diagram of compound E3 engaging with EUBP with the interfaces represented by blue dashed lines.

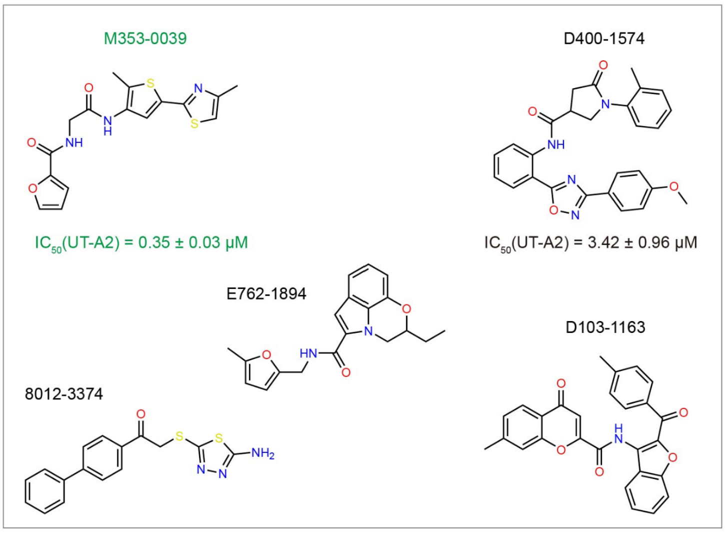


Figure R9. Structural formulas of the top five candidate compounds for UT-A2 selective inhibitors in progressive rescreening. The compounds M353-0039 and D400-1574 showed inhibitory potency to UT-A2 with IC50 value of 0.35 ± 0.03 μM and 3.42 ± 0.96 μM.

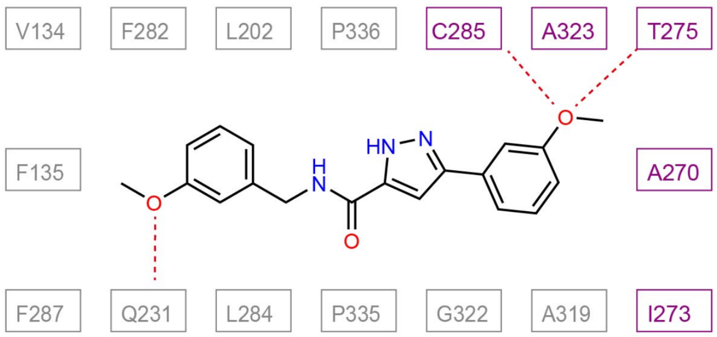


Figure R10. A diagram shows the residues in the E822-1968 binding pocket of UT-A2. The hydrogen bonds and polar interactions are shown as red dashed lines. The residues involved in the ATC pocket are colored by purple.

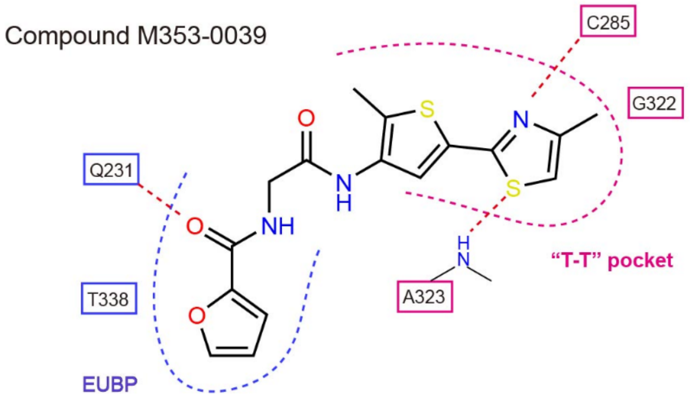


Figure R11. The diagram of M353-0039 engaging with the extracellular urea binding pocket (EUBP), "T-T" pocket with the interfaces represented by blue and violet dashed lines, respectively. Key residues with EUBP and "T-T" pocket are shown in blue and violet rectangles, respectively. The hydrogen bonds and polar interactions are shown as red dash line.
